# Cerebellar subregional structural changes across the Alzheimer’s disease continuum: a longitudinal analysis of cognitive and behavioural correlates

**DOI:** 10.1093/braincomms/fcaf500

**Published:** 2025-12-23

**Authors:** Yoo Hyun Um, Sheng-Min Wang, Dong Woo Kang, Sunghwan Kim, Suhyung Kim, Donghyeon Kim, Yeong Sim Choe, Regina E Y Kim, Hyun Kook Lim

**Affiliations:** Department of Psychiatry, St. Vincent’s Hospital, College of Medicine, The Catholic University of Korea, Seoul 06591, Republic of Korea; Department of Psychiatry, Yeouido St. Mary’s Hospital, College of Medicine, The Catholic University of Korea, Seoul 06591, Republic of Korea; Department of Psychiatry, Seoul St. Mary’s Hospital, College of Medicine, The Catholic University of Korea, Seoul 06591, Republic of Korea; Department of Psychiatry, Yeouido St. Mary’s Hospital, College of Medicine, The Catholic University of Korea, Seoul 06591, Republic of Korea; Department of Psychiatry, St. Vincent’s Hospital, College of Medicine, The Catholic University of Korea, Seoul 06591, Republic of Korea; Research Institute, Neurophet Inc., Seoul 06234, Republic of Korea; Research Institute, Neurophet Inc., Seoul 06234, Republic of Korea; Research Institute, Neurophet Inc., Seoul 06234, Republic of Korea; Department of Psychiatry, Yeouido St. Mary’s Hospital, College of Medicine, The Catholic University of Korea, Seoul 06591, Republic of Korea

**Keywords:** Alzheimer’s disease, cerebellum, neuropsychiatric symptoms, episodic memory, cortico-cerebellar networks

## Abstract

Although traditionally viewed as relatively spared in Alzheimer’s disease (Ad), the cerebellum is increasingly recognized for its contribution to cognitive and behavioural dysfunction. However, longitudinal data delineating subregional cerebellar involvement across the Ad continuum remain limited. In this study, we investigated longitudinal cerebellar atrophy and its clinical correlates in 259 older adults classified via amyloid PET into four biomarker-defined groups: cognitively normal controls, preclinical Ad, Ad-related mild cognitive impairment and Ad dementia. Structural MRI data were analysed using the Spatially Unbiased Infratentorial Template (SUIT), and longitudinal changes in 28 cerebellar subregions were assessed via generalized estimating equations, controlling for demographic and biological covariates. Across longitudinal analyses, cerebellar structural alterations in preclinical Ad were closely associated with both cognitive and behavioural measure changes. Reductions in lobule VI and Crus I/II were correlated with episodic memory decline, emphasizing the cerebellum’s contributions to early cognitive deterioration. The same regions were involved in associations with apathy and behavioural dysregulation, suggesting the cerebellar contribution to emerging neuropsychiatric symptoms through disruption of motivational and executive circuits. In addition, stage-dependent cortico-cerebellar coupling was noted, with coordinated volume loss between cerebellar lobule VI and temporo-orbitofrontal cortices in the preclinical stage, but selective posterior cerebellar-posterior cingulate synchrony in dementia, indicating progressive network reorganization and eventual decoupling along the disease continuum. This study provides the first biomarker-defined longitudinal mapping of cerebellar subregional atrophy in Ad. The findings demonstrate that cerebellar degeneration is not confined to advanced stages but emerges early and dynamically interacts with cortical networks, influencing both cognitive decline and neuropsychiatric symptoms. The distinct atrophy patterns and cortico-cerebellar decoupling underscore the cerebellum’s potential as a disease-stage–specific biomarker and therapeutic target in Ad.

## Teaser

Um *et al*. report that cerebellar degeneration in Alzheimer’s disease begins early, with subregional atrophy in lobule VI and Crus I/II during the preclinical stage and widespread loss in dementia. These longitudinal findings reveal cortico-cerebellar interactions underlying cognitive and neuropsychiatric symptoms, suggesting the cerebellum as an early biomarker and therapeutic target.

## Graphical Abstract

**Graphical Abstract:**
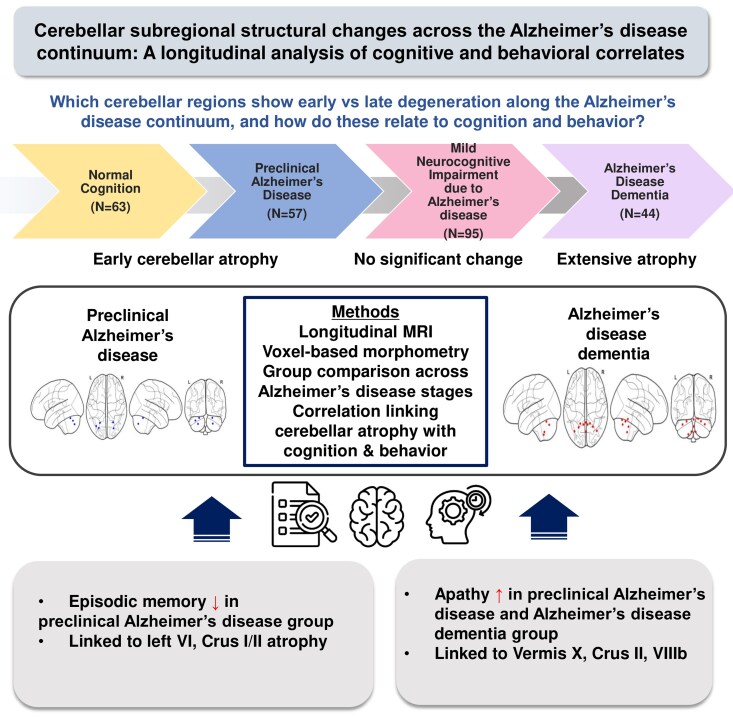

## Introduction

Alzheimer’s disease (Ad) is a gradually progressive neurodegenerative disease that leads to cognitive deterioration and behavioural dysfunction.^1,2^ While hippocampal and cortical atrophy are well-established hallmarks, the mechanisms underlying Ad-related cognitive and behavioural dysfunction remain incompletely understood.^3^ The cerebellum, long regarded as primarily involved in motor control has recently been recognized as playing a role in cognitive functions such as executive control, emotion regulation and visuospatial skills, as illustrated by cerebellar cognitive affective syndrome (CCAS).^4^ Disruptions in cortico-cerebellar networks have been implicated in episodic memory impairment, suggesting the cerebellum’s involvement in Ad-related symptoms.^5^

Despite this, the cerebellum has been considered relatively resistant to Ad pathology. Compared to the cerebrum, it shows less beta-amyloid (Aβ) and tau deposition and more stable grey matter volume and is often used as a reference region in amyloid and tau PET imaging.^6-8^ Moreover, although Aβ deposition does occur in the cerebellum, tau pathology typically observed in the cerebrum appears largely sparse.^9,10^ However, this notion has been challenged by recent histopathological studies showing both Aβ plaques and phosphorylated tau in the cerebellar cortex in early-onset AD.^11^ Structural MRI studies have also shown that the cerebellum is not entirely spared along the Ad continuum. Cross-sectional and longitudinal studies report cerebellar volume loss, especially in posterior regions, during later stages of Ad.^12-14^

More refined analyses reveal cerebellar vulnerability even earlier. A voxel-based morphometry study using cerebellum-specific templates found grey matter loss in amnestic mild cognitive impairment patients, particularly in the vermis and lobules I–V and VI14. In Ad dementia, posterior cerebellar atrophy was more prominent, although less severe than cortical atrophy.^12,13^ A longitudinal study further reported that total cerebellar atrophy mainly contributes to disease progression in late stages, with minimal changes in prodromal phases until conversion is imminent.^12^ Mild cognitive impairment patients who converted to Ad dementia had an elevated annual atrophy rate of 0.6% (64% higher than normal), whereas stable mild cognitive impairment patients had rates similar to cognitively normal individuals (0.4%/year).^12^

Nevertheless, important gaps remain. First, previous longitudinal studies did not define disease stages based on biomarkers, limiting sensitivity to early changes. Our prior work revealed altered functional connectivity between the locus coeruleus (LC) and cerebellum in preclinical Ad, suggesting possible compensatory structural reorganization or Wallerian degeneration linked to early pathology.^13^ Second, although cross-sectional studies emphasize subregional cerebellar volume loss over total volume loss in prodromal Ad,^14^ no longitudinal study has yet confirmed these findings. Third, the relationship between cerebellar structural changes throughout the entire course of Ad and associated cognitive-behavioural impairments—specifically, the progression of CCAS—remains unclear. Due to these gaps, there might be still a significant discrepancy between cognitive-behavioural symptoms, biomarker alterations and cerebellar changes in Ad.

To address these gaps, our study investigated longitudinal changes in cerebellar volumes across four diagnostic groups: normal cognition, preclinical Ad, mild cognitive impairment due to Ad and Ad dementia. Using MRI and PET imaging analyses, we employed a generalized estimating equation (GEE) to assess cerebellar volume changes over time. We hypothesized distinct longitudinal patterns across groups and examined correlations between cerebellar changes, cognitive performance and structural alterations in the cerebrum. By focusing on these relationships, we aimed to clarify the cerebellum’s involvement in Ad progression and its potential as an early biomarker.

## Materials and methods

### Study design and participants

This longitudinal study included 259 older adults (≥60 years) recruited from the Catholic Aging Brain Imaging Database (CABID) between 2018 and 2023. All participants underwent structural MRI, amyloid PET and standardized neuropsychological and neuropsychiatric assessments. Participants underwent baseline and 2-year follow-up structural MRI and clinical assessments. Four biomarker-defined diagnostic groups were established according to the NIA-AA framework: healthy control (HC), preclinical Ad (PAD), mild cognitive impairment due to Ad (MCI_Ad) and Ad dementia (ADD). All participants were memory clinic visitors presenting with memory complaints, including those with subjective cognitive decline (SCD) who nevertheless performed within the normal range on standardized neuropsychological testing. Diagnostic classification details and inclusion/exclusion criteria are provided in Supplementary Method S1. The study size was determined by the availability of participants with complete longitudinal structural MRI, PET and neuropsychological data in the CABID cohort between 2018 and 2023. Apolipoprotein ε4 (APOE4) genotyping was performed for all participants. (Supplementary Method S2). No formal power calculation was performed, as all eligible individuals meeting inclusion criteria were included. The study was conducted in accordance with the ethical and safety standards of the local Institutional Review Board of the Catholic University of Korea and the Declaration of Helsinki. All participants provided written informed consent.

### Imaging data acquisition and processing

T1-weighted structural MRI and amyloid PET scans were acquired and processed using validated pipelines. MRI preprocessing included segmentation using Statistical Parametric Mapping version 12 (SPM12; Wellcome Department of Cognitive Neurology, London, UK) with the Computational Anatomy Toolbox version 12 (CAT12; C. Gaser, Structural Brain Mapping Group, Jena University Hospital, Germany)^15^ and cerebellar parcellation via Spatially Unbiased Infratentorial Template (SUIT).^16^ The cerebellar regions analysed in this study, as defined by the SUIT atlas, encompass 28 distinct areas that provide a comprehensive representation of cerebellar anatomy. These regions include left I-IV, right I-IV, left V, right V, left VI, vermis VI, right VI, left Crus I, vermis Crus I, right Crus I, left Crus II, vermis Crus II, right Crus II, left VIIb, vermis VIIb, right VIIb, left VIIIa, vermis VIIIa, right VIIIa, left VIIIb, vermis VIIIb, right VIIIb, left IX, vermis IX, right IX, left X, vermis X and right X. T1-weighted structural MRI images were processed, and cortical volumes were calculated using the FreeSurfer image analysis suite (version 6.0, http://surfer.nmr.mgh.harvard.edu), following methods detailed in previously published studies.^17,18^ The regions of interest (ROIs) selected for this study—frontal lobe, temporal lobe, posterior cingulate cortex (PCC), hippocampus and insula.^19-21^ For each ROI, both the left hemisphere (denoted as ‘lh’) and the right hemisphere (denoted as ‘rh’) structures were analysed to capture lateralized effects and ensure comprehensive coverage of the brain regions.^22-24^ All segmented images obtained from CAT12 SUIT and FreeSurfer were visually inspected for accuracy. Quality control was performed independently by two trained raters (a psychiatrist specialized in dementia and a neuroimaging analyst with extensive experience in MRI processing). Participants with major segmentation errors or with significant structural abnormalities unrelated to Ad (e.g. large territorial infarcts, tumours) were excluded from the analyses. Global and regional amyloid standardized uptake value ratios (SUVRs) were calculated using automated segmentation software powered by a deep-learning model (SCALE PET v. 0.1.3.1, developed by Neurophet, South Korea).^25^ Detailed scanner parameters, acquisition protocols, region of interest and preprocessing information are reported in Supplementary Method S3.

### Cognitive and neuropsychiatric measures

Cognitive function was assessed using the Korean version of the Consortium to Establish a Registry for Alzheimer’s Disease Assessment Packet (CERAD-K).^26^Composite scores were computed for episodic and non-episodic memory. Neuropsychiatric symptoms were evaluated using the Neuropsychiatric Inventory (NPI),^27^ with 12 domains grouped into four categories based on previous factor structures (mood, psychotic, hyperactivity and behavioural symptoms). Test composition and scoring details are presented in Supplementary Method S4.

### Statistical analyses

Missing data were minimal. All MRI, PET and neuropsychological variables had complete data, with the exception of a single missing observation for the NPI. Given that this represented <1% of the dataset and occurred completely at random, the case was excluded via listwise deletion. No imputation procedures were necessary, and all analyses were performed on the remaining complete cases. Analysis of covariance (ANCOVA) was used to examine baseline differences in cerebellar regional volumes across diagnostic groups, with age, sex, years of education, APOE4 carrier status and total intracranial volume (TIV) included as covariates. For group-wise comparisons following significant omnibus results in ANCOVA models, *post hoc* analyses were conducted using Tukey’s Honest Significant Difference (HSD) test. This approach was chosen to control for Type I error across all pairwise comparisons while preserving statistical power. Group differences in regional cerebellar volumes were assessed across HC, PAD, MCI_Ad and ADD groups using this method. The significance level was set at *P* < 0.05 (two-tailed), and adjusted *P*-values from the HSD procedure were reported to ensure rigorous control of multiple testing. Longitudinal changes in cerebellar volumes over the 2-year follow-up period were analysed using GEE, allowing for repeated measures within subjects and adjusting for the same covariates as in the ANCOVA. For each cerebellar region, we modelled time (baseline versus follow-up) as a within-subject variable and examined interaction effects with diagnosis.

To assess the relationships between cerebellar structural changes and clinical as well as cortical measures, we performed a series of partial correlation analyses. First, Spearman’s rank-order correlation coefficients were calculated between longitudinal changes in cerebellar volumes and changes in neuropsychiatric symptoms and cognitive performance. Second, to examine cross-structural associations, we computed partial correlations between changes in cerebellar ROIs and frontal/temporal cortical ROIs derived from the FreeSurfer-based FSF parcellation.

To estimate partial correlations, we first regressed out covariates including age, sex, years of education, APOE4 carrier status and TIV from both the cerebellar and behavioural/cortical variables. Correlation coefficients were then computed between the residuals of the two variables of interest, yielding Spearman’s rho (r). To account for multiple comparisons, we applied the Benjamini–Hochberg false discovery rate (FDR) procedure. Families of tests were defined by outcome domains; for example, ‘all cerebellar ROIs × one NPI subscale’, ‘all cerebellar ROIs × CERAD-K episodic memory’, ‘all cerebellar ROIs × CERAD-K non-episodic memory’ and ‘all cerebellar ROIs × one FreeSurfer cortical ROI’ were each treated as separate families for FDR adjustment. For each family, uncorrected two-tailed *P*-values were converted into FDR-adjusted *q*-values. Both *P*-values and *q*-values are reported. Correlations were considered nominally significant at *P* < 0.05 and significant after correction if *q* < 0.05.

To examine whether baseline Aβ burden predicts longitudinal cerebellar atrophy, we constructed linear regression models for each cerebellar region within the PAD, MCI_Ad and ADD groups. The dependent variable was the change in regional cerebellar volume (Δ volume) over the observation period. The primary independent variable was baseline global cortical amyloid burden, measured using the global SUVR from amyloid PET. Each model additionally included baseline regional cerebellar volume, age, sex, years of education, APOE4 carrier status and TIV as covariates.

All statistical analyses were conducted in Python (version 3.9.7) using the statsmodels, scipy and pandas libraries. Full details of the statistical models, assumptions, covariate structures and software packages are provided in Supplementary Method S5.

## Results

### Baseline demographic and clinical characteristics

Demographic and clinical characteristics of the participants are summarized in Table 1. There were no significant group differences in age or TIV. However, groups differed significantly in years of education, APOE4 carrier frequency, amyloid burden and cognitive performance across all CERAD-K domains. The ADD group showed the lowest cognitive scores, while the HC and PAD groups performed similarly.

**Table 1:** Demographic and clinical characteristics of participants (*n* = 259)

	HC (*n* = 63)	PAD (*n* = 57)	MCI_Ad (*n* = 95)	ADD (*n* = 44)	*P*-value
**Age (years ± SD)**	73.46 ± 7.36	72.89 ± 6.95	75.73 ± 6.60	74.77 ± 9.53	0.093
**Education (years ± SD)**	11.22 ± 5.48	10.30 ± 5.14	10.44 ± 5.68	7.91 ± 5.06	0.017
**Sex (Female, %)**	76.19	64.91	68.42	79.55	0.292
**APOE4 carrier (Yes, %)**	9.52	45.61	51.58	59.09	<0.001
**Total intracranial volume (mm^3^** **±** **SD)**	1418.84 ± 119.45	1444.77 ± 131.00	1422.41 ± 128.54	1407.25 ± 138.17	0.505
**Regional SUVR_PONS_ (SUVR** **±** **SD)**					
**ACC**	0.45 ± 0.11	0.66 ± 0.10	0.72 ± 0.11	0.78 ± 0.14	<0.001
**FL**	0.40 ± 0.04	0.56 ± 0.12	0.64 ± 0.13	0.72 ± 0.11	<0.001
**PL**	0.35 ± 0.05	0.47 ± 0.10	0.55 ± 0.11	0.63 ± 0.11	<0.001
**PCC/precuneus**	0.52 ± 0.05	0.68 ± 0.14	0.82 ± 0.14	0.92 ± 0.12	<0.001
**TL**	0.48 ± 0.04	0.59 ± 0.10	0.68 ± 0.11	0.76 ± 0.12	<0.001
**Global SUVR _PONS_ (SUVR±** **SD)**	0.48 ± 0.07	0.64 ± 0.10	0.73 ± 0.10	0.79 ± 0.10	<0.001
**CERAD-K Battery (mean** **±** **SD)**					
**VF**	14.95 ± 4.56	15.04 ± 4.45	11.18 ± 3.66	8.02 ± 3.47	<0.001
**BNT**	12.02 ± 2.36	12.18 ± 2.13	10.02 ± 2.75	8.34 ± 2.79	<0.001
**MMSE**	27.24 ± 2.62	27.33 ± 2.59	23.89 ± 3.94	17.36 ± 4.42	<0.001
**WLM**	18.11 ± 4.16	17.61 ± 4.06	13.37 ± 3.36	7.80 ± 2.73	<0.001
**CP**	10.02 ± 1.26	10.19 ± 1.26	9.61 ± 1.81	7.93 ± 2.37	<0.001
**WLR**	6.16 ± 1.75	6.00 ± 1.60	2.55 ± 1.67	0.57 ± 0.95	<0.001
**WLRc**	9.22 ± 0.99	9.11 ± 1.05	6.46 ± 2.43	4.00 ± 2.25	<0.001
**CR**	6.81 ± 2.89	6.68 ± 2.98	2.85 ± 2.70	0.64 ± 1.56	<0.001

HC, healthy control; Ad, Alzheimer’s disease; PAD, preclinical Alzheimer’s disease; MCI_Ad, mild cognitive impairment due to Alzheimer’s disease; ADD, Alzheimer’s disease dementia; SD, standard deviation; APOE, apolipoprotein E; CERAD-K, Korean version of the Consortium to Establish a Registry for Alzheimer’s Disease; VF, verbal fluency; BNT, 15-item Boston Naming Test; MMSE, Mini Mental Status Examination; WLM, word list memory; CP, constructional praxis; WLR, word list recall; WLRc, word list recognition; CR, constructional recall; SUVRPONS, standardized uptake value ratios of [18F] flutemetamol with pons as the reference region; ACC, anterior cingulate cortex; FL, frontal lobes; PL, parietal lobes; PCC, posterior cingulate cortex; TL, lateral temporal lobes.

### Baseline cerebellar volume reductions across diagnostic groups

**Figure 1:**
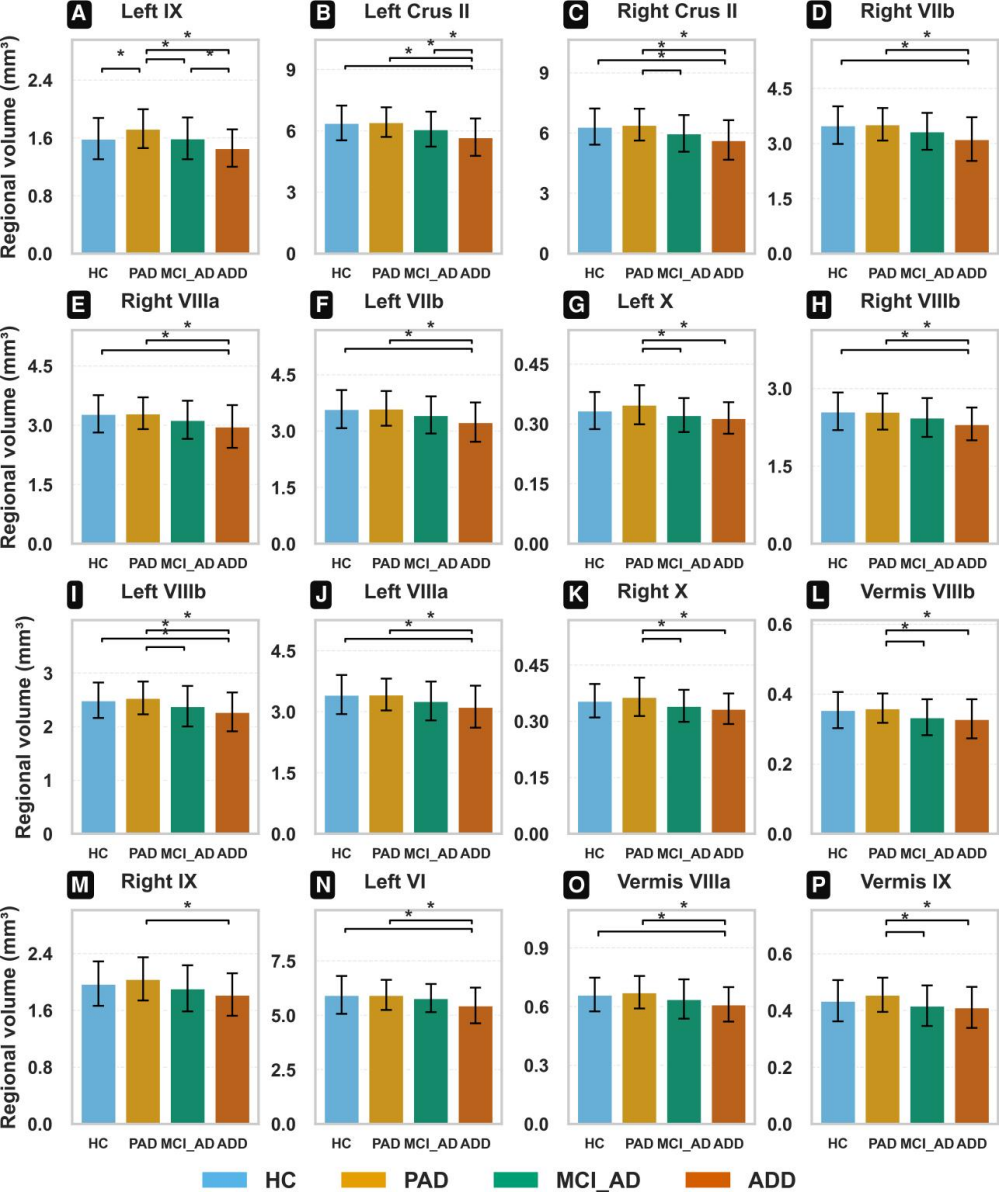
**Baseline group comparisons of regional cerebellar volumes (mm³) adjusted for covariates (age, sex, education, TIV and APOE4 carrier status), showing only regions with significant differences.** Experimental unit: individual participant. Sample sizes: HC *n* = 62, PAD *n* = 57, MCI_Ad  *n* = 95, ADD *n* = 44. Statistical analysis: Group differences were tested using ANCOVA adjusted for age, sex, years of education, TIV and APOE4 carrier status. Error bars represent standard errors of the mean. Significant Tukey’s HSD pairwise contrasts (*P*) are listed when applicable. (**A**) Left IX—ANCOVA *F*(3, 249) = 7.26, *P*=<0.001. (**B**) Left Crus I—ANCOVA *F*(3, 249) = 6.46, *P*=<0.001. (**C**) Right Crus II—ANCOVA *F*(3, 249) = 5.65, *P*=<0.001. (**D**) Right VIIb—ANCOVA *F*(3, 249) = 5.32, *P* = 0.001. (**E**) Right VIIIa—ANCOVA *F*(3, 249) = 4.65, *P* = 0.003. (**F**) Left VIIb—ANCOVA *F*(3, 249) = 4.40, *P* = 0.005. (**G**) Left X—ANCOVA *F*(3, 249) = 4.22, *P* = 0.006. (**H**) Right VIIIb—ANCOVA *F*(3, 249) = 4.05, *P* = 0.008. (**I**) Left VIIIb—ANCOVA *F*(3, 249) = 3.83, *P* = 0.010. (**J**) Left VIIIa—ANCOVA *F*(3, 249) = 3.67, *P* = 0.013. (**K**) Right X—ANCOVA *F*(3, 249) = 3.61, *P* = 0.014. (**L**) Vermis VIIIb—ANCOVA *F*(3, 249) = 3.44, *P* = 0.017. (**M**) Right IX—ANCOVA *F*(3, 249) = 3.33, *P* = 0.020. (**N**) Left VI—ANCOVA *F*(3, 249) = 3.23, *P* = 0.023. (**O**) Vermis VIIIa—ANCOVA *F*(3, 249) = 3.18, *P* = 0.025. (**P**) Vermis IX—ANCOVA *F*(3, 249) = 3.05, *P* = 0.029. Abbreviations: TIV, total intracranial volume; APOE4, apolipoprotein ε4; ANCOVA, analysis of covariance; HC, healthy control; PAD, preclinical Alzheimer’s disease; MCI_Ad, mild cognitive impairment due to Alzheimer’s disease; ADD, Alzheimer’s disease dementia.

ANCOVA identified significant group differences in 16 cerebellar regions (Fig. 1). The ADD group exhibited the most pronounced reductions in bilateral Crus I and II, lobules VI, VIIIb and IX. Detailed ANCOVA statistics for all 28 cerebellar subregions, including pairwise post-hoc comparisons, are provided in Supplementary Tables S1 and S2.

### Longitudinal changes in cerebellar volumes across the Ad spectrum

**Figure 2:**
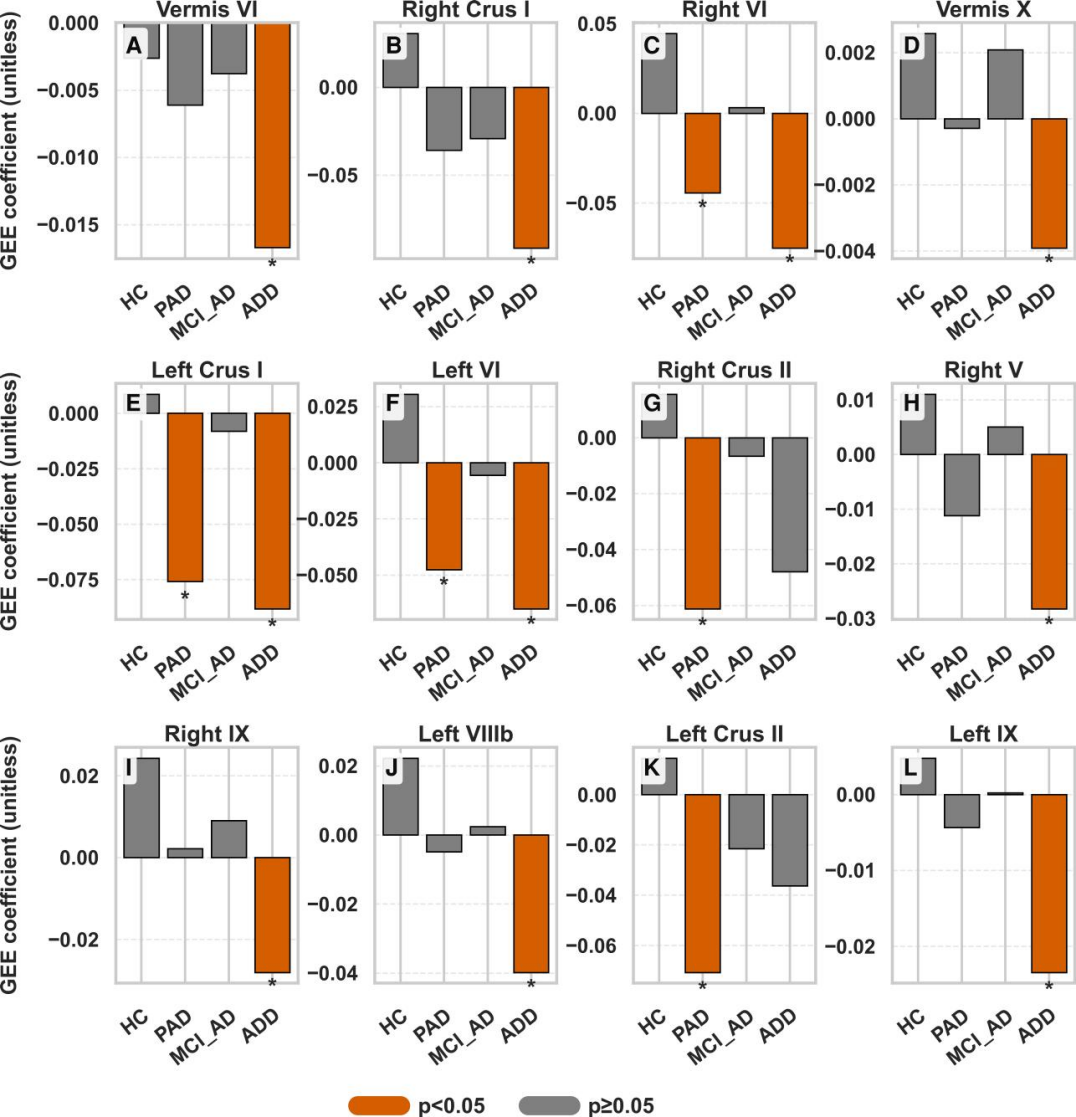
**Longitudinal changes in regional cerebellar volumes across the Alzheimer’s disease trajectory, showing only regions with significant longitudinal changes.** Experimental unit: individual participant. Bar show group-specific GEE coefficients; asterisks indicate statistically significant results (*P* < 0.05). Statistical analysis: GEE adjusted for age, sex, APOE4 carrier status, TIV and education. *P*-values are reported for each subregion. GEE models assessed cerebellar volume change over time, controlling for age, sex, years of education, APOE4 carrier status and TIV. The MCI_Ad group exhibited no significant longitudinal change, suggesting a plateau before renewed atrophy in ADD. Error bars represent 95% confidence intervals. (**A**) Vermis VI—HC: β=−0.003, *P* = 0.784; PAD: β=−0.006, *P* = 0.109; MCI_Ad: β=−0.004, *P* = 0.179; ADD: β=−0.017, *P*=<0.001. (**B**) Right Crus I—HC: β=0.031, *P* = 0.652; PAD: β=−0.036, *P* = 0.142; MCI_Ad: β=−0.029, *P* = 0.265; ADD: β=−0.091, *P* = 0.002. (**C**) Right VI—HC: β=0.045, *P* = 0.435; PAD: β=−0.044, *P* = 0.005; MCI_Ad: β=0.003, *P* = 0.825; ADD: β=−0.075, *P* = 0.003. (**D**) Vermis X—HC: β=0.003, *P* = 0.188; PAD: β=−0.000, *P* = 0.791; MCI_Ad: β=0.002, *P* = 0.075; ADD: β=−0.004, *P* = 0.007. (**E**) Left Crus I—HC: β=0.009, *P* = 0.883; PAD: β=−0.076, *P* = 0.031; MCI_Ad: β=−0.008, *P* = 0.795; ADD: β=−0.088, *P* = 0.011. (**F**) Left VI—HC: β=0.030, *P* = 0.615; PAD: β=−0.048, *P* = 0.012; MCI_Ad: β=−0.006, p = 0.694; ADD: β=−0.065, *P* = 0.021. (**G**) Right Crus II—HC: β=0.016, *P* = 0.740; PAD: β=−0.061, *P* = 0.013; MCI_Ad: β=−0.007, *P* = 0.816; ADD: β=−0.048, *P* = 0.101. (**H**) Right V—HC: β=0.011, *P* = 0.653; PAD: β=−0.011, *P* = 0.141; MCI_Ad: β=0.005, *P* = 0.520; ADD: β=−0.028, *P* = 0.027. (**I**) Right IX—HC: β=0.024, *P* = 0.263; PAD: β=0.002, *P* = 0.840; MCI_Ad: β=0.009, *P* = 0.392; ADD: β=−0.028, *P* = 0.030. (**J**) Left VIIIb—HC: β=0.022, *P* = 0.279; PAD: β=−0.005, *P* = 0.677; MCI_Ad: β=0.002, *P* = 0.813; ADD: β=−0.040, *P* = 0.030. (**K**) Left Crus II—HC: β=0.014, *P* = 0.799; PAD: β=−0.071, *P* = 0.033; MCI_Ad: β=−0.022, *P* = 0.343; ADD: β=−0.036, p = 0.272. (**L**) Left IX— HC: β=0.005, *P* = 0.783; PAD: β=−0.004, *P* = 0.587; MCI_Ad: β=−0.000, *P* = 0.979; ADD: β=−0.023, *P* = 0.0.39. Abbreviations: GEE, generalized estimating equation; APOE4, apolipoprotein ε4; TIV, total intracranial volume; HC, healthy control; PAD, preclinical Alzheimer’s disease; MCI_Ad, mild cognitive impairment due to Alzheimer’s disease; ADD, Alzheimer’s disease dementia.

**Figure 3:**
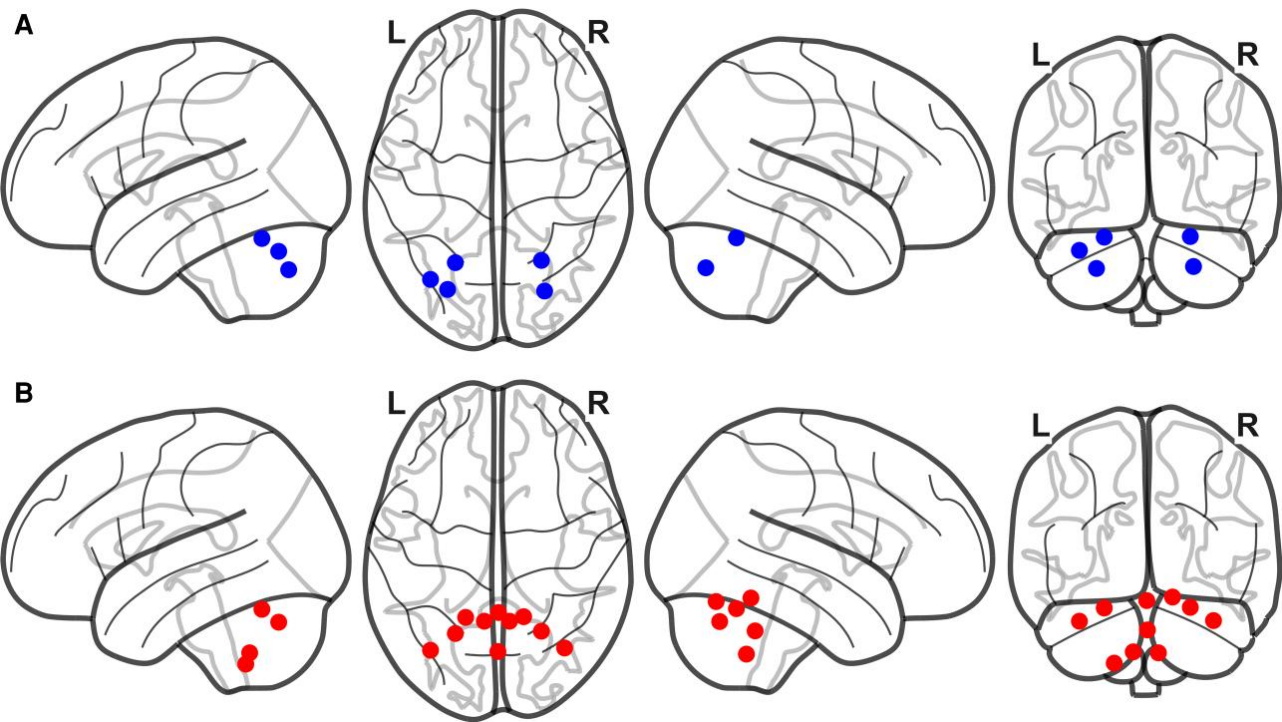
**Regional cerebellar atlas highlighting significant longitudinal volume differences observed in PAD and ADD groups.** (**A**): PAD (blue nodes); B: ADD (red nodes). Each node represents the MNI centroid of a SUIT cerebellar region that showed a significant longitudinal decrease in volume in the GEE models. Experimental unit: individual participant. (PAD: *n* = 57; ADD: *n* = 44). Statistical analysis: Statistical details for each region (regression coefficient, standard error, *P*-value) are reported in Supplementary Table S4–S7. Abbreviations: PAD, preclinical Alzheimer’s disease; ADD, Alzheimer’s disease dementia; MNI, Montreal Neurological Institute; SUIT, Spatially Unbiased atlas for the cerebellum and brainstem, GEE, generalized estimating equation.

GEE analysis revealed distinct longitudinal patterns of cerebellar volume decline. In the PAD group, early atrophy was detected in left VI (*β* = −0.05, *P* = 0.012), right VI (*β* = −0.04, *P* = 0.005), left Crus I (*β* = −0.08, *P* = 0.031) and left Crus II (*β* = −0.06, *P* = 0.013). In contrast, the ADD group demonstrated more extensive and accelerated atrophy, notably in right Crus I (*β* = −0.091, *P* = 0.002), left Crus I (*β* = −0.088, *P* = 0.011) and vermis X. Figure 2 shows volume trajectories across selected regions and Fig. 3 illustrates cerebellar regions where significant longitudinal volume differences were observed. Comprehensive GEE results for all cerebellar subregions in each diagnostic group are available in Supplementary Tables S4–S7.

In addition to region-specific findings, whole cerebellar volume changes were also examined. (Supplementary Table S3). Over the 2-year follow-up, the ADD group exhibited significant reductions in total cerebellar volume (*β* = −0.600, *P* = 0.015) and cerebellar grey matter volume (*β* = −0.737, *P* = 0.018), while no significant changes were observed in cerebellar white matter volume. Similarly, the PAD group showed a significant decline in cerebellar grey matter volume (*β* = −0.425, *P* = 0.041), but not in total volume or white matter. In contrast, the HC group showed no significant longitudinal changes across cerebellar regions, whereas the MCI_Ad group demonstrated a plateau pattern without further decline, potentially reflecting compensatory mechanisms before renewed atrophy in ADD.

### Partial correlations between longitudinal changes in cerebellar volumes and CERAD-K composite scores

**Figure 4:**
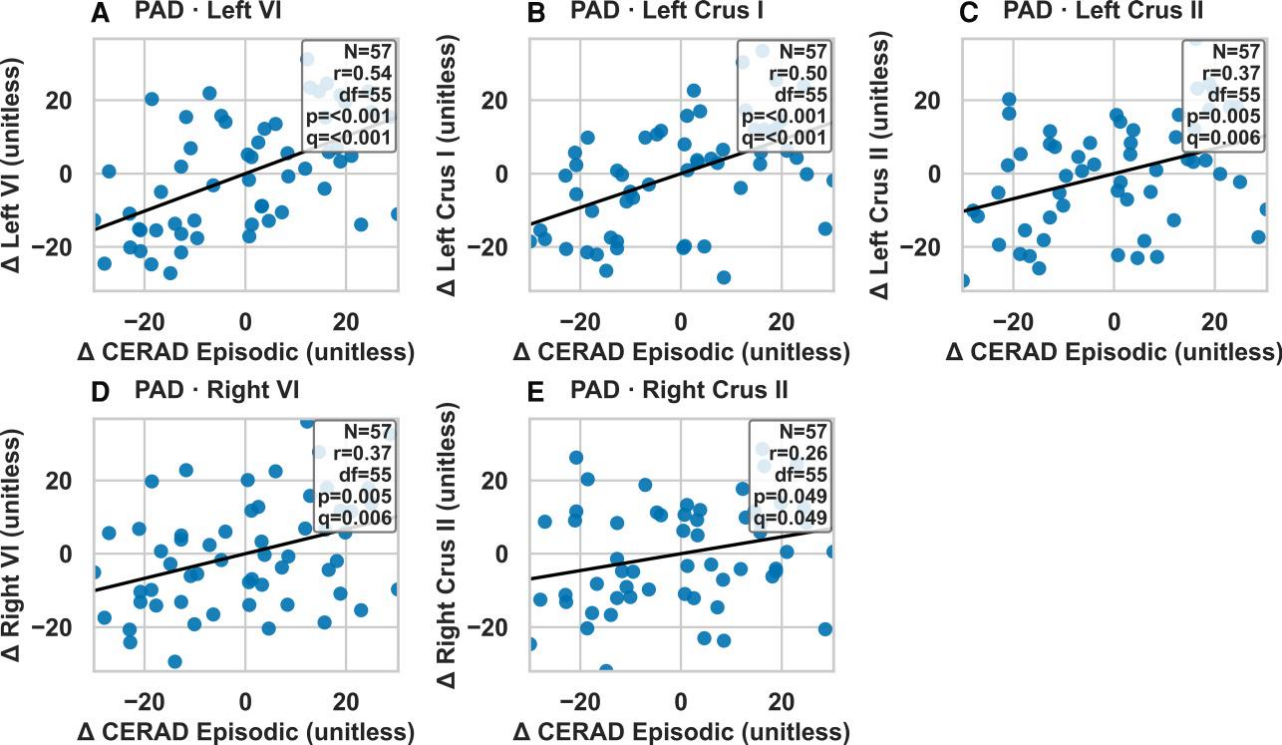
**Correlations between longitudinal cerebellar volume changes (mm³) and CERAD-K score changes in PAD group, adjusted for covariates (age, sex, education, TIV, APOE4 carrier status).** Panels depict significant partial Spearman correlations between longitudinal changes (Δ) in cerebellar subregional volumes and changes in CERAD-K Non-Episodic or CERAD-K Episodic memory scores, after adjusting for age, sex, education, APOE ε4 carrier status and TIV. Each panel (**A–E**) corresponds to a specific cerebellar region and diagnostic group. Regression lines represent the fitted partial correlation trend, and shaded points represent individual participants. Experimental unit: The experimental unit is an individual participant. Each dot corresponds to one participant’s residualized change score. Statistical analysis: Partial Spearman correlations (rank-based partial Pearson correlations) were computed between Δ CERAD-K and Δ cerebellar ROI values while controlling for covariates listed above. For each panel, the number of participants (*n*), correlation coefficient (*r*), degrees of freedom (df = *n* − 2), uncorrected *P*-value and FDR-adjusted q-value (Benjamini–Hochberg correction within each Group × Measure family) are reported. Axes and units: *x*-axes indicate Δ CERAD-K score (either Non-Episodic or Episodic) and *y*-axes indicate Δ volume in the corresponding cerebellar region. Abbreviations: PAD, preclinical Alzheimer’s disease; CERAD-K, Consortium to Establish a Registry for Alzheimer’s Disease–Korean version; APOE4, apolipoprotein E4; TIV, total intracranial volume; FDR, false discovery rate.

In the PAD group, partial correlation analysis revealed robust associations between reductions in cerebellar volume and episodic memory decline. Specifically, changes in the left VI (*r* = 0.54, *P* < 0.001, *q* < 0.001), left Crus I (*r* = 0.50, *P* < 0.001, *q* < 0.001), left Crus II (*r* = 0.37, *P* = 0.005, *q* = 0.006), right VI (*r* = 0.37, *P* = 0.005, *q* = 0.006) and right Crus II (*r* = 0.26, *P* = 0.049, *q* = 0.049) were positively correlated with changes in episodic memory composite scores. These findings remained significant after false discovery rate correction. Figure 4 illustrates the key associations, and full correlation matrices are provided in Supplementary Tables S8 and S9.

### Partial correlations between longitudinal changes in cerebellar volumes and NPI domains

**Figure 5:**
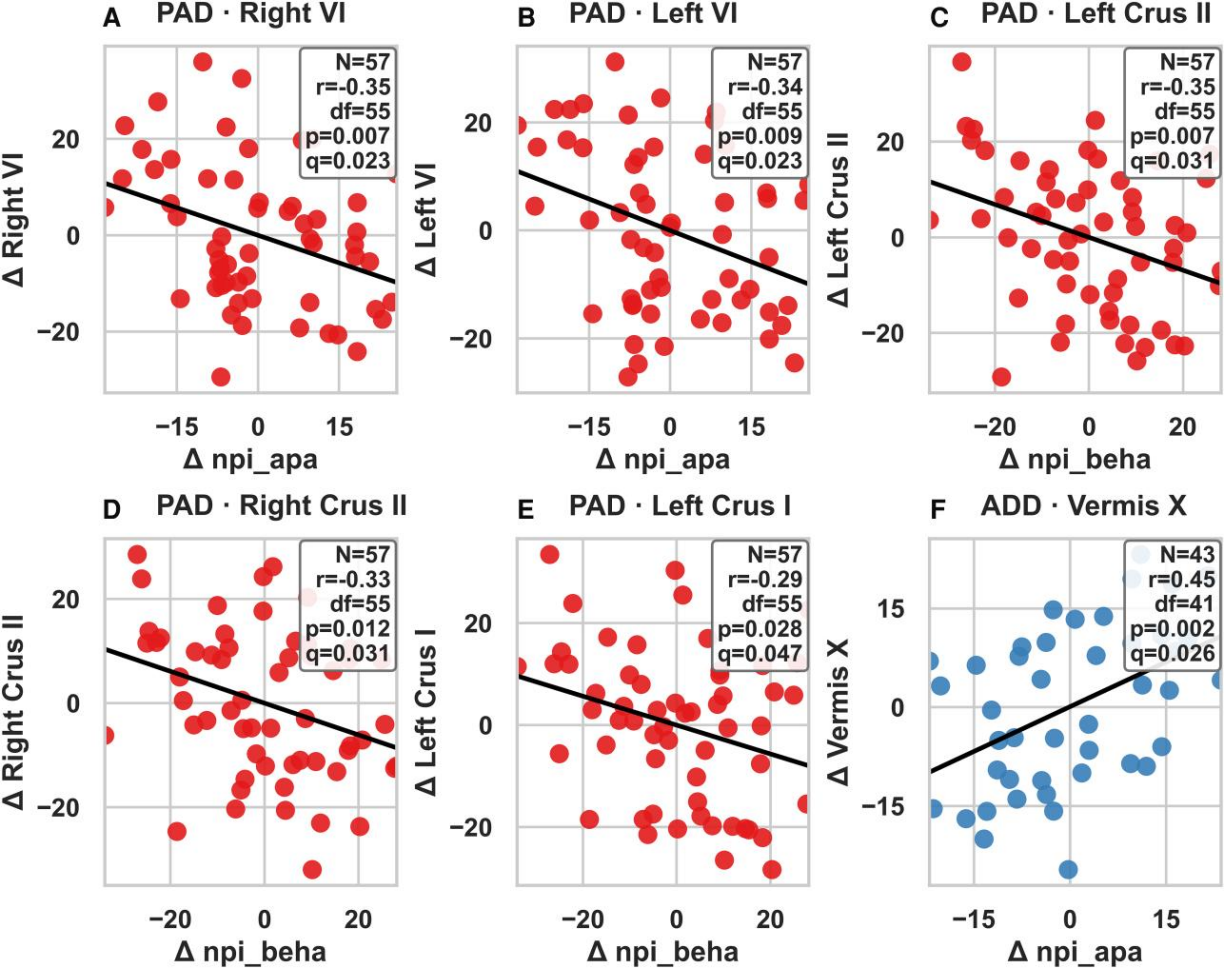
**Correlations between longitudinal cerebellar volume changes (mm³) and NPI score changes in PAD and ADD, adjusted for covariates (age, sex, education, TIV, APOE4 carrier status).** Panels (**A–F**) show significant (or top-ranked) partial Spearman correlations between longitudinal changes (Δ) in NPI scores and cerebellar subregional volumes, adjusting for age, sex, education, APOE4 carrier status and TIV. Experimental unit: The experimental unit is an individual participant. Each dot corresponds to one participant’s residualized change score. Statistical analysis: Partial Spearman correlations were computed as rank-based partial for each panel, we report *n*, correlation coefficient (*r*), degrees of freedom (df = *n* − 2), uncorrected *P*-value and FDR-adjusted q-value (Benjamini–Hochberg within each NPI family, separately for PAD and ADD). Axes and units: *x*-axes indicate Δ NPI score, and *y*-axes indicate Δ volume in the corresponding cerebellar region. Abbreviations: PAD, preclinical Alzheimer’s disease; ADD, Alzheimer’s disease dementia; NPI, Neuropsychiatric Inventory; APOE4, apolipoprotein E4; TIV, total intracranial volume; FDR, false discovery rate.

After adjusting for age, sex, education, APOE4 status and TIV, several cerebellar–behavioural associations remained significant following FDR correction. In the PAD group, greater changes in right VI (*r* = −0.35, *P* = 0.007, *q* = 0.023) and left VI (*r* = −0.34, *P* = 0.009, *q* = 0.023) were associated with changes in the apathy domain, and changes in left Crus II (*r* = −0.35, *P* = 0.007, *q* = 0.031), right Crus II (*r* = −0.33, *P* = 0.012, *q* = 0.031) and left Crus I (*r* = −0.29, *P* = 0.028, *q* = 0.047) were associated with behavioural dysregulation. In the ADD group, Vermis X changes were significantly associated with apathy (*r* = 0.46, *P* = 0.002, *q* = 0.026). Figure 5 illustrates representative associations; full matrices and uncorrected results are provided in Supplementary Tables S10–S11.

### Longitudinal links between cerebellar atrophy and cortical–limbic systems in Ad

In the PAD group, multiple significant positive associations between cerebellar lobule VI and cortical regions survived FDR correction. Specifically, reductions in left VI correlated with parallel volume decreases in the left inferior temporal gyrus (*r* = 0.57, *q* < 0.001), left superior temporal gyrus (*r* = 0.44, *q* = 0.004) and left medial orbitofrontal cortex (*r* = 0.40, *q* = 0.009). Similar synchrony was observed for right VI, which showed positive correlations with the right inferior temporal gyrus (*r* = 0.39, *q* = 0.013), right parahippocampal gyrus (*r* = 0.36, *q* = 0.015) and right superior temporal gyrus (*r* = 0.35, *q* = 0.017). These findings indicate that during the preclinical phase, cortico-cerebellar networks, particularly involving temporal and orbitofrontal regions, undergo coordinated volume loss.

In the ADD group, significant correlations emerged between posterior cerebellar lobules and the posterior cingulate cortex. For instance, Δ right V was positively associated with Δ volume in the left posterior cingulate cortex (*r* = 0.46, *q* = 0.016) and the right posterior cingulate cortex (*r* = 0.41, *q* = 0.029). Additionally, Δ right VI correlated with Δ volume in the right posterior cingulate cortex (*r* = 0.41, *q* = 0.029). Full correlation matrices are available in Supplementary Tables S12 and S13.

### Baseline Aβ burden and longitudinal cerebellar atrophy

Across PAD, MCI_Ad and ADD groups, regression models tested whether baseline amyloid burden predicted longitudinal cerebellar atrophy after adjusting for baseline regional volume, age, sex, education, APOE4 status and TIV. After false discovery rate correction, no associations survived in any group or region (all q > 0.05) (Supplementary table S14–S16).

## Discussion

This study examined cerebellar volume changes, cognitive function and neuropsychiatric symptoms across the Ad spectrum. Key findings include the following: (i) group differences in cerebellar volumes, (ii) longitudinal volume decline in PAD and ADD, (iii) associations with cognitive performance in PAD, (iv) links with neuropsychiatric symptoms in PAD and ADD and (v) correlations with cortical and limbic regions over time. This is the first region-specific longitudinal analysis of cerebellar atrophy across the Ad spectrum, revealing early structural changes often missed in prior research. By integrating PET-based biomarker classification with volumetric and clinical data, this approach offers a more precise, biologically grounded view of cerebellar involvement in Ad progression.

### Cerebellar volume changes across the Ad spectrum

The observed volume reductions in specific cerebellar regions, such as Crus I, Crus II and lobule VIIb, are particularly noteworthy.^28^ These areas have been implicated in cognitive functions beyond motor control.^28^ Our findings reveal a complex trajectory of cerebellar atrophy, with the ADD group showing the lowest volumes, consistent with progressive degeneration in Ad. Significant volume reductions in Crus I, Crus II and lobule VIIb align with their roles in executive functions, memory and language processing, as well as their integration within the default mode network (DMN) and frontoparietal network.^29^ The lateral ‘limbic’ cerebellar circuits, encompassing Crus I and II, further connect with prefrontal and subcortical regions, supporting their contributions to diverse cognitive and emotional processes.^30,31^ This progression supports the notion that cerebellar atrophy may precede or occur concurrently with cortical changes in Ad. Supporting this, Samstag *et al*. identified significant reductions in granular layer volume and VGlut1+ glomeruli in the lateral cerebellar cortex during Braak stages IV–VI.^32^

Interestingly, atrophy in vermis regions such as VIIIb, Crus II and IX correlated with reductions in Purkinje cells and dendritic spine density,^33^ extending our understanding of cerebellar contributions beyond cognitive lobules. In line with this, previous studies using SHAP (Shapley Additive Explanations) analysis have identified specific radiomic features in Crus I/II and vermis lobules I–III as key markers for distinguishing MCI_Ad from cognitively normal individuals.^34^ This suggests that structural changes in these cerebellar regions may have diagnostic potential in the early stages of Ad and highlights the broader involvement of the cerebellum in Ad pathology.

### Differential longitudinal cerebellar volume changes across the Ad spectrum

GEE analyses revealed distinct longitudinal patterns, with significant atrophy in PAD in regions like left VI, right VI, right Crus I and left Crus II, suggesting that cerebellar degeneration begins in PAD. Our results diverge from previous findings that suggested cerebellar atrophy occurs primarily in later Ad stages.^12^ By analysing specific lobules, we identified early cerebellar volume reductions in PAD, particularly in Crus I, Crus II and lobule VIIb. This suggests a more active role for the cerebellum in Ad pathophysiology. These findings highlight the cerebellum’s early vulnerability, even before significant cortical and hippocampal atrophy becomes evident. The absence of significant changes in MCI_Ad, followed by renewed atrophy in ADD, implies a non-linear trajectory of cerebellar involvement. This stabilization in MCI_Ad may reflect compensatory mechanisms aimed at preserving cognitive function during this transitional stage.

One potential driver of these compensatory processes is the LC, a critical hub in neurodegeneration that exhibits extensive functional connectivity with the cerebellum. The LC modulates both cognitive and motor functions through its influence on widespread brain networks, including the salience network (SN) and DMN.^35,36^ Structural covariance studies have shown that disruptions in LC–cerebellar connectivity emerge early in Ad, particularly in PAD and MCI_Ad.^37^Given its role in regulating attention and executive function, the cerebellum may act as a bridge in these circuits, helping to offset early neurodegenerative changes. This interaction between the LC and cerebellum may provide a temporary buffering effect, delaying overt cognitive decline until the disease progresses further.

In ADD, widespread cerebellar atrophy was observed, extending beyond the early-affected regions identified in PAD. Significant volume reductions were particularly notable in the right and left Crus I, right and left lobule VI and additional involvement of right and left lobule IX, vermis VI and vermis X. These regions are associated with key cognitive and motor networks, including the DMN, FPN and somatomotor network.^38^ This progressive degeneration pattern suggests a breakdown of initially compensatory cortico-cerebellar circuits, leading to disrupted communication across distributed functional networks. The involvement of both lateral hemispheric (e.g. Crus I) and vermal regions (e.g. lobule IX and X) highlights the cerebellum’s dual role in motor and cognitive processes and its vulnerability in the later stages of Alzheimer’s disease. These findings underscore the value of cerebellar markers in understanding the network-level degeneration characteristic of advanced Ad pathology.

### Cerebellar volume changes and cognitive performance

The strong correlation between cerebellar volume changes and CERAD-K composite scores in PAD underscores the cerebellum’s involvement in early cognitive decline. Bilateral VI volume changes, in particular, showed significant associations with episodic memory, extending prior findings of VI atrophy in aMCI to the preclinical stage,^14^ our findings extend this timeline by demonstrating that structural changes in these regions are evident in the preclinical phase. Moreover, observed correlation between changes in the left VI region and episodic memory performance, despite prior studies emphasizing the right cerebellum’s role in memory,^29,39^ may reflect nuanced, task-dependent cerebello-cerebral dynamics. Episodic memory tasks, particularly those involving visuospatial or associative components, could engage the left cerebellum through contralateral connections with right cortical regions, such as the hippocampus.^40,41^ The left VI region, acting as a transition zone between anterior and posterior networks, may compensate for disruptions in right-sided networks during early Ad.^42^ This transitional role may enable it to compensate for disruptions in right-sided networks in preclinical phase of Ad, particularly given its contralateral connections to cortical areas involved in episodic memory.

The significant correlation between the bilateral Crus II, left Crus I, and episodic memory in PAD is in line with previous findings where the Crus region was associated with episodic memory decline in Ad.^13,36^ Our observation of Crus I/II involvement is consistent with recent precision mapping showing that this region constitutes a ‘cognitive megacluster’ with multiple, interdigitated subregions supporting language, social cognition, episodic memory and cognitive control.^43^ The positive correlations between Crus I/II atrophy and episodic memory decline in PAD may therefore reflect the selective vulnerability of memory-related subzones within this cluster. These findings suggest that early episodic memory impairment in Ad is not solely driven by hippocampal or cortical pathology but also involves degeneration of cerebellar subregions tightly coupled to memory networks, with Crus I/II decline serving as a potential marker of disrupted cortico-cerebellar synchrony.

### Cerebellar volume changes and neuropsychiatric symptoms

In the PAD group, volume reductions in lobule VI bilaterally were significantly associated with increased apathy, while changes in Crus I and Crus II (bilateral) correlated with worsening behavioural dysregulation. These findings reinforce the view that Crus I/II are integral to social cognition, executive control and emotion regulation, consistent with meta-analytic evidence implicating these regions in mentalizing and affective processes.^44^ The early emergence of these associations in PAD highlights that neuropsychiatric vulnerability may arise before overt dementia through disruptions of cortico-cerebellar circuits supporting motivation and behavioural regulation.

In the ADD group, atrophy of vermis X was significantly associated with apathy. The vermis has long been linked to limbic regulation and affective modulation,^45^ and its degeneration may directly contribute to diminished motivation and emotional blunting in advanced disease. Together, these results provide converging evidence that degeneration of specific cerebellar subregions underlies distinct behavioural and psychological symptoms across the Ad trajectory. They further support the concept that the cerebellum contributes not only to cognitive decline but also to the neuropsychiatric symptomatology of Ad, through disruption of cerebello-limbic and cerebello-frontal circuits.

### Longitudinal associations with cortical and limbic regions

Our findings reveal stage-dependent shifts in cortico-cerebellar coupling. In PAD, lobule VI changes were correlated with temporal and orbitofrontal cortical atrophy, suggesting early vulnerability of associative cortico-cerebellar networks. In contrast, in ADD, significant associations were confined to posterior midline structures, specifically between posterior cerebellar lobules and the posterior cingulate cortex (PCC). This may seem paradoxical, as the PCC is among the earliest cortical regions affected in Ad and a well-established hub of DMN.^46^ One interpretation is that by the dementia stage, widespread cortico-cerebellar coordination has already collapsed, leaving only the most resilient or persistently interconnected networks detectable. The persistence of PCC–cerebellar correlations in ADD may therefore reflect a residual coupling of DMN–cerebellar circuits, serving as a ‘last link’ of synchrony before complete network disintegration. This interpretation is consistent with recent dynamic multilayer functional connectivity studies, which showed that alterations in DMN and limbic networks track both preclinical and clinical stages of Ad and that network flexibility and integration decline most prominently along the Ad continuum.^43^ Thus, while PCC dysfunction is an early hallmark of Ad, its enduring connectivity with posterior cerebellar regions in advanced disease may represent either selective resilience or a marker of the network’s final breakdown.^47,48^

Taken together, these results suggest that cortico-cerebellar synchrony is broadly distributed across associative networks during the preclinical phase but becomes progressively restricted to posterior midline circuits in advanced Ad, highlighting both the dynamic reorganization and eventual collapse of network-level connectivity across the disease trajectory.

### Link between baseline amyloid load and progressive cerebellar volume loss

In the present study, higher baseline global amyloid burden did not predict subsequent cerebellar volume decrease across the Ad spectrum. The overall findings suggest that cerebellar atrophy progresses largely independently of baseline Aβ deposition, implying that Aβ accumulation alone may not drive cerebellar degeneration. Instead, cerebellar involvement may reflect downstream or parallel mechanisms related to tau pathology, neuroinflammation, or network disconnection as disease severity increases.

### Limitations and future directions

While our study provides valuable insights into cerebellar involvement in Ad, several limitations should be noted. First, the cross-sectional nature of some analyses limits causal interpretation. Second, reliance on structural MRI alone restricts understanding of the functional impact of cerebellar changes. Future studies incorporating both structural and functional neuroimaging could offer a more comprehensive view. Third, although a 2-year follow-up aligns with prior studies in PAD,^49,50^ it may be insufficient to capture long-term trajectories. Given the slow progression of neurodegeneration, longer follow-up with multiple time points is needed to clarify cerebellar involvement and its potential as a biomarker. Fourth, the short follow-up may limit differentiation between converters and non-converters. Fifth, the absence of tau PET or blood-based tau markers precluded direct assessment of tau pathology. Sixth, our cohort did not include participants with MCI who were Aβ negative. As a result, it remains uncertain whether the observed findings are specific to Ad-related pathology. Considering tau-related changes beyond cortical regions, future integration of tau biomarkers is essential. Seventh, potential confounding from medications and comorbidities should be acknowledged. Eighth, although we used established segmentation methods (SPM12/CAT12 with SUIT parcellation), these traditional approaches are limited by partial volume effects due to the fine architecture of the cerebellum.^51^ More recent deep learning–based pipelines such as CerebNET may offer superior sensitivity to subtle volumetric changes and could help refine longitudinal estimates.^51^ Finally, inclusion of detailed clinical and post-mortem data would further elucidate cerebellar changes in Ad. Our findings underscore the cerebellum’s dynamic role across the Ad continuum, with its atrophy and associations with cognitive and neuropsychiatric symptoms highlighting its potential as a biomarker and therapeutic target.

## Supplementary Material

fcaf500_Supplementary_Data

## Data Availability

The datasets generated or analysed during the current study are not publicly available due to the patient data management protocol of Yeouido St. Mary’s Hospital but may be obtained from the corresponding author upon reasonable request. All Python codes used for data preprocessing, statistical analyses and figure generation are provided in the Supplementary material (Supplementary Data 1: Analysis Codes).

## References

[1] Li XL, Hu N, Tan MS, Yu JT, Tan L. Behavioural and psychological symptoms in Alzheimer’s disease. Biomed Res Int. 2014;2014:927804. 25133184 10.1155/2014/927804 PMC4123596

[2] Zhang J, Zhang Y, Wang J, Xia Y, Zhang J, Chen L. Recent advances in Alzheimer’s disease: Mechanisms, clinical trials and new drug development strategies. Signal Transduct Target Ther. 2024;9(1):211. 39174535 10.1038/s41392-024-01911-3 PMC11344989

[3] Selles MC, Oliveira MM, Ferreira ST. Brain inflammation connects cognitive and non-cognitive symptoms in Alzheimer’s disease. J Alzheimers Dis. 2018;64(Suppl 1):S313–S327. 29710716 10.3233/JAD-179925

[4] Argyropoulos GPD, van Dun K, Adamaszek M, et al The cerebellar cognitive affective/Schmahmann syndrome: A task force paper. Cerebellum. 2020;19(1):102–125. 31522332 10.1007/s12311-019-01068-8 PMC6978293

[5] Geissmann L, Coynel D, Papassotiropoulos A, de Quervain DJF. Neurofunctional underpinnings of individual differences in visual episodic memory performance. Nat Commun. 2023;14(1):5694. 37709747 10.1038/s41467-023-41380-w PMC10502056

[6] Betthauser TJ, Cody KA, Zammit MD, et al In vivo characterization and quantification of neurofibrillary tau PET radioligand 18F-MK-6240 in humans from Alzheimer disease dementia to young controls. J Nucl Med. 2018;60:93–99. 29777006 10.2967/jnumed.118.209650 PMC6354223

[7] Tissot C, Servaes S, Lussier FZ, et al The association of age-related and off-target retention with longitudinal quantification of [18F]MK6240 tau PET in target regions. J Nucl Med. 2022;64:452–459. 36396455 10.2967/jnumed.122.264434 PMC10071794

[8] Vemuri P, Lowe VJ, Knopman DS, et al Tau-PET uptake: Regional variation in average SUVR and impact of amyloid deposition. Alzheimers Dement (Amst). 2016;6:21–30. 28138510 10.1016/j.dadm.2016.12.010 PMC5257031

[9] Braak H, Braak E, Bohl J, Lang W. Alzheimer’s disease: Amyloid plaques in the cerebellum. J Neurol Sci. 1989;93:277–287. 2556503 10.1016/0022-510x(89)90197-4

[10] Mann DMA, Jones D, Prinja D, Purkiss MS. The prevalence of amyloid (A4) protein deposits within the cerebral and cerebellar cortex in Down’s syndrome and Alzheimer’s disease. Acta Neuropathol. 2004;80:318–327. 10.1007/BF00294651 1698007

[11] Sepulveda-Falla D, Matschke J, Bernreuther C, et al Deposition of hyperphosphorylated tau in cerebellum of PS1 E280A Alzheimer’s disease. Brain Pathol. 2011;21(4):452–463. 21159009 10.1111/j.1750-3639.2010.00469.x PMC8094246

[12] Tabatabaei-Jafari H, Walsh E, Shaw ME, Cherbuin N; for the Alzheimer’s Disease Neuroimaging Initiative. The cerebellum shrinks faster than normal ageing in Alzheimer’s disease but not in mild cognitive impairment. Hum Brain Mapp. 2017;38(6):3141–3150. 28321950 10.1002/hbm.23580 PMC5426955

[13] Chen Y, Spina S, Callahan P, et al Pathology-specific patterns of cerebellar atrophy in neurodegenerative disorders. Alzheimers Dement. 2024;20(3):1771–1783. 38109286 10.1002/alz.13551 PMC10984510

[14] Toniolo S, Serra L, Olivito G, Marra C, Bozzali M, Cercignani M. Patterns of cerebellar grey matter atrophy across Alzheimer’s disease progression. Front Cell Neurosci. 2018;12:430. 30515080 10.3389/fncel.2018.00430 PMC6255820

[15] Gaser C, Dahnke R, Thompson P, Kurth F, Luders E. CAT – a computational anatomy toolbox for the analysis of structural MRI data. 2022. Gigascience. 2024;13:giae049. 39102518 10.1093/gigascience/giae049 PMC11299546

[16] Diedrichsen J . A spatially unbiased atlas template of the human cerebellum. Neuroimage. 2006;33(1):127–138. 16904911 10.1016/j.neuroimage.2006.05.056

[17] Fischl B . FreeSurfer. Neuroimage. 2012;62(2):774–781. 22248573 10.1016/j.neuroimage.2012.01.021 PMC3685476

[18] Lee H, Kim HW, Lee M, et al Evaluating brain volume segmentation accuracy and reliability of FreeSurfer and Neurophet AQUA at variations in MRI magnetic field strengths. Sci Rep. 2024;14(1):24513. 39424856 10.1038/s41598-024-74622-y PMC11489576

[19] Kesslak JP, Nalcioglu O, Cotman CW. Quantification of magnetic resonance scans for hippocampal and parahippocampal atrophy in Alzheimer’s disease. Neurology. 1991;41(1):51. 1985296 10.1212/wnl.41.1.51

[20] Zhu Y, Wu Y, Lv X, et al The relationship between APOE genotype, CSF tau and cognition across the Alzheimer’s disease spectrum: Moderation and mediation role of insula network connectivity. CNS Neurosci Ther. 2024;30(1):e14401. 37577852 10.1111/cns.14401 PMC10805399

[21] Fennema-Notestine C, Hagler DJ Jr, McEvoy LK, et al Structural MRI biomarkers for preclinical and mild Alzheimer’s disease. Hum Brain Mapp. 2009;30(10):3238–3253. 19277975 10.1002/hbm.20744 PMC2951116

[22] Liu H, Zhang L, Xi Q, et al Changes in brain lateralisation in patients with mild cognitive impairment and Alzheimer’s disease: A resting-state functional magnetic resonance study from the Alzheimer’s disease neuroimaging initiative. Front Neurol. 2018;9:3. 29472886 10.3389/fneur.2018.00003 PMC5810419

[23] Miller J, Watrous AJ, Tsitsiklis M, et al Lateralised hippocampal oscillations underlie distinct aspects of human spatial memory and navigation. Nat Commun. 2018;9(1):2423. 29930307 10.1038/s41467-018-04847-9 PMC6013427

[24] Tyrer A, Gilbert JR, Adams S, et al Lateralised memory circuit dropout in Alzheimer’s disease patients. Brain Commun. 2020;2(2):fcaa212. 33409493 10.1093/braincomms/fcaa212 PMC7772115

[25] Lee J, Ha S, Kim REY, Lee M, Kim D, Lim HK. Development of amyloid PET analysis pipeline using deep learning-based brain MRI segmentation: A comparative validation study. Diagnostics (Basel). 2022;12(3):623. 35328176 10.3390/diagnostics12030623 PMC8947654

[26] Lee JH, Lee KU, Lee DY, et al Development of the Korean version of the consortium to establish a registry for Alzheimer’s disease assessment packet (CERAD-K): Clinical and neuropsychological assessment batteries. J Gerontol B Psychol Sci Soc Sci. 2002;57(1):P47–P53. 11773223 10.1093/geronb/57.1.p47

[27] Cummings JL, Mega M, Gray K, Rosenberg-Thompson S, Carusi DA, Gornbein J. The neuropsychiatric inventory: Comprehensive assessment of psychopathology in dementia. Neurology. 1994;44(12):2308–2314. 7991117 10.1212/wnl.44.12.2308

[28] Stoodley CJ, Schmahmann JD. Functional topography of the human cerebellum. In: Gruol DL, Koibuchi N, Manto M, Molinari M, Schmahmann JD, Shen Y, eds. Essentials of cerebellum and cerebellar disorders: A primer for graduate students. Springer International Publishing; 2016:373–381.

[29] Gellersen HM, Guell X, Sami S. Differential vulnerability of the cerebellum in healthy ageing and Alzheimer’s disease. Neuroimage Clin. 2021;30:102605. 33735787 10.1016/j.nicl.2021.102605 PMC7974323

[30] Adamaszek M, D’Agata F, Ferrucci R, et al Consensus paper: Cerebellum and emotion. Cerebellum. 2017;16(2):552–576. 27485952 10.1007/s12311-016-0815-8

[31] Yao Q, Tang F, Wang Y, et al Effect of cerebellum stimulation on cognitive recovery in patients with Alzheimer disease: A randomised clinical trial. Brain Stimul. 2022;15(4):910–920. 35700915 10.1016/j.brs.2022.06.004

[32] Samstag CL, Chapman NH, Gibbons LE, et al Neuropathological correlates of vulnerability and resilience in the cerebellum in Alzheimer’s disease. Alzheimers Dement. 2024;21(2):e14428. 39713867 10.1002/alz.14428 PMC11848203

[33] Yang C, Liu G, Chen X, Le W. Cerebellum in Alzheimer’s disease and other neurodegenerative diseases: An emerging research frontier. MedComm (2020). (2020). 2024;5(7):e638. 10.1002/mco2.638 PMC11245631 39006764

[34] Chen Y, Qi Y, Hu Y, et al Integrated cerebellar radiomic-network model for predicting mild cognitive impairment in Alzheimer’s disease. Alzheimers Dement. 2025;21(1):e14361. 39535490 10.1002/alz.14361 PMC11782160

[35] Veréb D, Mijalkov M, Canal-Garcia A, et al Age-related differences in the functional topography of the locus coeruleus and their implications for cognitive and affective functions. eLife. 2023;12:RP87188. 37650882 10.7554/eLife.87188 PMC10471162

[36] Jacobs HIL, Hopkins DA, Mayrhofer HC, et al The cerebellum in Alzheimer’s disease: Evaluating its role in cognitive decline. Brain. 2018;141(1):37–47. 29053771 10.1093/brain/awx194

[37] Tang Y, Cao M, Li Y, et al Altered structural covariance of locus coeruleus in individuals with significant memory concern and patients with mild cognitive impairment. Cereb Cortex. 2023;33(13):8523–8533. 37130822 10.1093/cercor/bhad137 PMC10321106

[38] Buckner RL, Krienen FM, Castellanos A, Diaz JC, Yeo BTT. The organisation of the human cerebellum estimated by intrinsic functional connectivity. J Neurophysiol. 2011;106(5):2322–2345. 21795627 10.1152/jn.00339.2011 PMC3214121

[39] Almeida J, Martins AR, Amaral L, et al The cerebellum is causally involved in episodic memory under ageing. Geroscience. 2023;45(4):2267–2287. 36749471 10.1007/s11357-023-00738-0 PMC10651631

[40] Starowicz-Filip A, Prochwicz K, Kłosowska J, et al Cerebellar functional lateralisation from the perspective of clinical neuropsychology. Front Psychol. 2021;12:775308. 34955995 10.3389/fpsyg.2021.775308 PMC8703197

[41] Zhang P, Duan L, Ou Y, et al The cerebellum and cognitive neural networks. Front Hum Neurosci. 2023;17:1197459. 37576472 10.3389/fnhum.2023.1197459 PMC10416251

[42] Bernard JA, Seidler RD, Hassevoort KM, et al Resting state cortico-cerebellar functional connectivity networks: A comparison of anatomical and self-organising map approaches. Front Neuroanat. 2012;6:31. 22907994 10.3389/fnana.2012.00031 PMC3415673

[43] Saadon-Grosman N, Du J, Kosakowski HL, et al Within-individual organization of the human cognitive cerebellum: Evidence for closely juxtaposed, functionally specialized regions. Sci Adv. 2024;10(45):eadq4037. 39514652 10.1126/sciadv.adq4037 PMC11546750

[44] Van Overwalle F, Ma Q, Heleven E. The posterior crus II cerebellum is specialised for social mentalising and emotional self-experiences: A meta-analysis. Soc Cogn Affect Neurosci. 2020;15(9):905–928. 32888303 10.1093/scan/nsaa124 PMC7851889

[45] Baillieux H, De Smet HJ, Paquier PF, De Deyn PP, Mariën P. Cerebellar neurocognition: Insights into the bottom of the brain. Clin Neurol Neurosurg. 2008;110(8):763–773. 18602745 10.1016/j.clineuro.2008.05.013

[46] Zhou J, Greicius MD, Gennatas ED, et al Divergent network connectivity changes in behavioural variant frontotemporal dementia and Alzheimer’s disease. Brain. 2010;133(5):1352–1367. 20410145 10.1093/brain/awq075 PMC2912696

[47] Braak H, Braak E. Neuropathological stageing of Alzheimer-related changes. Acta Neuropathol. 1991;82(4):239–259. 1759558 10.1007/BF00308809

[48] Brier MR, Thomas JB, Snyder AZ, et al Loss of intranetwork and internetwork resting state functional connections with Alzheimer’s disease progression. J Neurosci. 2012;32(26):8890–8899. 22745490 10.1523/JNEUROSCI.5698-11.2012 PMC3458508

[49] Mattsson-Carlgren N, Salvadó G, Ashton NJ, et al Prediction of longitudinal cognitive decline in preclinical Alzheimer disease using plasma biomarkers. JAMA Neurol. 2023;80(4):360–369. 36745413 10.1001/jamaneurol.2022.5272 PMC10087054

[50] Ng KP, Pascoal TA, Mathotaarachchi S, et al Neuropsychiatric symptoms predict hypometabolism in preclinical Alzheimer disease. Neurology. 2017;88(19):1814–1821. 28404803 10.1212/WNL.0000000000003916 PMC5419982

[51] Faber J, Kügler D, Bahrami E, et al CerebNet: A fast and reliable deep-learning pipeline for detailed cerebellum sub-segmentation. Neuroimage. 2022;264:119703. 36349595 10.1016/j.neuroimage.2022.119703 PMC9771831

