# Supplementary material for: Cerebellar subregional structural changes across the Alzheimer’s disease continuum: a longitudinal analysis of cognitive and behavioural correlates
Source: Brain Commun. 2025 Dec 23;8(1):fcaf500. doi: 10.1093/braincomms/fcaf500 (PMC12782110; doi:10.1093/braincomms/fcaf500)
Supplement: fcaf500_Supplementary_Data [file fcaf500_supplementary_data.docx]

**Supplementary Materials for “Cerebellar subregional structural changes across the Alzheimer’s disease continuum: a longitudinal analysis of cognitive and behavioral correlates” by Um et al.**

**Supplementary Methods**

S1. Diagnostic classification and exclusion criteria

S2. Apolipoprotein ε (APOE) genotyping

S3. Neuroimaging parameters and preprocessing steps

S4. Cognitive and neuropsychiatric assessments

S5. Statistical analysis and software details

**Supplementary Tables**

Supplementary Table S1 - Results of ANCOVA for regional cerebellar volumes across diagnostic groups (HC, PAD, MCI_AD, ADD), adjusted for covariates.

Supplementary Table S2 - Post-hoc pairwise comparisons for regional cerebellar volumes across diagnostic groups highlighting significant differences following ANCOVA.

Supplementary Table S3. Longitudinal changes in cerebellar total volume, grey matter, white matter volumes (mm^3^) over time in HC, PAD, MCI_AD, ADD groups, adjusted for covariates (age, sex, education, apoe4 carrier status and total intracranial volume) using GEE analysis

Supplementary Table S4 - Longitudinal changes in cerebellar regional volumes over time in HC Group, adjusted for covariates using GEE analysis.

Supplementary Table S5 - Longitudinal changes in cerebellar regional volumes over time in PAD Group, adjusted for covariates using GEE analysis.

Supplementary Table S6 - Longitudinal changes in cerebellar regional volumes over time in MCI_AD Group, adjusted for covariates using GEE analysis.

Supplementary Table S7 - Longitudinal changes in cerebellar regional volumes over time in ADD Group, adjusted for covariates using GEE analysis.

Supplementary Table S8- Partial correlation analysis between longitudinal changes in significant cerebellar regions and CERAD-K composite scores in the PAD group, adjusted for covariates.

Supplementary Table S9- Partial correlation analysis between longitudinal changes in significant cerebellar regions and CERAD-K composite scores in the ADD group, adjusted for covariates.

Supplementary Table S10 - Partial correlation analysis between longitudinal changes in significant cerebellar regions and NPI scores in the PAD group, adjusted for covariates.

Supplementary Table S11 - Partial correlation analysis between longitudinal changes in significant cerebellar regions and NPI scores in the ADD group, adjusted for covariates.

Supplementary Table S12 - Partial correlation analysis between longitudinal changes in significant cerebellar regions and cortical volume in the PAD group, adjusted for covariates.

Supplementary Table S13 - Partial correlation analysis between longitudinal changes in significant cerebellar regions and cortical volume in the ADD group, adjusted for covariates.

Supplementary Table S14. Association between baseline Aβ burden and longitudinal cerebellar volume change in the PAD group

Supplementary Table S15. Association between baseline Aβ burden and longitudinal cerebellar volume change in the MCI_AD group

Supplementary Table S16. Association between baseline Aβ burden and longitudinal cerebellar volume change in the ADD group

**Supplementary Data**

Supplementary Data 1. Analysis Codes (Python Scripts)

**Supplementary References**

**Supplementary Methods**

**S1. Diagnostic classification and exclusion criteria**

This longitudinal study included 229 older adults recruited from the Catholic Aging Brain Imaging Database (CABID). Participants were individuals who visited the Catholic Brain Health Center, Yeouido St. Mary’s Hospital, The Catholic University of Korea, between 2018 and 2023. Eligible participants were aged 60 years or older and underwent MRI, PET, and comprehensive clinical assessments, including the Korean Version of the Consortium to Establish a Registry for Alzheimer’s Disease Assessment Packet (CERAD-K)^1^and the Neuropsychiatric Inventory (NPI)^2^. The mean follow-up duration was two years.

Participants were classified into four diagnostic groups based on clinical evaluation and biomarker evidence, incorporating the National Institute on Aging and Alzheimer's Association (NIA-AA) diagnostic framework^3^, which provides distinct recommendations for the preclinical, mild cognitive impairment, and dementia stages of Alzheimer’s disease.:

1. Healthy control (HC): Participants with a clinical dementia rating (CDR)^4^ score of 0, no clinical evidence of cognitive impairment, and negative amyloid PET results indicating the absence of amyloid- Aβ deposition.

2.Preclinical Alzheimer’s disease (PAD): Participants with a CDR score of 0, no objective cognitive deficits on neuropsychological testing, but positive amyloid PET findings indicating biomarker evidence of Aβ deposition.

3. Mild cognitive impairment due to AD (MCI_AD): Defined according to Petersen’s criteria^5^, participants exhibited objective cognitive deficits on neuropsychological testing, with performance in at least one of the eight CERAD-K domains more than 1.5 standard deviations below age- and education-adjusted norms. They also had preserved general cognitive function, intact activities of daily living with a CDR score of 0.5, and positive amyloid PET findings indicative of Aβ deposition.

4. Alzheimer’s disease dementia (ADD): Diagnosed based on established clinical criteria and with a CDR score of 1 or more, participants exhibited significant cognitive and functional decline consistent with dementia, along with amyloid PET evidence of Aβ deposition.

Participants were excluded if they met any of the following criteria. Individuals with neurological disorders, including non-AD neurodegenerative diseases such as Parkinson’s disease, Lewy body dementia, or frontotemporal dementia, were not eligible. Additionally, those with a history of stroke, epilepsy, normal pressure hydrocephalus, or significant traumatic brain injury were excluded from the study. Participants with major psychiatric disorders were also ineligible, including those diagnosed with active major depressive disorder, bipolar disorder, schizophrenia, or other psychotic disorders as defined by Diagnostic and Statistical Manual of Mental Disorders, Fifth Edition. Furthermore, individuals with severe anxiety disorders or a history of substance use disorders within the past year were excluded. Exclusion criteria also encompassed medical conditions that could affect cognition, such as uncontrolled diabetes, thyroid dysfunction, renal failure, hepatic dysfunction, or chronic inflammatory diseases. Individuals with active malignancies, except for treated basal cell carcinoma, were also excluded. Finally, participants with contraindications to neuroimaging were not eligible for inclusion. This included individuals unable to undergo magnetic resonance imaging due to metal implants or severe claustrophobia, as well as those with contraindications to positron emission tomography imaging, such as an allergy to radiotracers.

**S2. Neuroimaging parameters, acquisition and preprocessing steps**

Structural T1-weighted MRI and amyloid PET scans were acquired for all participants. MRI imaging was conducted using a Siemens Skyra 3T scanner (Siemens Healthcare, Erlangen, Germany) equipped with a 20-channel head and neck coil. T1-weighted structural images were collected using a magnetization-prepared rapid gradient echo (MPRAGE) sequence with the following parameters: repetition time (TR) of 1860 ms, echo time (TE) of 25.3 ms, flip angle of 9°, field of view (FOV) measuring 224 × 224 mm, and a matrix size of 256 × 256. A total of 208 axial slices were acquired, each with a slice thickness of 1.0 mm. Amyloid PET imaging was performed using a Biograph 64 or Vision 600 scanner (Siemens Medical Solutions Inc., USA). Static PET scans were obtained 90–110 minutes after administering 185 MBq of flutemetamol, with a matrix size of 256 × 256 and a voxel size of 1.3364 × 1.3364 × 3 mm³. The resulting DICOM files were anonymized and converted to NIfTI format using the “dcm2niix” tool ^6^.

Amyloid PET images were analyzed to calculate the global amyloid standardized uptake value ratio (SUVR). The global amyloid SUVR was measured using automated segmentation software powered by a deep-learning model (SCALE PET v. 0.1.3.1, developed by Neurophet, South Korea)^7^. The use of the pons as a reference region for amyloid PET SUVR calculation is well-established in the literature^8^. A global and regional SUVR threshold of 0.62 was employed to classify amyloid positivity, consistent with established criteria in prior research^9^.

T1-weighted structural MRI images were processed and cortical volumes were calculated using the FreeSurfer image analysis suite (version 6.0, http://surfer.nmr.mgh.harvard.edu), following methods detailed in previously published studies^10,11^. The regions of interest (ROIs) selected for this study—frontal lobe, temporal lobe, posterior cingulate cortex (PCC), hippocampus, and insula^12-14^. For each ROI, both the left hemisphere (denoted as "lh") and the right hemisphere (denoted as "rh") structures were analyzed to capture lateralized effects and ensure comprehensive coverage of the brain regions ^15-17^. Cortical volume analysis was conducted across a range of regions of interest (ROIs) covering the frontal, temporal, cingulate, hippocampal, and insular cortices. Specifically, the frontal lobe ROIs included the lateral orbitofrontal gyrus, medial orbitofrontal gyrus, rostral middle frontal gyrus, caudal middle frontal gyrus, and superior frontal gyrus, each analyzed bilaterally. Temporal lobe ROIs encompassed the inferior, middle, and superior temporal gyri, as well as the transverse temporal gyrus, also in both hemispheres. The posterior cingulate cortex (PCC) network was represented by the caudal anterior cingulate cortex, rostral anterior cingulate cortex, isthmus cingulate cortex, and the posterior cingulate cortex itself, all examined in left and right hemispheres. In addition, the parahippocampal gyrus (hippocampal region) and the insular cortex were included as ROIs, bilaterally. These ROIs were extracted using the FreeSurfer image analysis suite (version 6.0).

Normalized cerebellar volumes were extracted and segmented using Statistical Parametric Mapping version 12 (SPM12; Wellcome Department of Cognitive Neurology, London, UK) with the Computational Anatomy Toolbox version 12 (CAT12; C. Gaser, Structural Brain Mapping Group, Jena University Hospital, Germany)^18^, and cerebellar parcellation via Spatially Unbiased Infratentorial Template (SUIT)^19^, with total intracranial volume (TIV) corrected. The cerebellar regions analyzed in this study, as defined by the SUIT atlas, encompass 28 distinct areas that provide a comprehensive representation of cerebellar anatomy. These regions include left I-IV, right I-IV, left V, right V, left VI, vermis VI, right VI, left Crus I, vermis Crus I, right Crus I, left Crus II, vermis Crus II, right Crus II, left VIIb, vermis VIIb, right VIIb, left VIIIa, vermis VIIIa, right VIIIa, left VIIIb, vermis VIIIb, right VIIIb, left IX, vermis IX, right IX, left X, vermis X, and right X. The CAT12 processing pipeline incorporates an optimized voxel-based morphometry (VBM) framework, which includes preprocessing steps such as tissue segmentation, bias field correction, and nonlinear spatial normalization using DARTEL. T1-weighted structural MRI images were initially segmented into gray matter (GM), white matter (WM), and cerebrospinal fluid (CSF) using CAT12’s segmentation algorithm. The SUIT toolbox was then applied to extract the cerebellum separately from supratentorial regions, ensuring accurate isolation of cerebellar structures. The cerebellar images were registered to the SUIT template, a dedicated high-resolution cerebellar atlas designed to enhance anatomical localization and reduce individual variability. This step involved a nonlinear registration process that preserved the distinct morphology of the cerebellum while aligning it to a standardized space. To maintain accurate volumetric information following normalization, images were modulated by multiplying with the Jacobian determinant derived from the spatial transformation. This adjustment ensured that the extracted cerebellar volumes reflected true anatomical differences rather than deformations introduced by normalization. Normalized and modulated images were used to compute cerebellar regional volumes based on predefined SUIT parcellations. This allowed for lobule-specific analyses while ensuring spatial transformations did not distort volumetric estimates. Total intracranial volume was automatically estimated in CAT12 and was included as a covariate in statistical models where necessary to account for differences in overall brain size.

**S3. Cognitive and neuropsychiatric assessments**

Originally developed for the standardized clinical and neuropsychological assessment of Alzheimer’s disease, the CERAD-K^1^ has been validated and proven reliable for use in the Korean population. The CERAD-K battery included tests such as Verbal Fluency (VF), the 15-item Boston Naming Test (BNT), Mini-Mental State Examination-Korean version (MMSE-K), Word List Memory (WLM), Word List Recall (WLR), Word List Recognition (WLRc), Constructional Praxis (CP), and Constructional Recall (CR). A neuropsychologist reviewed the results to determine the presence of cognitive impairment. Each test was scored according to established criteria: VF measured the number of animal names generated in one minute, while BNT had a maximum score of 15 points. The MMSE-K ranged from 0 to 30 points, WLM from 0 to 30 points, WLR and WLRc from 0 to 10 points each, and CP and CR from 0 to 11 points each.

Episodic memory composite score was defined as the combined score of word list memory, word list recall, word list recognition, and constructional praxis recall ^20^. Non-episodic memory composite score was defined as the composite score of verbal fluency, the modified Boston Naming Test, Mini-mental status examination (MMSE), and constructional praxis. ^20^

The NPI assesses 12 behavioral domains: delusions, hallucinations, agitation/aggression, depression, anxiety, elation/euphoria, apathy/indifference, disinhibition, irritability/lability, aberrant motor behavior, sleep disturbances, and appetite/eating changes^2^. While the NPI traditionally includes 12 domains, recent studies have grouped these into four broader domains based on factor analyses to better categorize behavioral and psychological symptoms. The Neuropsychiatric Inventory (NPI) components were grouped into four main categories based on symptom domains: mood symptoms (npi_mood: Depression/Dysphoria and Anxiety), psychotic symptoms (npi_psycho: Delusions and Hallucinations), hyperactivity symptoms (npi_hyper: Agitation/Aggression, Disinhibition, and Irritability/Lability), and behavioral symptoms (npi_beha: Apathy/Indifference, Aberrant Motor Behavior, Night-time Behavior Disturbances, and Appetite/Eating Disturbances) ^21^.

**S4. Statistical analysis and software details**

A one-way analysis of variance (ANOVA) was conducted to compare demographic, neuroimaging, and cognitive variables across four diagnostic groups: HC, PAD, MCI_AD and ADD. The assumptions of normality and homogeneity of variance were tested using the Shapiro-Wilk test and Levene’s test, respectively. Post-hoc pairwise comparisons were performed using Tukey’s Honest Significant Difference (HSD) test for normally distributed data and Games-Howell correction where variances were unequal. Categorical variables, such as sex and apolipoprotein ε4 genotype(APOE4) carrier status, were analyzed using chi-square tests. For each cerebellar region, mean ± standard deviation (SD) of volumetric values were calculated within each diagnostic group. Analysis of covariance (ANCOVA) was performed to evaluate differences in regional volumes across the four diagnostic groups (HC, PAD, MCI_AD, ADD). Age, sex, APOE4, TIV, and education were included as covariates to control for potential confounding effects. Post-hoc pairwise comparisons were conducted using Tukey's HSD test to identify specific group differences. Longitudinal volumetric changes in cerebellar regions, total cerebellar volume, grey matter volume, white matter volume were further analyzed using GEE to account for repeated measures and time-dependent variations. Significant regions identified from ANCOVA were subjected to GEE modeling, with time as the independent variable and the same set of covariates for adjustment. Results were reported as coefficients (β) with 95% confidence intervals and p-values. Partial correlation analyses were conducted to examine the relationship between cerebellar volume changes and cognitive/neuropsychiatric measures. Specifically, only diagnostic groups in which significant longitudinal cerebellar volume reductions were identified through GEE were included in the analysis. Within these groups, partial correlations were performed between the volumetric changes in cerebellar regions that showed significant reductions and changes in composite scores of episodic and non-episodic memory from the CERAD-K, as well as changes in composite NPI scores and cortical volumes. Analyses controlled for covariates including age, sex, APOE4 status, TIV, and education. Results were presented as Pearson’s partial correlation coefficients (r) with corresponding p-values. Statistical significance for all analyses was set at p < 0.05. All statistical analyses were conducted using Python (version 3.9.7), employing a range of specialized libraries to ensure rigorous and reproducible results. Data manipulation and preprocessing were performed using pandas (version 1.3.3), which facilitated efficient handling of large datasets. For statistical modeling, including ANCOVA and partial correlation analyses, statsmodels (version 0.13.1) was utilized. Visualization of data distributions and statistical relationships was achieved through seaborn (version 0.11.2), while matplotlib (version 3.4.3) was employed to generate high-quality figures suitable for publication. Numerical computations and array-based operations were supported by numpy (version 1.21.2), and scipy (version 1.7.1) was used to perform various statistical tests, including correlation analyses. Finally, sklearn (version 0.24.2) was used to preprocess and standardize covariates, ensuring accuracy in partial correlation analyses.

**S5. Apolipoprotein ε (APOE) genotyping**

To determine APOE genotype, DNA was extracted from participants’ blood samples using the QIAamp Blood DNA Maxi Kit (Qiagen, Valencia, CA, USA). TaqMan SNP genotyping assays (Applied Biosystems, Foster City, CA, USA) were used to identify two key APOE single nucleotide polymorphisms (SNPs): rs429358 (E4) and rs7412 (E2). Participants were classified as APOE ε4 carriers if they possessed at least one ε4 allele, whereas those without the ε4 allele were designated as APOE ε4 non-carriers.

**Supplementary Tables**

**Supplementary Table S1. Results of ANCOVA for baseline regional cerebellar volumes (mm^3^±standard deviation) across diagnostic groups (HC, PAD, MCI_AD, ADD), adjusted for age, sex, education, APOE4 carrier status and TIV**

|  | **HC** | **PAD** | **MCI_AD** | **ADD** | **P value** |
| --- | --- | --- | --- | --- | --- |
| **Left I-IV** | 1.83 ± 0.21 | 1.84 ± 0.22 | 1.82 ± 0.24 | 1.77 ± 0.20 | 0.815 |
| **Right I-IV** | 2.19 ± 0.26 | 2.21 ± 0.25 | 2.19 ± 0.28 | 2.11 ± 0.23 | 0.437 |
| **Left V** | 2.64 ± 0.32 | 2.67 ± 0.28 | 2.64 ± 0.31 | 2.53 ± 0.32 | 0.324 |
| **Right V** | 2.79 ± 0.37 | 2.84 ± 0.31 | 2.75 ± 0.34 | 2.64 ± 0.34 | 0.255 |
| **Left VI** | 5.94 ± 0.87 | 5.94 ± 0.69 | 5.80 ± 0.65 | 5.46 ± 0.82 | **0.023** |
| **Vermis VI** | 1.16 ± 0.16 | 1.15 ± 0.13 | 1.13 ± 0.13 | 1.09 ± 0.12 | 0.384 |
| **Right VI** | 5.60 ± 0.88 | 5.67 ± 0.71 | 5.48 ± 0.67 | 5.23 ± 0.83 | 0.149 |
| **Left Crus I** | 8.05 ± 1.11 | 8.25 ± 1.08 | 7.80 ± 0.96 | 7.60 ± 1.11 | 0.132 |
| **Vermis Crus I** | 0.01 ± 0.00 | 0.01 ± 0.00 | 0.01 ± 0.00 | 0.01 ± 0.00 | 0.119 |
| **Right Crus I** | 7.78 ± 1.20 | 8.09 ± 1.15 | 7.71 ± 0.97 | 7.54 ± 0.95 | 0.302 |
| **Left Crus II** | 6.39 ± 0.85 | 6.43 ± 0.72 | 6.08 ± 0.86 | 5.69 ± 0.91 | **<0.001** |
| **Vermis Crus II** | 0.29 ± 0.04 | 0.29 ± 0.03 | 0.28 ± 0.04 | 0.28 ± 0.04 | 0.502 |
| **Right Crus II** | 6.32 ± 0.91 | 6.42 ± 0.79 | 5.99 ± 0.91 | 5.66 ± 0.98 | **0.001** |
| **Left VIIb** | 3.58 ± 0.51 | 3.60 ± 0.46 | 3.43 ± 0.49 | 3.24 ± 0.52 | **0.005** |
| **Vermis VIIb** | 0.15 ± 0.02 | 0.14 ± 0.02 | 0.14 ± 0.02 | 0.13 ± 0.02 | 0.094 |
| **Right VIIb** | 3.50 ± 0.51 | 3.52 ± 0.44 | 3.33 ± 0.50 | 3.12 ± 0.59 | **0.001** |
| **Left VIIIa** | 3.42 ± 0.48 | 3.42 ± 0.39 | 3.26 ± 0.48 | 3.12 ± 0.51 | **0.013** |
| **Vermis VIIIa** | 0.66 ± 0.09 | 0.67 ± 0.08 | 0.64 ± 0.10 | 0.61 ± 0.09 | **0.025** |
| **Right VIIIa** | 3.29 ± 0.47 | 3.30 ± 0.40 | 3.13 ± 0.48 | 2.97 ± 0.54 | **0.003** |
| **Left VIIIb** | 2.49 ± 0.33 | 2.54 ± 0.31 | 2.38 ± 0.38 | 2.27 ± 0.36 | **0.010** |
| **Vermis VIIIb** | 0.35 ± 0.05 | 0.36 ± 0.04 | 0.33 ± 0.05 | 0.33 ± 0.06 | **0.017** |
| **Right VIIIb** | 2.56 ± 0.36 | 2.55 ± 0.35 | 2.44 ± 0.38 | 2.31 ± 0.32 | **0.008** |
| **Left IX** | 1.59 ± 0.29 | 1.73 ± 0.27 | 1.59 ± 0.29 | 1.46 ± 0.26 | **<0.001** |
| **Vermis IX** | 0.43 ± 0.07 | 0.46 ± 0.06 | 0.42 ± 0.07 | 0.41 ± 0.07 | **0.029** |
| **Right IX** | 1.98 ± 0.31 | 2.04 ± 0.30 | 1.91 ± 0.33 | 1.82 ± 0.30 | **0.020** |
| **Left X** | 0.33 ± 0.05 | 0.35 ± 0.05 | 0.32 ± 0.04 | 0.32 ± 0.04 | **0.006** |
| **Vermis X** | 0.20 ± 0.03 | 0.20 ± 0.03 | 0.20 ± 0.03 | 0.19 ± 0.03 | 0.588 |
| **Right X** | 0.35 ± 0.04 | 0.37 ± 0.05 | 0.34 ± 0.04 | 0.33 ± 0.04 | **0.014** |

Group differences were tested using ANCOVA adjusted for age, sex, years of education, TIV, APOE4 carrier status. Error bars represent standard errors of the mean.

Abbreviations: ANCOVA, analysis of covariance, HC, healthy control; PAD, preclinical Alzheimer’s disease; MCI_AD, mild cognitive impairment due to Alzheimer’s disease; ADD, Alzheimer’s disease dementia; TIV, total intracranial volume, APOE4, apolipoprotein ε4

**Supplementary Table S2. Post-hoc pairwise comparisons for regional cerebellar volumes(mm^3^) across diagnostic groups (HC, PAD, MCI_AD, ADD) showing significant differences following ANCOVA**

| **Region** | **Group1** | **Group2** | **Mean Diff** | **p-adj** |
| --- | --- | --- | --- | --- |
| **Left VI** | **ADD** | **HC** | 0.484 | **0.006** |
| **Left VI** | **ADD** | **MCI_AD** | 0.34 | 0.062 |
| **Left VI** | **ADD** | **PAD** | 0.482 | **0.008** |
| **Left VI** | **HC** | **MCI_AD** | -0.144 | 0.637 |
| **Left VI** | **HC** | **PAD** | -0.002 | 1.000 |
| **Left VI** | **MCI_AD** | **PAD** | 0.142 | 0.666 |
| **Left Crus II** | **ADD** | **HC** | 0.698 | **<0.001** |
| **Left Crus II** | **ADD** | **MCI_AD** | 0.395 | **0.498** |
| **Left Crus II** | **ADD** | **PAD** | 0.739 | **<0.001** |
| **Left Crus II** | **HC** | **MCI_AD** | -0.303 | 0.122 |
| **Left Crus II** | **HC** | **PAD** | 0.041 | 0.993 |
| **Left Crus II** | **MCI_AD** | **PAD** | 0.344 | 0.071 |
| **Right Crus II** | **ADD** | **HC** | 0.666 | **0.001** |
| **Right Crus II** | **ADD** | **MCI_AD** | 0.331 | 0.184 |
| **Right Crus II** | **ADD** | **PAD** | 0.761 | **<0.001** |
| **Right Crus II** | **HC** | **MCI_AD** | -0.336 | 0.104 |
| **Right Crus II** | **HC** | **PAD** | 0.095 | 0.939 |
| **Right Crus II** | **MCI_AD** | **PAD** | 0.431 | **0.024** |
| **Left VIIb** | **ADD** | **HC** | 0.347 | **0.003** |
| **Left VIIb** | **ADD** | **MCI_AD** | 0.191 | 0.154 |
| **Left VIIb** | **ADD** | **PAD** | 0.366 | **0.002** |
| **Left VIIb** | **HC** | **MCI_AD** | -0.157 | 0.217 |
| **Left VIIb** | **HC** | **PAD** | 0.018 | 0.997 |
| **Left VIIb** | **MCI_AD** | **PAD** | 0.175 | 0.154 |
| **Right VIIb** | **ADD** | **HC** | 0.378 | **0.001** |
| **Right VIIb** | **ADD** | **MCI_AD** | 0.21 | 0.109 |
| **Right VIIb** | **ADD** | **PAD** | 0.404 | **0.001** |
| **Right VIIb** | **HC** | **MCI_AD** | -0.168 | 0.184 |
| **Right VIIb** | **HC** | **PAD** | 0.026 | 0.992 |
| **Right VIIb** | **MCI_AD** | **PAD** | 0.194 | 0.107 |
| **Left VIIIa** | **ADD** | **HC** | 0.298 | **0.007** |
| **Left VIIIa** | **ADD** | **MCI_AD** | 0.138 | 0.367 |
| **Left VIIIa** | **ADD** | **PAD** | 0.299 | **0.009** |
| **Left VIIIa** | **HC** | **MCI_AD** | -0.16 | 0.158 |
| **Left VIIIa** | **HC** | **PAD** | 0.001 | 1.000 |
| **Left VIIIa** | **MCI_AD** | **PAD** | 0.161 | 0.173 |
| **Vermis VIIIa** | **ADD** | **HC** | 0.051 | **0.028** |
| **Vermis VIIIa** | **ADD** | **MCI_AD** | 0.028 | 0.337 |
| **Vermis VIIIa** | **ADD** | **PAD** | 0.062 | **0.005** |
| **Vermis VIIIa** | **HC** | **MCI_AD** | -0.022 | 0.435 |
| **Vermis VIIIa** | **HC** | **PAD** | 0.012 | 0.897 |
| **Vermis VIIIa** | **MCI_AD** | **PAD** | 0.034 | 0.117 |
| **Right VIIIa** | **ADD** | **HC** | 0.319 | **0.004** |
| **Right VIIIa** | **ADD** | **MCI_AD** | 0.165 | 0.227 |
| **Right VIIIa** | **ADD** | **PAD** | 0.332 | **0.00**3 |
| **Right VIIIa** | **HC** | **MCI_AD** | -0.154 | 0.197 |
| **Right VIIIa** | **HC** | **PAD** | 0.013 | 0.999 |
| **Right VIIIa** | **MCI_AD** | **PAD** | 0.167 | 0.157 |
| **Left VIIIb** | **ADD** | **HC** | 0.217 | **0.010** |
| **Left VIIIb** | **ADD** | **MCI_AD** | 0.109 | 0.323 |
| **Left VIIIb** | **ADD** | **PAD** | 0.261 | **0.001** |
| **Left VIIIb** | **HC** | **MCI_AD** | -0.108 | 0.234 |
| **Left VIIIb** | **HC** | **PAD** | 0.044 | 0.900 |
| **Left VIIIb** | **MCI_AD** | **PAD** | 0.152 | **0.048** |
| **Vermis VIIIb** | **ADD** | **HC** | 0.025 | 0.058 |
| **Vermis VIIIb** | **ADD** | **MCI_AD** | 0.005 | 0.951 |
| **Vermis VIIIb** | **ADD** | **PAD** | 0.031 | **0.014** |
| **Vermis VIIIb** | **HC** | **MCI_AD** | -0.02 | 0.069 |
| **Vermis VIIIb** | **HC** | **PAD** | 0.005 | 0.935 |
| **Vermis VIIIb** | **MCI_AD** | **PAD** | 0.026 | **0.014** |
| **Right VIIIb** | **ADD** | **HC** | 0.245 | **0.003** |
| **Right VIIIb** | **ADD** | **MCI_AD** | 0.125 | 0.224 |
| **Right VIIIb** | **ADD** | **PAD** | 0.239 | **0.005** |
| **Right VIIIb** | **HC** | **MCI_AD** | -0.12 | 0.173 |
| **Right VIIIb** | **HC** | **PAD** | -0.006 | 1.000 |
| **Right VIIIb** | **MCI_AD** | **PAD** | 0.114 | 0.229 |
| **Left IX** | **ADD** | **HC** | 0.131 | 0.084 |
| **Left IX** | **ADD** | **MCI_AD** | 0.134 | **0.045** |
| **Left IX** | **ADD** | **PAD** | 0.268 | **<0.001** |
| **Left IX** | **HC** | **MCI_AD** | 0.003 | 1.000 |
| **Left IX** | **HC** | **PAD** | 0.137 | **0.039** |
| **Left IX** | **MCI_AD** | **PAD** | 0.134 | **0.023** |
| **Vermis IX** | **ADD** | **HC** | 0.023 | 0.331 |
| **Vermis IX** | **ADD** | **MCI_AD** | 0.006 | 0.964 |
| **Vermis IX** | **ADD** | **PAD** | 0.044 | 0.009 |
| **Vermis IX** | **HC** | **MCI_AD** | -0.017 | 0.434 |
| **Vermis IX** | **HC** | **PAD** | 0.021 | 0.351 |
| **Vermis IX** | **MCI_AD** | **PAD** | 0.038 | **0.006** |
| **Right IX** | **ADD** | **HC** | 0.154 | 0.064 |
| **Right IX** | **ADD** | **MCI_AD** | 0.086 | 0.433 |
| **Right IX** | **ADD** | **PAD** | 0.22 | **0.003** |
| **Right IX** | **HC** | **MCI_AD** | -0.067 | 0.553 |
| **Right IX** | **HC** | **PAD** | 0.066 | 0.660 |
| **Right IX** | **MCI_AD** | **PAD** | 0.134 | 0.056 |
| **Left X** | **ADD** | **HC** | 0.019 | 0.145 |
| **Left X** | **ADD** | **MCI_AD** | 0.007 | 0.809 |
| **Left X** | **ADD** | **PAD** | 0.033 | **0.001** |
| **Left X** | **HC** | **MCI_AD** | -0.011 | 0.391 |
| **Left X** | **HC** | **PAD** | 0.015 | 0.286 |
| **Left X** | **MCI_AD** | **PAD** | 0.026 | **0.003** |
| **Right X** | **ADD** | **HC** | 0.021 | 0.073 |
| **Right X** | **ADD** | **MCI_AD** | 0.008 | 0.776 |
| **Right X** | **ADD** | **PAD** | 0.032 | **0.003** |
| **Right X** | **HC** | **MCI_AD** | -0.014 | 0.241 |
| **Right X** | **HC** | **PAD** | 0.01 | 0.593 |
| **Right X** | **MCI_AD** | **PAD** | 0.024 | **0.008** |

ANCOVA was performed to compare baseline regional cerebellar volumes across four diagnostic groups, adjusting for age, sex, years of education, APOE4 carrier status, and TIV. Post-hoc pairwise comparisons were conducted using Tukey’s HSD test. Significant group differences (p < 0.05) are highlighted in bold.

Abbreviations: ANCOVA, analysis of covariance; HC, healthy control; PAD, preclinical Alzheimer’s disease; MCI_AD, mild cognitive impairment due to Alzheimer’s disease; ADD, Alzheimer’s disease dementia; APOE4, apolipoprotein ε4; TIV, total intracranial volume; HSD, Honest Significant Difference

**Supplementary Table S3. Longitudinal changes in cerebellar total volume, grey matter, white matter volumes(mm^3^) over time in HC, PAD, MCI_AD, ADD groups, adjusted for covariates (age, sex, education, APOE4 carrier status and TIV) using GEE analysis**

| **Group** | **Measure** | ***β*** | **P value** | **CI Lower** | **CI Upper** |
| --- | --- | --- | --- | --- | --- |
| **HC** | **g_cerebellum** | 0.360 | 0.062 | -0.017 | 0.736 |
| **HC** | **w_cerebellum** | 0.090 | 0.438 | -0.139 | 0.319 |
| **HC** | **t_cerebellum** | 0.454 | **0.005** | 0.136 | 0.772 |
| **MCI_AD** | **g_cerebellum** | -0.226 | 0.154 | -0.537 | 0.085 |
| **MCI_AD** | **w_cerebellum** | 0.014 | 0.873 | -0.163 | 0.191 |
| **MCI_AD** | **t_cerebellum** | -0.224 | 0.106 | -0.496 | 0.048 |
| **PAD** | **g_cerebellum** | -0.425 | **0.041** | -0.831 | -0.018 |
| **PAD** | **w_cerebellum** | 0.247 | 0.080 | -0.030 | 0.523 |
| **PAD** | **t_cerebellum** | 0.045 | 0.912 | -0.748 | 0.837 |
| **ADD** | **g_cerebellum** | -0.737 | **0.018** | -1.349 | -0.125 |
| **ADD** | **w_cerebellum** | 0.195 | 0.406 | -0.265 | 0.654 |
| **ADD** | **t_cerebellum** | -0.600 | **0.015** | -1.082 | -0.117 |

GEE were used to examine longitudinal changes in total cerebellar, grey matter, and white matter volumes across four diagnostic groups (HC, PAD, MCI_AD, and ADD). Each model was adjusted for age, sex, years of education, APOE4 carrier status, and TIV. Regression coefficients (β), standard errors, and significance values (p < 0.05) are presented for time effects and group-by-time interactions.

Abbreviations: GEE, generalized estimating equation; HC, healthy control; PAD, preclinical Alzheimer’s disease; MCI_AD, mild cognitive impairment due to Alzheimer’s disease; ADD, Alzheimer’s disease dementia; APOE4, apolipoprotein ε4; TIV, total intracranial volume

**Supplementary Table S4. Longitudinal changes in cerebellar regional volumes(mm^3^) over time in HC Group, adjusted for covariates (age, sex, education, APOE4 carrier status and TIV) using GEE analysis**

| **Region** | ***β*** | **P value** | **CI Lower** | **CI Upper** |
| --- | --- | --- | --- | --- |
| **Left I-IV** | 0.013 | 0.346 | -0.014 | 0.039 |
| **Right I-IV** | 0.012 | 0.520 | -0.024 | 0.047 |
| **Left V** | 0.023 | 0.324 | -0.022 | 0.068 |
| **Right V** | 0.011 | 0.653 | -0.037 | 0.059 |
| **Left VI** | 0.030 | 0.615 | -0.088 | 0.149 |
| **Vermis VI** | -0.003 | 0.784 | -0.021 | 0.016 |
| **Right VI** | 0.045 | 0.435 | -0.067 | 0.156 |
| **Left Crus I** | 0.009 | 0.883 | -0.106 | 0.123 |
| **Vermis Crus I** | 0.000 | 0.192 | 0.000 | 0.001 |
| **Right Crus I** | 0.031 | 0.652 | -0.102 | 0.163 |
| **Left Crus II** | 0.014 | 0.799 | -0.097 | 0.126 |
| **Vermis Crus II** | 0.000 | 0.875 | -0.006 | 0.006 |
| **Right Crus II** | 0.016 | 0.740 | -0.076 | 0.107 |
| **Left VIIb** | 0.031 | 0.392 | -0.040 | 0.101 |
| **Vermis VIIb** | 0.000 | 0.795 | -0.003 | 0.004 |
| **Right VIIb** | 0.043 | 0.213 | -0.025 | 0.111 |
| **Left VIIIa** | 0.045 | 0.077 | -0.005 | 0.095 |
| **Vermis VIIIa** | 0.010 | 0.148 | -0.004 | 0.023 |
| **Right VIIIa** | 0.042 | 0.142 | -0.014 | 0.097 |
| **Left VIIIb** | 0.022 | 0.279 | -0.018 | 0.062 |
| **Vermis VIIIb** | 0.003 | 0.446 | -0.004 | 0.010 |
| **Right VIIIb** | 0.034 | 0.127 | -0.010 | 0.079 |
| **Left IX** | 0.005 | 0.783 | -0.029 | 0.039 |
| **Vermis IX** | 0.007 | 0.173 | -0.003 | 0.016 |
| **Right IX** | 0.024 | 0.263 | -0.018 | 0.067 |
| **Left X** | 0.001 | 0.839 | -0.005 | 0.006 |
| **Vermis X** | 0.003 | 0.188 | -0.001 | 0.006 |
| **Right X** | -0.004 | 0.232 | -0.010 | 0.002 |

GEE models were applied to evaluate longitudinal changes in cerebellar regional volumes within HC group. Each model was adjusted for age, sex, years of education, APOE4 carrier status, and TIV. Regression coefficients (β), standard errors, and significance values (p < 0.05) are presented for time effects.

Abbreviations: GEE, generalized estimating equation; HC, healthy control; APOE4, apolipoprotein ε4; TIV, total intracranial volume

**Supplementary Table S5. Longitudinal changes in cerebellar regional volumes over time in PAD Group, adjusted for covariates (age, sex, education, APOE4 carrier status and TIV) using GEE analysis**

| **Region** | ***β*** | **P value** | **CI Lower** | **CI Upper** |
| --- | --- | --- | --- | --- |
| **Left I-IV** | -0.012 | 0.084 | -0.026 | 0.002 |
| **Right I-IV** | -0.004 | 0.601 | -0.017 | 0.010 |
| **Left V** | -0.009 | 0.245 | -0.024 | 0.006 |
| **Right V** | -0.011 | 0.141 | -0.026 | 0.004 |
| **Left VI** | -0.048 | **0.012** | -0.085 | -0.010 |
| **Vermis VI** | -0.006 | 0.109 | -0.014 | 0.001 |
| **Right VI** | -0.044 | **0.005** | -0.075 | -0.013 |
| **Left Crus I** | -0.076 | **0.031** | -0.145 | -0.007 |
| **Vermis Crus I** | 0.000 | 0.232 | -0.001 | 0.000 |
| **Right Crus I** | -0.036 | 0.142 | -0.084 | 0.012 |
| **Left Crus II** | -0.071 | **0.033** | -0.136 | -0.006 |
| **Vermis Crus II** | -0.004 | 0.059 | -0.008 | 0.000 |
| **Right Crus II** | -0.061 | **0.013** | -0.109 | -0.013 |
| **Left VIIb** | -0.03 | 0.081 | -0.064 | 0.004 |
| **Vermis VIIb** | 0.000 | 0.840 | -0.002 | 0.001 |
| **Right VIIb** | -0.027 | 0.074 | -0.056 | 0.003 |
| **Left VIIIa** | -0.009 | 0.585 | -0.040 | 0.023 |
| **Vermis VIIIa** | -0.007 | 0.057 | -0.014 | 0.000 |
| **Right VIIIa** | -0.015 | 0.273 | -0.043 | 0.012 |
| **Left VIIIb** | -0.005 | 0.677 | -0.028 | 0.018 |
| **Vermis VIIIb** | 0.001 | 0.819 | -0.004 | 0.005 |
| **Right VIIIb** | 0.006 | 0.600 | -0.016 | 0.028 |
| **Left IX** | -0.004 | 0.587 | -0.020 | 0.011 |
| **Vermis IX** | -0.001 | 0.685 | -0.006 | 0.004 |
| **Right IX** | 0.002 | 0.840 | -0.019 | 0.023 |
| **Left X** | -0.001 | 0.408 | -0.004 | 0.002 |
| **Vermis X** | 0.000 | 0.791 | -0.002 | 0.002 |
| **Right X** | -0.003 | 0.085 | -0.006 | 0.000 |

# GEE models were used to assess longitudinal changes in cerebellar regional volumes within PAD group. Each model was adjusted for age, sex, years of education, APOE4 carrier status, and TIV. Regression coefficients (β), standard errors, and significance values (p < 0.05) are presented for time effects.

# Abbreviations: GEE, generalized estimating equation; PAD, preclinical Alzheimer’s disease; APOE4, apolipoprotein ε4; TIV, total intracranial volume

**Supplementary Table S6. Longitudinal changes in cerebellar regional volumes over time in MCI_AD Group, adjusted for covariates (age, sex, education, APOE4 carrier status and TIV) using GEE analysis**

| **Region** | ***β*** | **P value** | **CI Lower** | **CI Upper** |
| --- | --- | --- | --- | --- |
| **Left I-IV** | 0.008 | 0.153 | -0.003 | 0.018 |
| **Right I-IV** | 0.008 | 0.258 | -0.006 | 0.023 |
| **Left V** | 0.003 | 0.665 | -0.009 | 0.014 |
| **Right V** | 0.005 | 0.520 | -0.010 | 0.020 |
| **Left VI** | -0.006 | 0.694 | -0.034 | 0.022 |
| **Vermis VI** | -0.004 | 0.179 | -0.009 | 0.002 |
| **Right VI** | 0.003 | 0.825 | -0.025 | 0.031 |
| **Left Crus I** | -0.008 | 0.795 | -0.069 | 0.053 |
| **Vermis Crus I** | 0.000 | 0.408 | 0.000 | 0.000 |
| **Right Crus I** | -0.029 | 0.265 | -0.081 | 0.022 |
| **Left Crus II** | -0.022 | 0.343 | -0.066 | 0.023 |
| **Vermis Crus II** | -0.001 | 0.430 | -0.005 | 0.002 |
| **Right Crus II** | -0.007 | 0.816 | -0.062 | 0.049 |
| **Left VIIb** | 0.009 | 0.469 | -0.016 | 0.035 |
| **Vermis VIIb** | 0.000 | 0.980 | -0.002 | 0.002 |
| **Right VIIb** | 0.017 | 0.253 | -0.012 | 0.047 |
| **Left VIIIa** | 0.020 | 0.129 | -0.006 | 0.045 |
| **Vermis VIIIa** | -0.001 | 0.821 | -0.007 | 0.005 |
| **Right VIIIa** | 0.017 | 0.199 | -0.009 | 0.042 |
| **Left VIIIb** | 0.002 | 0.813 | -0.018 | 0.023 |
| **Vermis VIIIb** | 0.003 | 0.057 | 0.000 | 0.006 |
| **Right VIIIb** | 0.024 | 0.094 | -0.004 | 0.051 |
| **Left IX** | 0.000 | 0.979 | -0.016 | 0.017 |
| **Vermis IX** | 0.004 | 0.096 | -0.001 | 0.008 |
| **Right IX** | 0.009 | 0.392 | -0.012 | 0.030 |
| **Left X** | 0.000 | 0.796 | -0.002 | 0.002 |
| **Vermis X** | 0.002 | 0.075 | 0.000 | 0.004 |
| **Right X** | 0.002 | 0.263 | -0.001 | 0.005 |

GEE models were employed to evaluate longitudinal changes in cerebellar regional volumes within MCI_AD group. Each model was adjusted for age, sex, years of education, APOE4 carrier status, TIV. Regression coefficients (β), standard errors, and significance values (p < 0.05) are presented for time effects.

Abbreviations: GEE, generalized estimating equation; MCI_AD, mild cognitive impairment due to Alzheimer’s disease; APOE4, apolipoprotein ε4; TIV, total intracranial volume

**Supplementary Table S7.** **Longitudinal changes in cerebellar regional volumes over time in ADD Group, adjusted for covariates (age, sex, education, APOE4 carrier status and TIV) using GEE analysis**

| **Region** | ***β*** | **P value** | **CI Lower** | **CI Upper** |
| --- | --- | --- | --- | --- |
| **Left I-IV** | -0.009 | 0.484 | -0.033 | 0.016 |
| **Right I-IV** | -0.013 | 0.263 | -0.035 | 0.01 |
| **Left V** | -0.025 | 0.058 | -0.051 | 0.001 |
| **Right V** | -0.028 | **0.027** | -0.053 | -0.003 |
| **Left VI** | -0.065 | **0.021** | -0.120 | -0.01 |
| **Vermis VI** | -0.017 | **0.001** | -0.026 | -0.007 |
| **Right VI** | -0.075 | **0.003** | -0.124 | -0.026 |
| **Left Crus I** | -0.088 | **0.011** | -0.156 | -0.02 |
| **Vermis Crus I** | 0.000 | 0.269 | -0.001 | 0.000 |
| **Right Crus I** | -0.091 | **0.002** | -0.148 | -0.035 |
| **Left Crus II** | -0.036 | 0.272 | -0.101 | 0.028 |
| **Vermis Crus II** | -0.004 | 0.100 | -0.008 | 0.001 |
| **Right Crus II** | -0.048 | 0.101 | -0.105 | 0.009 |
| **Left VIIb** | -0.036 | 0.085 | -0.076 | 0.005 |
| **Vermis VIIb** | -0.001 | 0.342 | -0.003 | 0.001 |
| **Right VIIb** | -0.012 | 0.587 | -0.056 | 0.032 |
| **Left VIIIa** | -0.035 | 0.142 | -0.081 | 0.012 |
| **Vermis VIIIa** | -0.007 | 0.181 | -0.016 | 0.003 |
| **Right VIIIa** | -0.028 | 0.186 | -0.069 | 0.014 |
| **Left VIIIb** | -0.04 | **0.030** | -0.076 | -0.004 |
| **Vermis VIIIb** | -0.003 | 0.159 | -0.008 | 0.001 |
| **Right VIIIb** | -0.02 | 0.128 | -0.045 | 0.006 |
| **Left IX** | -0.023 | **0.039** | -0.046 | -0.001 |
| **Vermis IX** | -0.007 | 0.069 | -0.015 | 0.001 |
| **Right IX** | -0.028 | **0.030** | -0.053 | -0.003 |
| **Left X** | -0.003 | 0.095 | -0.007 | 0.001 |
| **Vermis X** | -0.004 | **0.007** | -0.007 | -0.001 |
| **Right X** | -0.002 | 0.190 | -0.005 | 0.001 |

GEE models were applied to examine longitudinal changes in cerebellar regional volumes within the ADD. Each model was adjusted for age, sex, years of education, APOE4 carrier status, and TIV. Regression coefficients (β), standard errors, and significance values (p < 0.05) are presented for time effects.

Abbreviations: GEE, generalized estimating equation; ADD, Alzheimer’s disease dementia; APOE4, apolipoprotein ε4; TIV, total intracranial volume

| **Region** | **CERAD Score** | **r** | **FDR corrected Q value** |
| --- | --- | --- | --- |
| ΔLeft VI | ΔCERAD Episodic | 0.538 | **<0.001** |
| ΔLeft Crus I | ΔCERAD Episodic | 0.500 | **<0.001** |
| ΔLeft Crus II | ΔCERAD Episodic | 0.370 | **0.006** |
| ΔRight VI | ΔCERAD Episodic | 0.368 | **0.006** |
| ΔRight Crus II | ΔCERAD Episodic | 0.262 | **0.049** |
| ΔLeft VI | ΔCERAD Non-Episodic | 0.129 | 0.544 |
| ΔLeft Crus I | ΔCERAD Non-Episodic | 0.017 | 0.898 |
| ΔLeft Crus II | ΔCERAD Non-Episodic | 0.148 | 0.544 |
| ΔRight VI | ΔCERAD Non-Episodic | 0.284 | **0.162** |
| ΔRight Crus II | ΔCERAD Non-Episodic | 0.105 | 0.544 |

**Supplementary Table S8. Partial correlation analysis between longitudinal changes in significant cerebellar regions and CERAD-K composite scores (episodic and non-episodic memory) in the PAD group, adjusted for covariates (age, sex, education, APOE4 carrier status and TIV)**

Spearman partial correlations were computed between Δ cerebellar volume (mm³) and Δ CERAD-K episodic memory composite scores, adjusting for age, sex, education, APOE4 carrier status, and TIV. Statistical significance was determined at p < 0.05 (false discovery rate [FDR]–corrected q < 0.05).

Abbreviations: PAD, preclinical Alzheimer’s disease; CERAD-K, Consortium to Establish a Registry for Alzheimer’s Disease–Korean version; APOE4, apolipoprotein E4; TIV, total intracranial volume; FDR, false discovery rate.

**Supplementary Table S9. Partial correlation analysis between longitudinal changes in significant cerebellar regions and CERAD-K composite scores (episodic and non-episodic memory) in the ADD group, adjusted for covariates (age, sex, education, APOE4 carrier status and TIV)**

| **Region** | **CERAD Score** | **r** | **FDR corrected Q value** |
| --- | --- | --- | --- |
| **ΔLeft VI** | **ΔCERAD Episodic** | 0.292 | 0.389 |
| **ΔRight VI** | **ΔCERAD Episodic** | 0.236 | 0.389 |
| **ΔRight Crus II** | **ΔCERAD Episodic** | 0.232 | 0.389 |
| **ΔRight Crus I** | **ΔCERAD Episodic** | 0.188 | 0.468 |
| **ΔLeft Crus I** | **ΔCERAD Episodic** | 0.183 | 0.468 |
| **ΔVermis VI** | **ΔCERAD Episodic** | 0.126 | 0.712 |
| **ΔRight IX** | **ΔCERAD Episodic** | 0.097 | 0.776 |
| **ΔRight V** | **ΔCERAD Episodic** | 0.08 | 0.776 |
| **ΔLeft IX** | **ΔCERAD Episodic** | 0.047 | 0.831 |
| **ΔVermis X** | **ΔCERAD Episodic** | 0.019 | 0.901 |
| **ΔRight Crus I** | **ΔCERAD Non-Episodic** | -0.157 | 0.877 |
| **ΔLeft IX** | **ΔCERAD Non-Episodic** | 0.152 | 0.877 |
| **ΔLeft VI** | **ΔCERAD Non-Episodic** | 0.148 | 0.877 |
| **ΔRight IX** | **ΔCERAD Non-Episodic** | 0.127 | 0.877 |
| **ΔRight VI** | **ΔCERAD Non-Episodic** | -0.081 | 0.877 |
| **ΔRight V** | **ΔCERAD Non-Episodic** | -0.051 | 0.877 |
| **ΔVermis VI** | **ΔCERAD Non-Episodic** | -0.038 | 0.877 |
| **ΔRight Crus II** | **ΔCERAD Non-Episodic** | 0.034 | 0.877 |
| **ΔLeft Crus I** | **ΔCERAD Non-Episodic** | 0.03 | 0.877 |
| **ΔVermis X** | **ΔCERAD Non-Episodic** | -0.024 | 0.877 |

Spearman partial correlations were computed between Δ cerebellar volume (mm³) and Δ CERAD-K episodic memory composite scores, adjusting for age, sex, education, APOE4 carrier status, and TIV. Statistical significance was determined at p < 0.05 (false discovery rate [FDR]–corrected q < 0.05).

Abbreviations: ADD, Alzheimer’s disease dementia; CERAD-K, Consortium to Establish a Registry for Alzheimer’s Disease–Korean version; APOE4, apolipoprotein E4; TIV, total intracranial volume; FDR, false discovery rate.

**Supplementary Table S10. Partial correlation analysis between longitudinal changes in significant cerebellar regions and NPI total, sub-scores and composite scores in the PAD group, adjusted for covariates (age, sex, education, APOE4 carrier status and TIV)**

| **NPI** | **Region** | **r** | **P value** | **FDR corrected Q value** |
| --- | --- | --- | --- | --- |
| **Δnpi_agg** | **ΔRight VI** | 0.104 | 0.442 | 0.976 |
| **Δnpi_agg** | **ΔRight Crus II** | 0.042 | 0.758 | 0.976 |
| **Δnpi_agg** | **ΔLeft Crus II** | -0.016 | 0.909 | 0.976 |
| **Δnpi_agg** | **ΔLeft VI** | 0.012 | 0.931 | 0.976 |
| **Δnpi_agg** | **ΔLeft Crus I** | 0.004 | 0.976 | 0.976 |
| **Δnpi_anx** | **ΔLeft Crus II** | -0.247 | 0.064 | 0.191 |
| **Δnpi_anx** | **ΔLeft Crus I** | -0.237 | 0.076 | 0.191 |
| **Δnpi_anx** | **ΔRight Crus II** | -0.151 | 0.262 | 0.437 |
| **Δnpi_anx** | **ΔLeft VI** | -0.094 | 0.485 | 0.607 |
| **Δnpi_anx** | **ΔRight VI** | 0.044 | 0.748 | 0.748 |
| **Δnpi_apa** | **ΔRight VI** | -0.351 | 0.007 | **0.023** |
| **Δnpi_apa** | **ΔLeft VI** | -0.342 | 0.009 | **0.023** |
| **Δnpi_apa** | **ΔLeft Crus II** | -0.246 | 0.066 | 0.109 |
| **Δnpi_apa** | **ΔLeft Crus I** | -0.193 | 0.151 | 0.189 |
| **Δnpi_apa** | **ΔRight Crus II** | -0.153 | 0.255 | 0.255 |
| **Δnpi_beha** | **ΔLeft Crus II** | -0.352 | 0.007 | **0.031** |
| **Δnpi_beha** | **ΔRight Crus II** | -0.33 | 0.012 | **0.031** |
| **Δnpi_beha** | **ΔLeft Crus I** | -0.291 | 0.028 | 0.047 |
| **Δnpi_beha** | **ΔLeft VI** | -0.12 | 0.373 | 0.445 |
| **Δnpi_beha** | **ΔRight VI** | -0.103 | 0.445 | 0.445 |
| **Δnpi_del** | **ΔRight VI** | 0.078 | 0.563 | 0.928 |
| **Δnpi_del** | **ΔLeft Crus I** | 0.068 | 0.614 | 0.928 |
| **Δnpi_del** | **ΔLeft VI** | 0.066 | 0.625 | 0.928 |
| **Δnpi_del** | **ΔRight Crus II** | 0.033 | 0.807 | 0.928 |
| **Δnpi_del** | **ΔLeft Crus II** | -0.012 | 0.928 | 0.928 |
| **Δnpi_dep** | **ΔLeft Crus I** | -0.243 | 0.068 | 0.216 |
| **Δnpi_dep** | **ΔLeft VI** | -0.21 | 0.117 | 0.216 |
| **Δnpi_dep** | **ΔLeft Crus II** | -0.203 | 0.129 | 0.216 |
| **Δnpi_dep** | **ΔRight Crus II** | -0.147 | 0.274 | 0.343 |
| **Δnpi_dep** | **ΔRight VI** | -0.117 | 0.386 | 0.386 |
| **Δnpi_disin** | **ΔRight Crus II** | 0.066 | 0.626 | 0.907 |
| **Δnpi_disin** | **ΔLeft Crus II** | 0.054 | 0.692 | 0.907 |
| **Δnpi_disin** | **ΔLeft Crus I** | 0.054 | 0.692 | 0.907 |
| **Δnpi_disin** | **ΔLeft VI** | 0.048 | 0.725 | 0.907 |
| **Δnpi_disin** | **ΔRight VI** | -0.01 | 0.939 | 0.939 |
| **Δnpi_eat** | **ΔRight Crus II** | -0.205 | 0.127 | 0.342 |
| **Δnpi_eat** | **ΔLeft Crus I** | -0.2 | 0.137 | 0.342 |
| **Δnpi_eat** | **ΔLeft Crus II** | -0.166 | 0.218 | 0.363 |
| **Δnpi_eat** | **ΔLeft VI** | -0.091 | 0.5 | 0.625 |
| **Δnpi_eat** | **ΔRight VI** | -0.053 | 0.696 | 0.696 |
| **Δnpi_eup** | **ΔRight Crus II** | -0.023 | 0.866 | 0.993 |
| **Δnpi_eup** | **ΔLeft Crus II** | -0.021 | 0.875 | 0.993 |
| **Δnpi_eup** | **ΔLeft Crus I** | -0.013 | 0.925 | 0.993 |
| **Δnpi_eup** | **ΔRight VI** | -0.012 | 0.928 | 0.993 |
| **Δnpi_eup** | **ΔLeft VI** | -0.001 | 0.993 | 0.993 |
| **Δnpi_hal** | **ΔRight Crus II** | -0.023 | 0.866 | 0.993 |
| **Δnpi_hal** | **ΔLeft Crus II** | -0.021 | 0.875 | 0.993 |
| **Δnpi_hal** | **ΔLeft Crus I** | -0.013 | 0.925 | 0.993 |
| **Δnpi_hal** | **ΔRight VI** | -0.012 | 0.928 | 0.993 |
| **Δnpi_hal** | **ΔLeft VI** | -0.001 | 0.993 | 0.993 |
| **Δnpi_hyper** | **ΔRight Crus II** | -0.101 | 0.453 | 0.869 |
| **Δnpi_hyper** | **ΔLeft Crus II** | -0.085 | 0.529 | 0.869 |
| **Δnpi_hyper** | **ΔLeft VI** | -0.064 | 0.637 | 0.869 |
| **Δnpi_hyper** | **ΔRight VI** | -0.029 | 0.829 | 0.869 |
| **Δnpi_hyper** | **ΔLeft Crus I** | -0.022 | 0.869 | 0.869 |
| **Δnpi_irri** | **ΔLeft VI** | -0.146 | 0.28 | 0.712 |
| **Δnpi_irri** | **ΔRight Crus II** | -0.142 | 0.291 | 0.712 |
| **Δnpi_irri** | **ΔLeft Crus II** | -0.085 | 0.531 | 0.712 |
| **Δnpi_irri** | **ΔRight VI** | -0.059 | 0.66 | 0.712 |
| **Δnpi_irri** | **ΔLeft Crus I** | -0.05 | 0.712 | 0.712 |
| **Δnpi_mood** | **ΔLeft Crus II** | -0.294 | 0.026 | 0.059 |
| **Δnpi_mood** | **ΔLeft Crus I** | -0.289 | 0.029 | 0.059 |
| **Δnpi_mood** | **ΔLeft VI** | -0.279 | 0.036 | 0.059 |
| **Δnpi_mood** | **ΔRight Crus II** | -0.187 | 0.165 | 0.206 |
| **Δnpi_mood** | **ΔRight VI** | -0.144 | 0.284 | 0.284 |
| **Δnpi_motor** | **ΔLeft VI** | -0.224 | 0.094 | 0.448 |
| **Δnpi_motor** | **ΔRight VI** | -0.166 | 0.217 | 0.448 |
| **Δnpi_motor** | **ΔLeft Crus I** | -0.145 | 0.28 | 0.448 |
| **Δnpi_motor** | **ΔRight Crus II** | -0.124 | 0.358 | 0.448 |
| **Δnpi_motor** | **ΔLeft Crus II** | -0.095 | 0.484 | 0.484 |
| **Δnpi_psycho** | **ΔRight VI** | 0.078 | 0.563 | 0.928 |
| **Δnpi_psycho** | **ΔLeft Crus I** | 0.068 | 0.614 | 0.928 |
| **Δnpi_psycho** | **ΔLeft VI** | 0.066 | 0.625 | 0.928 |
| **Δnpi_psycho** | **ΔRight Crus II** | 0.033 | 0.807 | 0.928 |
| **Δnpi_psycho** | **ΔLeft Crus II** | -0.012 | 0.928 | 0.928 |
| **Δnpi_sleep** | **ΔLeft Crus II** | -0.295 | 0.026 | 0.083 |
| **Δnpi_sleep** | **ΔRight Crus II** | -0.283 | 0.033 | 0.083 |
| **Δnpi_sleep** | **ΔLeft Crus I** | -0.207 | 0.123 | 0.204 |
| **Δnpi_sleep** | **ΔLeft VI** | -0.086 | 0.524 | 0.641 |
| **Δnpi_sleep** | **ΔRight VI** | -0.063 | 0.641 | 0.641 |
| **Δnpi_total** | **ΔLeft Crus II** | -0.309 | 0.019 | 0.096 |
| **Δnpi_total** | **ΔLeft Crus I** | -0.271 | 0.042 | 0.098 |
| **Δnpi_total** | **ΔRight Crus II** | -0.252 | 0.059 | 0.098 |
| **Δnpi_total** | **ΔLeft VI** | -0.222 | 0.097 | 0.122 |
| **Δnpi_total** | **ΔRight VI** | -0.095 | 0.484 | 0.484 |

Spearman partial correlations were calculated between Δ cerebellar volume (mm³) and Δ NPI domain scores in PAD, controlling for age, sex, education, APOE4 status, and TIV. Significance threshold was set at p < 0.05 (FDR-corrected q < 0.05).

Abbreviations: PAD, preclinical Alzheimer’s disease; NPI, Neuropsychiatric Inventory; APOE4, apolipoprotein E4; TIV, total intracranial volume; FDR, false discovery rate.

**Supplementary Table S11. Partial correlation analysis between longitudinal changes in significant cerebellar regions and NPI total, sub-scores and composite scores in the ADD group, adjusted for covariates (age, sex, education, APOE4 carrier status and TIV)**

| **NPI** | **Region** | **r** | **P value** | **FDR corrected Q value** |
| --- | --- | --- | --- | --- |
| **Δ npi_agg** | **Δ Left VIIIb** | -0.331 | 0.03 | 0.362 |
| **Δ npi_agg** | **Δ Right IX** | -0.254 | 0.1 | 0.602 |
| **Δ npi_agg** | **Δ Vermis X** | -0.221 | 0.155 | 0.621 |
| **Δ npi_agg** | **Δ Right V** | -0.187 | 0.23 | 0.69 |
| **Δ npi_agg** | **Δ Left IX** | -0.126 | 0.42 | 0.862 |
| **Δ npi_agg** | **Δ Right Crus I** | -0.071 | 0.652 | 0.862 |
| **Δ npi_agg** | **Δ Left Crus II** | -0.071 | 0.653 | 0.862 |
| **Δ npi_agg** | **Δ Right VI** | -0.065 | 0.68 | 0.862 |
| **Δ npi_agg** | **Δ Right Crus II** | -0.064 | 0.686 | 0.862 |
| **Δ npi_agg** | **Δ Vermis VI** | -0.043 | 0.785 | 0.862 |
| **Δ npi_agg** | **Δ Left VI** | 0.042 | 0.791 | 0.862 |
| **Δ npi_agg** | **Δ Left Crus I** | -0.013 | 0.934 | 0.934 |
| **Δ npi_anx** | **Δ Right VI** | -0.127 | 0.416 | 0.967 |
| **Δ npi_anx** | **Δ Right Crus II** | 0.1 | 0.525 | 0.967 |
| **Δ npi_anx** | **Δ Vermis X** | 0.085 | 0.588 | 0.967 |
| **Δ npi_anx** | **Δ Right IX** | -0.085 | 0.589 | 0.967 |
| **Δ npi_anx** | **Δ Left VI** | -0.064 | 0.684 | 0.967 |
| **Δ npi_anx** | **Δ Left Crus I** | -0.048 | 0.759 | 0.967 |
| **Δ npi_anx** | **Δ Right Crus I** | -0.036 | 0.818 | 0.967 |
| **Δ npi_anx** | **Δ Vermis VI** | -0.03 | 0.85 | 0.967 |
| **Δ npi_anx** | **Δ Left IX** | -0.029 | 0.855 | 0.967 |
| **Δ npi_anx** | **Δ Right V** | -0.024 | 0.877 | 0.967 |
| **Δ npi_anx** | **Δ Left VIIIb** | 0.022 | 0.89 | 0.967 |
| **Δ npi_anx** | **Δ Left Crus II** | -0.006 | 0.967 | 0.967 |
| **Δ npi_apa** | **Δ Vermis X** | 0.455 | 0.002 | **0.026** |
| **Δ npi_apa** | **Δ Right Crus II** | 0.305 | 0.047 | 0.281 |
| **Δ npi_apa** | **Δ Left VI** | 0.218 | 0.16 | 0.472 |
| **Δ npi_apa** | **Δ Vermis VI** | 0.203 | 0.191 | 0.472 |
| **Δ npi_apa** | **Δ Left VIIIb** | 0.201 | 0.197 | 0.472 |
| **Δ npi_apa** | **Δ Left Crus I** | 0.167 | 0.285 | 0.532 |
| **Δ npi_apa** | **Δ Left IX** | 0.158 | 0.31 | 0.532 |
| **Δ npi_apa** | **Δ Right V** | 0.1 | 0.522 | 0.661 |
| **Δ npi_apa** | **Δ Left Crus II** | 0.098 | 0.534 | 0.661 |
| **Δ npi_apa** | **Δ Right Crus I** | 0.093 | 0.551 | 0.661 |
| **Δ npi_apa** | **Δ Right IX** | -0.06 | 0.704 | 0.768 |
| **Δ npi_apa** | **Δ Right VI** | 0.038 | 0.808 | 0.808 |
| **Δ npi_beha** | **Δ Left IX** | 0.299 | 0.049 | 0.587 |
| **Δ npi_beha** | **Δ Vermis X** | 0.228 | 0.136 | 0.818 |
| **Δ npi_beha** | **Δ Left VIIIb** | 0.191 | 0.215 | 0.859 |
| **Δ npi_beha** | **Δ Right Crus II** | 0.151 | 0.326 | 0.97 |
| **Δ npi_beha** | **Δ Left Crus II** | -0.102 | 0.509 | 0.97 |
| **Δ npi_beha** | **Δ Vermis VI** | 0.084 | 0.587 | 0.97 |
| **Δ npi_beha** | **Δ Left Crus I** | 0.069 | 0.656 | 0.97 |
| **Δ npi_beha** | **Δ Right IX** | 0.066 | 0.67 | 0.97 |
| **Δ npi_beha** | **Δ Right V** | -0.046 | 0.767 | 0.97 |
| **Δ npi_beha** | **Δ Left VI** | 0.034 | 0.829 | 0.97 |
| **Δ npi_beha** | **Δ Right VI** | -0.022 | 0.889 | 0.97 |
| **Δ npi_beha** | **Δ Right Crus I** | 0.002 | 0.991 | 0.991 |
| **Δ npi_del** | **Δ Left Crus II** | 0.144 | 0.356 | 0.95 |
| **Δ npi_del** | **Δ Left VI** | 0.091 | 0.562 | 0.95 |
| **Δ npi_del** | **Δ Right VI** | 0.087 | 0.577 | 0.95 |
| **Δ npi_del** | **Δ Left Crus I** | 0.073 | 0.641 | 0.95 |
| **Δ npi_del** | **Δ Left VIIIb** | 0.069 | 0.662 | 0.95 |
| **Δ npi_del** | **Δ Right IX** | 0.055 | 0.728 | 0.95 |
| **Δ npi_del** | **Δ Right Crus II** | -0.052 | 0.741 | 0.95 |
| **Δ npi_del** | **Δ Left IX** | 0.049 | 0.754 | 0.95 |
| **Δ npi_del** | **Δ Vermis X** | -0.041 | 0.794 | 0.95 |
| **Δ npi_del** | **Δ Right V** | 0.021 | 0.892 | 0.95 |
| **Δ npi_del** | **Δ Right Crus I** | -0.02 | 0.9 | 0.95 |
| **Δ npi_del** | **Δ Vermis VI** | -0.01 | 0.95 | 0.95 |
| **Δ npi_dep** | **Δ Left Crus I** | -0.282 | 0.067 | 0.512 |
| **Δ npi_dep** | **Δ Left Crus II** | -0.25 | 0.106 | 0.512 |
| **Δ npi_dep** | **Δ Right Crus I** | -0.226 | 0.144 | 0.512 |
| **Δ npi_dep** | **Δ Right IX** | -0.213 | 0.171 | 0.512 |
| **Δ npi_dep** | **Δ Right Crus II** | -0.143 | 0.362 | 0.729 |
| **Δ npi_dep** | **Δ Right VI** | -0.108 | 0.49 | 0.729 |
| **Δ npi_dep** | **Δ Left VI** | -0.104 | 0.506 | 0.729 |
| **Δ npi_dep** | **Δ Left IX** | -0.094 | 0.551 | 0.729 |
| **Δ npi_dep** | **Δ Vermis X** | -0.09 | 0.568 | 0.729 |
| **Δ npi_dep** | **Δ Right V** | -0.081 | 0.608 | 0.729 |
| **Δ npi_dep** | **Δ Vermis VI** | -0.06 | 0.701 | 0.764 |
| **Δ npi_dep** | **Δ Left VIIIb** | -0.008 | 0.959 | 0.959 |
| **Δ npi_disin** | **Δ Vermis X** | -0.2 | 0.198 | 0.912 |
| **Δ npi_disin** | **Δ Vermis VI** | -0.185 | 0.236 | 0.912 |
| **Δ npi_disin** | **Δ Left VIIIb** | -0.162 | 0.301 | 0.912 |
| **Δ npi_disin** | **Δ Right V** | -0.127 | 0.418 | 0.912 |
| **Δ npi_disin** | **Δ Right VI** | -0.109 | 0.485 | 0.912 |
| **Δ npi_disin** | **Δ Right Crus I** | -0.088 | 0.577 | 0.912 |
| **Δ npi_disin** | **Δ Right Crus II** | -0.064 | 0.685 | 0.912 |
| **Δ npi_disin** | **Δ Left VI** | 0.056 | 0.719 | 0.912 |
| **Δ npi_disin** | **Δ Left Crus II** | -0.048 | 0.758 | 0.912 |
| **Δ npi_disin** | **Δ Left Crus I** | 0.048 | 0.76 | 0.912 |
| **Δ npi_disin** | **Δ Left IX** | 0.032 | 0.841 | 0.918 |
| **Δ npi_disin** | **Δ Right IX** | -0.005 | 0.975 | 0.975 |
| **Δ npi_eat** | **Δ Left VI** | -0.384 | 0.011 | 0.133 |
| **Δ npi_eat** | **Δ Left Crus I** | -0.341 | 0.025 | 0.152 |
| **Δ npi_eat** | **Δ Left Crus II** | -0.31 | 0.043 | 0.172 |
| **Δ npi_eat** | **Δ Vermis VI** | -0.237 | 0.125 | 0.362 |
| **Δ npi_eat** | **Δ Right V** | -0.223 | 0.151 | 0.362 |
| **Δ npi_eat** | **Δ Right Crus II** | -0.188 | 0.226 | 0.452 |
| **Δ npi_eat** | **Δ Right VI** | -0.142 | 0.362 | 0.621 |
| **Δ npi_eat** | **Δ Vermis X** | -0.116 | 0.46 | 0.69 |
| **Δ npi_eat** | **Δ Left IX** | 0.084 | 0.593 | 0.771 |
| **Δ npi_eat** | **Δ Right Crus I** | -0.059 | 0.705 | 0.771 |
| **Δ npi_eat** | **Δ Left VIIIb** | -0.051 | 0.745 | 0.771 |
| **Δ npi_eat** | **Δ Right IX** | -0.046 | 0.771 | 0.771 |
| **Δ npi_eup** | **Δ Left Crus II** | -0.21 | 0.177 | 0.652 |
| **Δ npi_eup** | **Δ Vermis VI** | -0.196 | 0.208 | 0.652 |
| **Δ npi_eup** | **Δ Right Crus II** | -0.165 | 0.291 | 0.652 |
| **Δ npi_eup** | **Δ Vermis X** | -0.157 | 0.314 | 0.652 |
| **Δ npi_eup** | **Δ Left Crus I** | -0.157 | 0.316 | 0.652 |
| **Δ npi_eup** | **Δ Left VIIIb** | -0.153 | 0.326 | 0.652 |
| **Δ npi_eup** | **Δ Left VI** | -0.135 | 0.387 | 0.663 |
| **Δ npi_eup** | **Δ Right V** | -0.07 | 0.654 | 0.981 |
| **Δ npi_eup** | **Δ Left IX** | 0.032 | 0.838 | 0.992 |
| **Δ npi_eup** | **Δ Right Crus I** | 0.019 | 0.904 | 0.992 |
| **Δ npi_eup** | **Δ Right IX** | -0.008 | 0.957 | 0.992 |
| **Δ npi_eup** | **Δ Right VI** | 0.001 | 0.992 | 0.992 |
| **Δ npi_hal** | **Δ Right IX** | 0.278 | 0.071 | 0.701 |
| **Δ npi_hal** | **Δ Left Crus I** | 0.195 | 0.209 | 0.701 |
| **Δ npi_hal** | **Δ Left IX** | 0.185 | 0.235 | 0.701 |
| **Δ npi_hal** | **Δ Left VI** | 0.157 | 0.313 | 0.701 |
| **Δ npi_hal** | **Δ Vermis VI** | 0.123 | 0.431 | 0.701 |
| **Δ npi_hal** | **Δ Right Crus II** | 0.122 | 0.434 | 0.701 |
| **Δ npi_hal** | **Δ Right Crus I** | -0.116 | 0.457 | 0.701 |
| **Δ npi_hal** | **Δ Right V** | -0.113 | 0.471 | 0.701 |
| **Δ npi_hal** | **Δ Left Crus II** | 0.081 | 0.604 | 0.701 |
| **Δ npi_hal** | **Δ Vermis X** | 0.073 | 0.641 | 0.701 |
| **Δ npi_hal** | **Δ Right VI** | -0.073 | 0.643 | 0.701 |
| **Δ npi_hal** | **Δ Left VIIIb** | 0.06 | 0.701 | 0.701 |
| **Δ npi_hyper** | **Δ Left VIIIb** | -0.308 | 0.042 | 0.327 |
| **Δ npi_hyper** | **Δ Vermis X** | -0.292 | 0.055 | 0.327 |
| **Δ npi_hyper** | **Δ Right IX** | -0.161 | 0.297 | 0.866 |
| **Δ npi_hyper** | **Δ Right V** | -0.148 | 0.338 | 0.866 |
| **Δ npi_hyper** | **Δ Left IX** | -0.135 | 0.382 | 0.866 |
| **Δ npi_hyper** | **Δ Right VI** | -0.111 | 0.473 | 0.866 |
| **Δ npi_hyper** | **Δ Vermis VI** | -0.082 | 0.597 | 0.866 |
| **Δ npi_hyper** | **Δ Right Crus I** | -0.073 | 0.636 | 0.866 |
| **Δ npi_hyper** | **Δ Left VI** | 0.071 | 0.649 | 0.866 |
| **Δ npi_hyper** | **Δ Right Crus II** | -0.031 | 0.843 | 0.985 |
| **Δ npi_hyper** | **Δ Left Crus II** | 0.019 | 0.903 | 0.985 |
| **Δ npi_hyper** | **Δ Left Crus I** | 0.002 | 0.992 | 0.992 |
| **Δ npi_irri** | **Δ Left VIIIb** | -0.242 | 0.117 | 0.863 |
| **Δ npi_irri** | **Δ Vermis X** | -0.175 | 0.263 | 0.863 |
| **Δ npi_irri** | **Δ Left Crus II** | 0.163 | 0.296 | 0.863 |
| **Δ npi_irri** | **Δ Left IX** | -0.163 | 0.297 | 0.863 |
| **Δ npi_irri** | **Δ Left Crus I** | 0.118 | 0.45 | 0.863 |
| **Δ npi_irri** | **Δ Right Crus II** | 0.098 | 0.53 | 0.863 |
| **Δ npi_irri** | **Δ Right VI** | -0.098 | 0.534 | 0.863 |
| **Δ npi_irri** | **Δ Right Crus I** | 0.088 | 0.575 | 0.863 |
| **Δ npi_irri** | **Δ Left VI** | 0.056 | 0.72 | 0.959 |
| **Δ npi_irri** | **Δ Right IX** | -0.028 | 0.861 | 0.998 |
| **Δ npi_irri** | **Δ Right V** | 0.014 | 0.931 | 0.998 |
| **Δ npi_irri** | **Δ Vermis VI** | 0 | 0.998 | 0.998 |
| **Δ npi_mood** | **Δ Left Crus I** | -0.238 | 0.12 | 0.779 |
| **Δ npi_mood** | **Δ Left Crus II** | -0.196 | 0.203 | 0.779 |
| **Δ npi_mood** | **Δ Right IX** | -0.182 | 0.238 | 0.779 |
| **Δ npi_mood** | **Δ Right Crus I** | -0.15 | 0.331 | 0.779 |
| **Δ npi_mood** | **Δ Right VI** | -0.14 | 0.366 | 0.779 |
| **Δ npi_mood** | **Δ Left VI** | -0.122 | 0.432 | 0.779 |
| **Δ npi_mood** | **Δ Vermis VI** | -0.116 | 0.454 | 0.779 |
| **Δ npi_mood** | **Δ Right Crus II** | -0.075 | 0.627 | 0.852 |
| **Δ npi_mood** | **Δ Left IX** | -0.073 | 0.639 | 0.852 |
| **Δ npi_mood** | **Δ Right V** | -0.045 | 0.77 | 0.903 |
| **Δ npi_mood** | **Δ Left VIIIb** | 0.034 | 0.828 | 0.903 |
| **Δ npi_mood** | **Δ Vermis X** | 0.017 | 0.911 | 0.911 |
| **Δ npi_motor** | **Δ Right VI** | 0.329 | 0.031 | 0.329 |
| **Δ npi_motor** | **Δ Left VIIIb** | 0.274 | 0.075 | 0.329 |
| **Δ npi_motor** | **Δ Left VI** | 0.253 | 0.101 | 0.329 |
| **Δ npi_motor** | **Δ Right V** | 0.244 | 0.115 | 0.329 |
| **Δ npi_motor** | **Δ Left IX** | 0.23 | 0.137 | 0.329 |
| **Δ npi_motor** | **Δ Vermis VI** | 0.193 | 0.215 | 0.401 |
| **Δ npi_motor** | **Δ Right Crus II** | 0.174 | 0.265 | 0.401 |
| **Δ npi_motor** | **Δ Vermis X** | 0.173 | 0.267 | 0.401 |
| **Δ npi_motor** | **Δ Left Crus I** | 0.152 | 0.332 | 0.443 |
| **Δ npi_motor** | **Δ Right IX** | 0.138 | 0.378 | 0.454 |
| **Δ npi_motor** | **Δ Right Crus I** | 0.113 | 0.471 | 0.513 |
| **Δ npi_motor** | **Δ Left Crus II** | 0.018 | 0.911 | 0.911 |
| **Δ npi_psycho** | **Δ Right IX** | 0.165 | 0.285 | 0.9 |
| **Δ npi_psycho** | **Δ Left Crus II** | 0.143 | 0.353 | 0.9 |
| **Δ npi_psycho** | **Δ Left IX** | 0.136 | 0.379 | 0.9 |
| **Δ npi_psycho** | **Δ Left VIIIb** | 0.134 | 0.386 | 0.9 |
| **Δ npi_psycho** | **Δ Left VI** | 0.118 | 0.444 | 0.9 |
| **Δ npi_psycho** | **Δ Left Crus I** | 0.117 | 0.45 | 0.9 |
| **Δ npi_psycho** | **Δ Right VI** | 0.092 | 0.554 | 0.916 |
| **Δ npi_psycho** | **Δ Right V** | 0.064 | 0.682 | 0.916 |
| **Δ npi_psycho** | **Δ Vermis X** | 0.047 | 0.76 | 0.916 |
| **Δ npi_psycho** | **Δ Vermis VI** | 0.039 | 0.801 | 0.916 |
| **Δ npi_psycho** | **Δ Right Crus II** | -0.022 | 0.887 | 0.916 |
| **Δ npi_psycho** | **Δ Right Crus I** | -0.016 | 0.916 | 0.916 |
| **Δ npi_sleep** | **Δ Left IX** | 0.222 | 0.152 | 0.897 |
| **Δ npi_sleep** | **Δ Right V** | -0.116 | 0.458 | 0.897 |
| **Δ npi_sleep** | **Δ Right Crus I** | -0.114 | 0.468 | 0.897 |
| **Δ npi_sleep** | **Δ Right VI** | -0.096 | 0.542 | 0.897 |
| **Δ npi_sleep** | **Δ Vermis X** | -0.09 | 0.567 | 0.897 |
| **Δ npi_sleep** | **Δ Vermis VI** | -0.082 | 0.602 | 0.897 |
| **Δ npi_sleep** | **Δ Right Crus II** | -0.07 | 0.656 | 0.897 |
| **Δ npi_sleep** | **Δ Left VI** | -0.058 | 0.71 | 0.897 |
| **Δ npi_sleep** | **Δ Left Crus II** | -0.052 | 0.738 | 0.897 |
| **Δ npi_sleep** | **Δ Left Crus I** | 0.047 | 0.767 | 0.897 |
| **Δ npi_sleep** | **Δ Left VIIIb** | 0.035 | 0.823 | 0.897 |
| **Δ npi_sleep** | **Δ Right IX** | 0.015 | 0.926 | 0.926 |
| **Δ npi_total** | **Δ Right Crus I** | -0.178 | 0.255 | 0.912 |
| **Δ npi_total** | **Δ Right VI** | -0.125 | 0.423 | 0.912 |
| **Δ npi_total** | **Δ Left Crus II** | -0.122 | 0.436 | 0.912 |
| **Δ npi_total** | **Δ Left IX** | 0.118 | 0.452 | 0.912 |
| **Δ npi_total** | **Δ Right V** | -0.108 | 0.49 | 0.912 |
| **Δ npi_total** | **Δ Vermis X** | 0.068 | 0.664 | 0.912 |
| **Δ npi_total** | **Δ Right IX** | -0.064 | 0.682 | 0.912 |
| **Δ npi_total** | **Δ Left Crus I** | -0.033 | 0.834 | 0.912 |
| **Δ npi_total** | **Δ Right Crus II** | 0.03 | 0.85 | 0.912 |
| **Δ npi_total** | **Δ Left VIIIb** | 0.026 | 0.869 | 0.912 |
| **Δ npi_total** | **Δ Left VI** | 0.018 | 0.908 | 0.912 |
| **Δ npi_total** | **Δ Vermis VI** | -0.017 | 0.912 | 0.912 |

Spearman partial correlations were calculated between Δ cerebellar volume (mm³) and Δ NPI domain scores in ADD, controlling for age, sex, education, APOE4 status, and TIV. Significance threshold was set at p < 0.05 (FDR-corrected q < 0.05).

Abbreviations: ADD, Alzheimer’s disease dementia; NPI, Neuropsychiatric Inventory; APOE4, apolipoprotein E4; TIV, total intracranial volume; FDR, false discovery rate.

**Supplementary Table S12. Partial correlation analysis between longitudinal changes in significant cerebellar regions and cortical volume in the PAD group, adjusted for covariates (age, sex, education, APOE4 carrier status and TIV)**

| **FSF ROI** | **Region** | **r** | **P value** | **FDR corrected Q value** |
| --- | --- | --- | --- | --- |
| **ΔLateral Orbitofrontal Gyrus (LH)** | **ΔLeft VI** | 0.221 | 0.099 | 0.389 |
| **ΔLateral Orbitofrontal Gyrus (LH)** | **ΔRight VI** | 0.191 | 0.156 | 0.389 |
| **ΔLateral Orbitofrontal Gyrus (LH)** | **ΔLeft Crus I** | 0.153 | 0.256 | 0.426 |
| **ΔLateral Orbitofrontal Gyrus (LH)** | **ΔLeft Crus II** | 0.101 | 0.456 | 0.456 |
| **ΔLateral Orbitofrontal Gyrus (LH)** | **ΔRight Crus II** | 0.111 | 0.411 | 0.456 |
| **ΔLateral Orbitofrontal Gyrus (RH)** | **ΔLeft VI** | 0.251 | 0.06 | 0.075 |
| **ΔLateral Orbitofrontal Gyrus (RH)** | **ΔRight VI** | 0.159 | 0.239 | 0.239 |
| **ΔLateral Orbitofrontal Gyrus (RH)** | **ΔLeft Crus I** | 0.283 | 0.033 | 0.075 |
| **ΔLateral Orbitofrontal Gyrus (RH)** | **ΔLeft Crus II** | 0.255 | 0.056 | 0.075 |
| **ΔLateral Orbitofrontal Gyrus (RH)** | **ΔRight Crus II** | 0.351 | 0.007 | **0.037** |
| **ΔMedial Orbitofrontal Gyrus (LH)** | **ΔLeft VI** | 0.404 | 0.002 | **0.009** |
| **ΔMedial Orbitofrontal Gyrus (LH)** | **ΔRight VI** | 0.29 | 0.029 | 0.072 |
| **ΔMedial Orbitofrontal Gyrus (LH)** | **ΔLeft Crus I** | 0.214 | 0.11 | 0.137 |
| **ΔMedial Orbitofrontal Gyrus (LH)** | **ΔLeft Crus II** | 0.229 | 0.086 | 0.137 |
| **ΔMedial Orbitofrontal Gyrus (LH)** | **ΔRight Crus II** | 0.133 | 0.323 | 0.323 |
| **ΔMedial Orbitofrontal Gyrus (RH)** | **ΔLeft VI** | 0.031 | 0.817 | 0.817 |
| **ΔMedial Orbitofrontal Gyrus (RH)** | **ΔRight VI** | -0.046 | 0.734 | 0.817 |
| **ΔMedial Orbitofrontal Gyrus (RH)** | **ΔLeft Crus I** | 0.155 | 0.249 | 0.817 |
| **ΔMedial Orbitofrontal Gyrus (RH)** | **ΔLeft Crus II** | 0.085 | 0.528 | 0.817 |
| **ΔMedial Orbitofrontal Gyrus (RH)** | **ΔRight Crus II** | 0.083 | 0.54 | 0.817 |
| **ΔRostral Middle Frontal Gyrus (LH)** | **ΔLeft VI** | 0.113 | 0.402 | 0.776 |
| **ΔRostral Middle Frontal Gyrus (LH)** | **ΔRight VI** | 0.067 | 0.621 | 0.776 |
| **ΔRostral Middle Frontal Gyrus (LH)** | **ΔLeft Crus I** | 0.103 | 0.447 | 0.776 |
| **ΔRostral Middle Frontal Gyrus (LH)** | **ΔLeft Crus II** | 0.011 | 0.935 | 0.935 |
| **ΔRostral Middle Frontal Gyrus (LH)** | **ΔRight Crus II** | -0.088 | 0.516 | 0.776 |
| **ΔRostral Middle Frontal Gyrus (RH)** | **ΔLeft VI** | 0.055 | 0.684 | 0.911 |
| **ΔRostral Middle Frontal Gyrus (RH)** | **ΔRight VI** | -0.087 | 0.518 | 0.911 |
| **ΔRostral Middle Frontal Gyrus (RH)** | **ΔLeft Crus I** | 0.096 | 0.477 | 0.911 |
| **ΔRostral Middle Frontal Gyrus (RH)** | **ΔLeft Crus II** | 0.015 | 0.911 | 0.911 |
| **ΔRostral Middle Frontal Gyrus (RH)** | **ΔRight Crus II** | -0.034 | 0.801 | 0.911 |
| **ΔCaudal Middle Frontal Gyrus (LH)** | **ΔLeft VI** | 0.088 | 0.517 | 0.805 |
| **ΔCaudal Middle Frontal Gyrus (LH)** | **ΔRight VI** | -0.033 | 0.805 | 0.805 |
| **ΔCaudal Middle Frontal Gyrus (LH)** | **ΔLeft Crus I** | 0.067 | 0.619 | 0.805 |
| **ΔCaudal Middle Frontal Gyrus (LH)** | **ΔLeft Crus II** | 0.1 | 0.459 | 0.805 |
| **ΔCaudal Middle Frontal Gyrus (LH)** | **ΔRight Crus II** | -0.041 | 0.761 | 0.805 |
| **ΔCaudal Middle Frontal Gyrus (RH)** | **ΔLeft VI** | 0.183 | 0.174 | 0.864 |
| **ΔCaudal Middle Frontal Gyrus (RH)** | **ΔRight VI** | 0.106 | 0.434 | 0.864 |
| **ΔCaudal Middle Frontal Gyrus (RH)** | **ΔLeft Crus I** | 0.058 | 0.668 | 0.864 |
| **ΔCaudal Middle Frontal Gyrus (RH)** | **ΔLeft Crus II** | 0.002 | 0.991 | 0.991 |
| **ΔCaudal Middle Frontal Gyrus (RH)** | **ΔRight Crus II** | -0.054 | 0.691 | 0.864 |
| **ΔSuperior Frontal Gyrus (LH)** | **ΔLeft VI** | 0.286 | 0.031 | 0.156 |
| **ΔSuperior Frontal Gyrus (LH)** | **ΔRight VI** | 0.235 | 0.078 | 0.195 |
| **ΔSuperior Frontal Gyrus (LH)** | **ΔLeft Crus I** | 0.158 | 0.241 | 0.402 |
| **ΔSuperior Frontal Gyrus (LH)** | **ΔLeft Crus II** | 0.133 | 0.324 | 0.405 |
| **ΔSuperior Frontal Gyrus (LH)** | **ΔRight Crus II** | -0.001 | 0.994 | 0.994 |
| **ΔSuperior Frontal Gyrus (RH)** | **ΔLeft VI** | 0.152 | 0.258 | 0.645 |
| **ΔSuperior Frontal Gyrus (RH)** | **ΔRight VI** | 0.227 | 0.09 | 0.448 |
| **ΔSuperior Frontal Gyrus (RH)** | **ΔLeft Crus I** | -0.011 | 0.932 | 0.932 |
| **ΔSuperior Frontal Gyrus (RH)** | **ΔLeft Crus II** | -0.029 | 0.832 | 0.932 |
| **ΔSuperior Frontal Gyrus (RH)** | **ΔRight Crus II** | -0.108 | 0.423 | 0.706 |
| **ΔInferior Temporal Gyrus (LH)** | **ΔLeft VI** | 0.568 | <0.001 | **<0.001** |
| **ΔInferior Temporal Gyrus (LH)** | **ΔRight VI** | 0.432 | 0.001 | **0.002** |
| **ΔInferior Temporal Gyrus (LH)** | **ΔLeft Crus I** | 0.335 | 0.011 | **0.018** |
| **ΔInferior Temporal Gyrus (LH)** | **ΔLeft Crus II** | 0.279 | 0.035 | **0.044** |
| **ΔInferior Temporal Gyrus (LH)** | **ΔRight Crus II** | 0.19 | 0.157 | 0.157 |
| **ΔInferior Temporal Gyrus (RH)** | **ΔLeft VI** | 0.365 | 0.005 | **0.013** |
| **ΔInferior Temporal Gyrus (RH)** | **ΔRight VI** | 0.389 | 0.003 | **0.013** |
| **ΔInferior Temporal Gyrus (RH)** | **ΔLeft Crus I** | 0.153 | 0.256 | 0.320 |
| **ΔInferior Temporal Gyrus (RH)** | **ΔLeft Crus II** | 0.057 | 0.675 | 0.675 |
| **ΔInferior Temporal Gyrus (RH)** | **ΔRight Crus II** | 0.173 | 0.199 | 0.320 |
| **ΔMiddle Temporal Gyrus (LH)** | **ΔLeft VI** | 0.327 | 0.013 | 0.066 |
| **ΔMiddle Temporal Gyrus (LH)** | **ΔRight VI** | 0.086 | 0.523 | 0.637 |
| **ΔMiddle Temporal Gyrus (LH)** | **ΔLeft Crus I** | 0.159 | 0.236 | 0.590 |
| **ΔMiddle Temporal Gyrus (LH)** | **ΔLeft Crus II** | 0.107 | 0.429 | 0.637 |
| **ΔMiddle Temporal Gyrus (LH)** | **ΔRight Crus II** | -0.064 | 0.637 | 0.637 |
| **ΔMiddle Temporal Gyrus (RH)** | **ΔLeft VI** | 0.178 | 0.186 | 0.540 |
| **ΔMiddle Temporal Gyrus (RH)** | **ΔRight VI** | 0.166 | 0.216 | 0.540 |
| **ΔMiddle Temporal Gyrus (RH)** | **ΔLeft Crus I** | 0 | 0.998 | 0.998 |
| **ΔMiddle Temporal Gyrus (RH)** | **ΔLeft Crus II** | 0.097 | 0.475 | 0.594 |
| **ΔMiddle Temporal Gyrus (RH)** | **ΔRight Crus II** | 0.129 | 0.338 | 0.563 |
| **ΔSuperior Temporal Gyrus (LH)** | **ΔLeft VI** | 0.435 | 0.001 | **0.004** |
| **ΔSuperior Temporal Gyrus (LH)** | **ΔRight VI** | 0.355 | 0.007 | **0.017** |
| **ΔSuperior Temporal Gyrus (LH)** | **ΔLeft Crus I** | 0.32 | 0.015 | **0.026** |
| **ΔSuperior Temporal Gyrus (LH)** | **ΔLeft Crus II** | 0.152 | 0.259 | 0.324 |
| **ΔSuperior Temporal Gyrus (LH)** | **ΔRight Crus II** | 0.118 | 0.383 | 0.383 |
| **ΔSuperior Temporal Gyrus (RH)** | **ΔLeft VI** | 0.355 | 0.007 | **0.017** |
| **ΔSuperior Temporal Gyrus (RH)** | **ΔRight VI** | 0.354 | 0.007 | **0.017** |
| **ΔSuperior Temporal Gyrus (RH)** | **ΔLeft Crus I** | 0.212 | 0.114 | 0.114 |
| **ΔSuperior Temporal Gyrus (RH)** | **ΔLeft Crus II** | 0.219 | 0.102 | 0.114 |
| **ΔSuperior Temporal Gyrus (RH)** | **ΔRight Crus II** | 0.28 | 0.035 | 0.058 |
| **ΔTransverse Temporal Gyrus (LH)** | **ΔLeft VI** | 0.268 | 0.044 | 0.110 |
| **ΔTransverse Temporal Gyrus (LH)** | **ΔRight VI** | 0.342 | 0.009 | **0.046** |
| **ΔTransverse Temporal Gyrus (LH)** | **ΔLeft Crus I** | 0.13 | 0.337 | 0.337 |
| **ΔTransverse Temporal Gyrus (LH)** | **ΔLeft Crus II** | 0.152 | 0.257 | 0.322 |
| **ΔTransverse Temporal Gyrus (LH)** | **ΔRight Crus II** | 0.159 | 0.237 | 0.322 |
| **ΔTransverse Temporal Gyrus (RH)** | **ΔLeft VI** | 0.203 | 0.13 | 0.326 |
| **ΔTransverse Temporal Gyrus (RH)** | **ΔRight VI** | 0.222 | 0.097 | 0.326 |
| **ΔTransverse Temporal Gyrus (RH)** | **ΔLeft Crus I** | 0.123 | 0.361 | 0.602 |
| **ΔTransverse Temporal Gyrus (RH)** | **ΔLeft Crus II** | -0.08 | 0.556 | 0.695 |
| **ΔTransverse Temporal Gyrus (RH)** | **ΔRight Crus II** | 0.028 | 0.834 | 0.834 |
| **ΔCaudal Anterior Cingulate Cortex (LH)** | **ΔLeft VI** | 0.166 | 0.217 | 0.712 |
| **ΔCaudal Anterior Cingulate Cortex (LH)** | **ΔRight VI** | 0.013 | 0.925 | 0.925 |
| **ΔCaudal Anterior Cingulate Cortex (LH)** | **ΔLeft Crus I** | 0.107 | 0.427 | 0.712 |
| **ΔCaudal Anterior Cingulate Cortex (LH)** | **ΔLeft Crus II** | -0.024 | 0.86 | 0.925 |
| **ΔCaudal Anterior Cingulate Cortex (LH)** | **ΔRight Crus II** | -0.113 | 0.402 | 0.712 |
| **ΔCaudal Anterior Cingulate Cortex (RH)** | **ΔLeft VI** | 0.199 | 0.137 | 0.368 |
| **ΔCaudal Anterior Cingulate Cortex (RH)** | **ΔRight VI** | 0.077 | 0.571 | 0.600 |
| **ΔCaudal Anterior Cingulate Cortex (RH)** | **ΔLeft Crus I** | 0.071 | 0.6 | 0.600 |
| **ΔCaudal Anterior Cingulate Cortex (RH)** | **ΔLeft Crus II** | 0.105 | 0.435 | 0.600 |
| **ΔCaudal Anterior Cingulate Cortex (RH)** | **ΔRight Crus II** | 0.194 | 0.147 | 0.368 |
| **ΔRostral Anterior Cingulate Cortex (LH)** | **ΔLeft VI** | -0.057 | 0.676 | 0.747 |
| **ΔRostral Anterior Cingulate Cortex (LH)** | **ΔRight VI** | -0.054 | 0.691 | 0.747 |
| **ΔRostral Anterior Cingulate Cortex (LH)** | **ΔLeft Crus I** | -0.044 | 0.747 | 0.747 |
| **ΔRostral Anterior Cingulate Cortex (LH)** | **ΔLeft Crus II** | -0.143 | 0.287 | 0.747 |
| **ΔRostral Anterior Cingulate Cortex (LH)** | **ΔRight Crus II** | -0.116 | 0.39 | 0.747 |
| **ΔRostral Anterior Cingulate Cortex (RH)** | **ΔLeft VI** | -0.05 | 0.711 | 0.711 |
| **ΔRostral Anterior Cingulate Cortex (RH)** | **ΔRight VI** | -0.158 | 0.239 | 0.369 |
| **ΔRostral Anterior Cingulate Cortex (RH)** | **ΔLeft Crus I** | -0.246 | 0.065 | 0.163 |
| **ΔRostral Anterior Cingulate Cortex (RH)** | **ΔLeft Crus II** | -0.318 | 0.016 | 0.079 |
| **ΔRostral Anterior Cingulate Cortex (RH)** | **ΔRight Crus II** | -0.141 | 0.295 | 0.369 |
| **ΔIsthmus Cingulate Cortex (LH)** | **ΔLeft VI** | 0.378 | 0.004 | **0.019** |
| **ΔIsthmus Cingulate Cortex (LH)** | **ΔRight VI** | 0.304 | 0.022 | **0.028** |
| **ΔIsthmus Cingulate Cortex (LH)** | **ΔLeft Crus I** | 0.301 | 0.023 | **0.028** |
| **ΔIsthmus Cingulate Cortex (LH)** | **ΔLeft Crus II** | 0.259 | 0.052 | 0.052 |
| **ΔIsthmus Cingulate Cortex (LH)** | **ΔRight Crus II** | 0.333 | 0.011 | **0.028** |
| **ΔIsthmus Cingulate Cortex (RH)** | **ΔLeft VI** | 0.19 | 0.157 | 0.392 |
| **ΔIsthmus Cingulate Cortex (RH)** | **ΔRight VI** | 0.118 | 0.383 | 0.638 |
| **ΔIsthmus Cingulate Cortex (RH)** | **ΔLeft Crus I** | 0.028 | 0.838 | 0.838 |
| **ΔIsthmus Cingulate Cortex (RH)** | **ΔLeft Crus II** | 0.086 | 0.523 | 0.654 |
| **ΔIsthmus Cingulate Cortex (RH)** | **ΔRight Crus II** | 0.251 | 0.06 | 0.301 |
| **ΔPosterior Cingulate Cortex (LH)** | **ΔLeft VI** | 0.245 | 0.067 | 0.333 |
| **ΔPosterior Cingulate Cortex (LH)** | **ΔRight VI** | 0.181 | 0.178 | 0.446 |
| **ΔPosterior Cingulate Cortex (LH)** | **ΔLeft Crus I** | 0.068 | 0.615 | 0.784 |
| **ΔPosterior Cingulate Cortex (LH)** | **ΔLeft Crus II** | -0.064 | 0.636 | 0.784 |
| **ΔPosterior Cingulate Cortex (LH)** | **ΔRight Crus II** | -0.037 | 0.784 | 0.784 |
| **ΔPosterior Cingulate Cortex (RH)** | **ΔLeft VI** | 0.176 | 0.19 | 0.815 |
| **ΔPosterior Cingulate Cortex (RH)** | **ΔRight VI** | 0.093 | 0.489 | 0.815 |
| **ΔPosterior Cingulate Cortex (RH)** | **ΔLeft Crus I** | 0.001 | 0.997 | 0.997 |
| **ΔPosterior Cingulate Cortex (RH)** | **ΔLeft Crus II** | -0.013 | 0.923 | 0.997 |
| **ΔPosterior Cingulate Cortex (RH)** | **ΔRight Crus II** | 0.095 | 0.483 | 0.815 |
| **ΔParahippocampal Gyrus (LH)** | **ΔLeft VI** | 0.286 | 0.031 | 0.077 |
| **ΔParahippocampal Gyrus (LH)** | **ΔRight VI** | 0.304 | 0.021 | 0.077 |
| **ΔParahippocampal Gyrus (LH)** | **ΔLeft Crus I** | 0.156 | 0.246 | 0.246 |
| **ΔParahippocampal Gyrus (LH)** | **ΔLeft Crus II** | 0.212 | 0.113 | 0.141 |
| **ΔParahippocampal Gyrus (LH)** | **ΔRight Crus II** | 0.262 | 0.049 | 0.081 |
| **ΔParahippocampal Gyrus (RH)** | **ΔLeft VI** | 0.368 | 0.005 | **0.015** |
| **ΔParahippocampal Gyrus (RH)** | **ΔRight VI** | 0.358 | 0.006 | **0.015** |
| **ΔParahippocampal Gyrus (RH)** | **ΔLeft Crus I** | 0.219 | 0.101 | 0.127 |
| **ΔParahippocampal Gyrus (RH)** | **ΔLeft Crus II** | 0.179 | 0.182 | 0.182 |
| **ΔParahippocampal Gyrus (RH)** | **ΔRight Crus II** | 0.279 | 0.035 | 0.059 |
| **ΔInsular Cortex (LH)** | **ΔLeft VI** | 0.235 | 0.079 | 0.395 |
| **ΔInsular Cortex (LH)** | **ΔRight VI** | 0.07 | 0.604 | 0.951 |
| **ΔInsular Cortex (LH)** | **ΔLeft Crus I** | 0.104 | 0.44 | 0.951 |
| **ΔInsular Cortex (LH)** | **ΔLeft Crus II** | 0.008 | 0.951 | 0.951 |
| **ΔInsular Cortex (LH)** | **ΔRight Crus II** | -0.04 | 0.767 | 0.951 |
| **ΔInsular Cortex (RH)** | **ΔLeft VI** | 0.219 | 0.102 | 0.444 |
| **ΔInsular Cortex (RH)** | **ΔRight VI** | 0.181 | 0.178 | 0.444 |
| **ΔInsular Cortex (RH)** | **ΔLeft Crus I** | 0.089 | 0.512 | 0.854 |
| **ΔInsular Cortex (RH)** | **ΔLeft Crus II** | -0.02 | 0.88 | 0.88 |
| **ΔInsular Cortex (RH)** | **ΔRight Crus II** | -0.045 | 0.742 | 0.88 |

Spearman partial correlations were computed between Δ cerebellar volume (mm³) and Δ cortical volume (mm³) in selected FSF-defined regions, adjusting for age, sex, education, APOE4 carrier status, and TIV. Statistical significance was determined at p < 0.05 (FDR-corrected q < 0.05).

Abbreviations: FSF, freesurfer;, ROI, region of interest; PAD, preclinical Alzheimer’s disease; APOE4, apolipoprotein E4; TIV, total intracranial volume; FDR, false discovery rate.

**Supplementary Table S13. Partial correlation analysis between longitudinal changes in significant cerebellar regions and cortical volume in the ADD group, adjusted for covariates (age, sex, education, APOE4 carrier status and TIV)**

| **FSF ROI** | **Region** | **r** | **P value** | **FDR corrected Q value** |
| --- | --- | --- | --- | --- |
| **ΔLateral Orbitofrontal Gyrus (LH)** | **ΔRight V** | -0.083 | 0.592 | 0.891 |
| **ΔLateral Orbitofrontal Gyrus (LH)** | **ΔLeft VI** | 0.039 | 0.802 | 0.891 |
| **ΔLateral Orbitofrontal Gyrus (LH)** | **ΔVermis VI** | -0.062 | 0.69 | 0.891 |
| **ΔLateral Orbitofrontal Gyrus (LH)** | **ΔRight VI** | -0.05 | 0.747 | 0.891 |
| **ΔLateral Orbitofrontal Gyrus (LH)** | **ΔLeft Crus I** | -0.044 | 0.779 | 0.891 |
| **ΔLateral Orbitofrontal Gyrus (LH)** | **ΔRight Crus I** | 0.014 | 0.93 | 0.93 |
| **ΔLateral Orbitofrontal Gyrus (LH)** | **ΔLeft VIIIb** | -0.208 | 0.174 | 0.879 |
| **ΔLateral Orbitofrontal Gyrus (LH)** | **ΔLeft IX** | -0.132 | 0.394 | 0.891 |
| **ΔLateral Orbitofrontal Gyrus (LH)** | **ΔRight IX** | -0.208 | 0.176 | 0.879 |
| **ΔLateral Orbitofrontal Gyrus (LH)** | **ΔVermis X** | -0.075 | 0.628 | 0.891 |
| **ΔLateral Orbitofrontal Gyrus (RH)** | **ΔRight V** | 0.148 | 0.338 | 0.588 |
| **ΔLateral Orbitofrontal Gyrus (RH)** | **ΔLeft VI** | 0.259 | 0.09 | 0.588 |
| **ΔLateral Orbitofrontal Gyrus (RH)** | **ΔVermis VI** | 0.149 | 0.336 | 0.588 |
| **ΔLateral Orbitofrontal Gyrus (RH)** | **ΔRight VI** | 0.16 | 0.298 | 0.588 |
| **ΔLateral Orbitofrontal Gyrus (RH)** | **ΔLeft Crus I** | 0.143 | 0.353 | 0.588 |
| **ΔLateral Orbitofrontal Gyrus (RH)** | **ΔRight Crus I** | 0.149 | 0.335 | 0.588 |
| **ΔLateral Orbitofrontal Gyrus (RH)** | **ΔLeft VIIIb** | -0.035 | 0.819 | 0.966 |
| **ΔLateral Orbitofrontal Gyrus (RH)** | **ΔLeft IX** | 0.007 | 0.966 | 0.966 |
| **ΔLateral Orbitofrontal Gyrus (RH)** | **ΔRight IX** | -0.056 | 0.716 | 0.966 |
| **ΔLateral Orbitofrontal Gyrus (RH)** | **ΔVermis X** | 0.022 | 0.888 | 0.966 |
| **ΔMedial Orbitofrontal Gyrus (LH)** | **ΔRight V** | -0.127 | 0.413 | 0.689 |
| **ΔMedial Orbitofrontal Gyrus (LH)** | **ΔLeft VI** | -0.053 | 0.731 | 0.904 |
| **ΔMedial Orbitofrontal Gyrus (LH)** | **ΔVermis VI** | -0.041 | 0.789 | 0.904 |
| **ΔMedial Orbitofrontal Gyrus (LH)** | **ΔRight VI** | -0.037 | 0.814 | 0.904 |
| **ΔMedial Orbitofrontal Gyrus (LH)** | **ΔLeft Crus I** | -0.127 | 0.413 | 0.689 |
| **ΔMedial Orbitofrontal Gyrus (LH)** | **ΔRight Crus I** | 0.01 | 0.949 | 0.949 |
| **ΔMedial Orbitofrontal Gyrus (LH)** | **ΔLeft VIIIb** | -0.202 | 0.188 | 0.469 |
| **ΔMedial Orbitofrontal Gyrus (LH)** | **ΔLeft IX** | -0.218 | 0.155 | 0.469 |
| **ΔMedial Orbitofrontal Gyrus (LH)** | **ΔRight IX** | -0.411 | 0.006 | 0.056 |
| **ΔMedial Orbitofrontal Gyrus (LH)** | **ΔVermis X** | -0.202 | 0.188 | 0.469 |
| **ΔMedial Orbitofrontal Gyrus (RH)** | **ΔRight V** | 0.33 | 0.029 | 0.096 |
| **ΔMedial Orbitofrontal Gyrus (RH)** | **ΔLeft VI** | 0.39 | 0.009 | 0.089 |
| **ΔMedial Orbitofrontal Gyrus (RH)** | **ΔVermis VI** | 0.223 | 0.145 | 0.29 |
| **ΔMedial Orbitofrontal Gyrus (RH)** | **ΔRight VI** | 0.336 | 0.026 | 0.096 |
| **ΔMedial Orbitofrontal Gyrus (RH)** | **ΔLeft Crus I** | 0.174 | 0.259 | 0.432 |
| **ΔMedial Orbitofrontal Gyrus (RH)** | **ΔRight Crus I** | 0.288 | 0.058 | 0.145 |
| **ΔMedial Orbitofrontal Gyrus (RH)** | **ΔLeft VIIIb** | 0.024 | 0.877 | 0.877 |
| **ΔMedial Orbitofrontal Gyrus (RH)** | **ΔLeft IX** | 0.03 | 0.846 | 0.877 |
| **ΔMedial Orbitofrontal Gyrus (RH)** | **ΔRight IX** | 0.153 | 0.322 | 0.46 |
| **ΔMedial Orbitofrontal Gyrus (RH)** | **ΔVermis X** | 0.032 | 0.836 | 0.877 |
| **ΔRostral Middle Frontal Gyrus (LH)** | **ΔRight V** | 0.081 | 0.6 | 0.758 |
| **ΔRostral Middle Frontal Gyrus (LH)** | **ΔLeft VI** | 0.172 | 0.265 | 0.758 |
| **ΔRostral Middle Frontal Gyrus (LH)** | **ΔVermis VI** | -0.019 | 0.903 | 0.903 |
| **ΔRostral Middle Frontal Gyrus (LH)** | **ΔRight VI** | 0.09 | 0.559 | 0.758 |
| **ΔRostral Middle Frontal Gyrus (LH)** | **ΔLeft Crus I** | 0.091 | 0.558 | 0.758 |
| **ΔRostral Middle Frontal Gyrus (LH)** | **ΔRight Crus I** | 0.141 | 0.362 | 0.758 |
| **ΔRostral Middle Frontal Gyrus (LH)** | **ΔLeft VIIIb** | -0.093 | 0.546 | 0.758 |
| **ΔRostral Middle Frontal Gyrus (LH)** | **ΔLeft IX** | 0.08 | 0.607 | 0.758 |
| **ΔRostral Middle Frontal Gyrus (LH)** | **ΔRight IX** | -0.035 | 0.824 | 0.903 |
| **ΔRostral Middle Frontal Gyrus (LH)** | **ΔVermis X** | -0.128 | 0.408 | 0.758 |
| **ΔRostral Middle Frontal Gyrus (RH)** | **ΔRight V** | 0.093 | 0.547 | 0.819 |
| **ΔRostral Middle Frontal Gyrus (RH)** | **ΔLeft VI** | -0.006 | 0.968 | 0.968 |
| **ΔRostral Middle Frontal Gyrus (RH)** | **ΔVermis VI** | -0.056 | 0.717 | 0.819 |
| **ΔRostral Middle Frontal Gyrus (RH)** | **ΔRight VI** | 0.052 | 0.737 | 0.819 |
| **ΔRostral Middle Frontal Gyrus (RH)** | **ΔLeft Crus I** | -0.155 | 0.316 | 0.819 |
| **ΔRostral Middle Frontal Gyrus (RH)** | **ΔRight Crus I** | 0.098 | 0.527 | 0.819 |
| **ΔRostral Middle Frontal Gyrus (RH)** | **ΔLeft VIIIb** | -0.14 | 0.364 | 0.819 |
| **ΔRostral Middle Frontal Gyrus (RH)** | **ΔLeft IX** | -0.065 | 0.677 | 0.819 |
| **ΔRostral Middle Frontal Gyrus (RH)** | **ΔRight IX** | -0.107 | 0.491 | 0.819 |
| **ΔRostral Middle Frontal Gyrus (RH)** | **ΔVermis X** | -0.103 | 0.506 | 0.819 |
| **ΔCaudal Middle Frontal Gyrus (LH)** | **ΔRight V** | 0.087 | 0.574 | 0.729 |
| **ΔCaudal Middle Frontal Gyrus (LH)** | **ΔLeft VI** | 0.128 | 0.406 | 0.729 |
| **ΔCaudal Middle Frontal Gyrus (LH)** | **ΔVermis VI** | -0.113 | 0.467 | 0.729 |
| **ΔCaudal Middle Frontal Gyrus (LH)** | **ΔRight VI** | -0.031 | 0.839 | 0.839 |
| **ΔCaudal Middle Frontal Gyrus (LH)** | **ΔLeft Crus I** | 0.039 | 0.802 | 0.839 |
| **ΔCaudal Middle Frontal Gyrus (LH)** | **ΔRight Crus I** | -0.085 | 0.583 | 0.729 |
| **ΔCaudal Middle Frontal Gyrus (LH)** | **ΔLeft VIIIb** | -0.268 | 0.078 | 0.325 |
| **ΔCaudal Middle Frontal Gyrus (LH)** | **ΔLeft IX** | -0.253 | 0.098 | 0.325 |
| **ΔCaudal Middle Frontal Gyrus (LH)** | **ΔRight IX** | -0.28 | 0.065 | 0.325 |
| **ΔCaudal Middle Frontal Gyrus (LH)** | **ΔVermis X** | -0.157 | 0.309 | 0.729 |
| **ΔCaudal Middle Frontal Gyrus (RH)** | **ΔRight V** | 0.143 | 0.355 | 0.507 |
| **ΔCaudal Middle Frontal Gyrus (RH)** | **ΔLeft VI** | 0.371 | 0.013 | 0.133 |
| **ΔCaudal Middle Frontal Gyrus (RH)** | **ΔVermis VI** | -0.039 | 0.801 | 0.801 |
| **ΔCaudal Middle Frontal Gyrus (RH)** | **ΔRight VI** | 0.226 | 0.141 | 0.386 |
| **ΔCaudal Middle Frontal Gyrus (RH)** | **ΔLeft Crus I** | 0.205 | 0.182 | 0.386 |
| **ΔCaudal Middle Frontal Gyrus (RH)** | **ΔRight Crus I** | 0.2 | 0.193 | 0.386 |
| **ΔCaudal Middle Frontal Gyrus (RH)** | **ΔLeft VIIIb** | 0.094 | 0.544 | 0.605 |
| **ΔCaudal Middle Frontal Gyrus (RH)** | **ΔLeft IX** | 0.096 | 0.535 | 0.605 |
| **ΔCaudal Middle Frontal Gyrus (RH)** | **ΔRight IX** | -0.182 | 0.237 | 0.394 |
| **ΔCaudal Middle Frontal Gyrus (RH)** | **ΔVermis X** | 0.23 | 0.133 | 0.386 |
| **ΔSuperior Frontal Gyrus (LH)** | **ΔRight V** | 0.128 | 0.409 | 0.612 |
| **ΔSuperior Frontal Gyrus (LH)** | **ΔLeft VI** | 0.188 | 0.221 | 0.612 |
| **ΔSuperior Frontal Gyrus (LH)** | **ΔVermis VI** | 0.121 | 0.434 | 0.612 |
| **ΔSuperior Frontal Gyrus (LH)** | **ΔRight VI** | 0.107 | 0.49 | 0.612 |
| **ΔSuperior Frontal Gyrus (LH)** | **ΔLeft Crus I** | -0.037 | 0.81 | 0.81 |
| **ΔSuperior Frontal Gyrus (LH)** | **ΔRight Crus I** | 0.045 | 0.774 | 0.81 |
| **ΔSuperior Frontal Gyrus (LH)** | **ΔLeft VIIIb** | -0.175 | 0.255 | 0.612 |
| **ΔSuperior Frontal Gyrus (LH)** | **ΔLeft IX** | -0.178 | 0.247 | 0.612 |
| **ΔSuperior Frontal Gyrus (LH)** | **ΔRight IX** | -0.152 | 0.324 | 0.612 |
| **ΔSuperior Frontal Gyrus (LH)** | **ΔVermis X** | -0.214 | 0.163 | 0.612 |
| **ΔSuperior Frontal Gyrus (RH)** | **ΔRight V** | 0.178 | 0.248 | 0.664 |
| **ΔSuperior Frontal Gyrus (RH)** | **ΔLeft VI** | 0.284 | 0.062 | 0.616 |
| **ΔSuperior Frontal Gyrus (RH)** | **ΔVermis VI** | 0.06 | 0.697 | 0.739 |
| **ΔSuperior Frontal Gyrus (RH)** | **ΔRight VI** | 0.16 | 0.298 | 0.664 |
| **ΔSuperior Frontal Gyrus (RH)** | **ΔLeft Crus I** | 0.067 | 0.663 | 0.739 |
| **ΔSuperior Frontal Gyrus (RH)** | **ΔRight Crus I** | 0.07 | 0.651 | 0.739 |
| **ΔSuperior Frontal Gyrus (RH)** | **ΔLeft VIIIb** | -0.192 | 0.212 | 0.664 |
| **ΔSuperior Frontal Gyrus (RH)** | **ΔLeft IX** | -0.097 | 0.533 | 0.739 |
| **ΔSuperior Frontal Gyrus (RH)** | **ΔRight IX** | -0.052 | 0.739 | 0.739 |
| **ΔSuperior Frontal Gyrus (RH)** | **ΔVermis X** | -0.15 | 0.332 | 0.664 |
| **ΔInferior Temporal Gyrus (LH)** | **ΔRight V** | -0.085 | 0.582 | 0.728 |
| **ΔInferior Temporal Gyrus (LH)** | **ΔLeft VI** | 0.202 | 0.189 | 0.632 |
| **ΔInferior Temporal Gyrus (LH)** | **ΔVermis VI** | 0.035 | 0.823 | 0.823 |
| **ΔInferior Temporal Gyrus (LH)** | **ΔRight VI** | -0.047 | 0.762 | 0.823 |
| **ΔInferior Temporal Gyrus (LH)** | **ΔLeft Crus I** | 0.091 | 0.558 | 0.728 |
| **ΔInferior Temporal Gyrus (LH)** | **ΔRight Crus I** | 0.117 | 0.451 | 0.728 |
| **ΔInferior Temporal Gyrus (LH)** | **ΔLeft VIIIb** | -0.268 | 0.079 | 0.632 |
| **ΔInferior Temporal Gyrus (LH)** | **ΔLeft IX** | -0.212 | 0.168 | 0.632 |
| **ΔInferior Temporal Gyrus (LH)** | **ΔRight IX** | -0.137 | 0.376 | 0.728 |
| **ΔInferior Temporal Gyrus (LH)** | **ΔVermis X** | 0.116 | 0.454 | 0.728 |
| **ΔInferior Temporal Gyrus (RH)** | **ΔRight V** | -0.028 | 0.857 | 0.937 |
| **ΔInferior Temporal Gyrus (RH)** | **ΔLeft VI** | 0.205 | 0.182 | 0.937 |
| **ΔInferior Temporal Gyrus (RH)** | **ΔVermis VI** | 0.035 | 0.821 | 0.937 |
| **ΔInferior Temporal Gyrus (RH)** | **ΔRight VI** | -0.063 | 0.683 | 0.937 |
| **ΔInferior Temporal Gyrus (RH)** | **ΔLeft Crus I** | 0.012 | 0.937 | 0.937 |
| **ΔInferior Temporal Gyrus (RH)** | **ΔRight Crus I** | 0.091 | 0.556 | 0.937 |
| **ΔInferior Temporal Gyrus (RH)** | **ΔLeft VIIIb** | -0.148 | 0.338 | 0.937 |
| **ΔInferior Temporal Gyrus (RH)** | **ΔLeft IX** | -0.129 | 0.404 | 0.937 |
| **ΔInferior Temporal Gyrus (RH)** | **ΔRight IX** | -0.074 | 0.632 | 0.937 |
| **ΔInferior Temporal Gyrus (RH)** | **ΔVermis X** | 0.036 | 0.814 | 0.937 |
| **ΔMiddle Temporal Gyrus (LH)** | **ΔRight V** | -0.003 | 0.986 | 0.989 |
| **ΔMiddle Temporal Gyrus (LH)** | **ΔLeft VI** | 0.189 | 0.218 | 0.834 |
| **ΔMiddle Temporal Gyrus (LH)** | **ΔVermis VI** | 0.015 | 0.924 | 0.989 |
| **ΔMiddle Temporal Gyrus (LH)** | **ΔRight VI** | -0.002 | 0.989 | 0.989 |
| **ΔMiddle Temporal Gyrus (LH)** | **ΔLeft Crus I** | 0.085 | 0.584 | 0.834 |
| **ΔMiddle Temporal Gyrus (LH)** | **ΔRight Crus I** | 0.086 | 0.577 | 0.834 |
| **ΔMiddle Temporal Gyrus (LH)** | **ΔLeft VIIIb** | -0.176 | 0.254 | 0.834 |
| **ΔMiddle Temporal Gyrus (LH)** | **ΔLeft IX** | -0.18 | 0.242 | 0.834 |
| **ΔMiddle Temporal Gyrus (LH)** | **ΔRight IX** | -0.097 | 0.53 | 0.834 |
| **ΔMiddle Temporal Gyrus (LH)** | **ΔVermis X** | -0.116 | 0.452 | 0.834 |
| **ΔMiddle Temporal Gyrus (RH)** | **ΔRight V** | 0.059 | 0.705 | 0.95 |
| **ΔMiddle Temporal Gyrus (RH)** | **ΔLeft VI** | 0.146 | 0.345 | 0.95 |
| **ΔMiddle Temporal Gyrus (RH)** | **ΔVermis VI** | -0.095 | 0.538 | 0.95 |
| **ΔMiddle Temporal Gyrus (RH)** | **ΔRight VI** | 0.041 | 0.791 | 0.95 |
| **ΔMiddle Temporal Gyrus (RH)** | **ΔLeft Crus I** | -0.01 | 0.95 | 0.95 |
| **ΔMiddle Temporal Gyrus (RH)** | **ΔRight Crus I** | 0.129 | 0.405 | 0.95 |
| **ΔMiddle Temporal Gyrus (RH)** | **ΔLeft VIIIb** | -0.101 | 0.513 | 0.95 |
| **ΔMiddle Temporal Gyrus (RH)** | **ΔLeft IX** | -0.065 | 0.673 | 0.95 |
| **ΔMiddle Temporal Gyrus (RH)** | **ΔRight IX** | -0.015 | 0.925 | 0.95 |
| **ΔMiddle Temporal Gyrus (RH)** | **ΔVermis X** | -0.081 | 0.602 | 0.95 |
| **ΔSuperior Temporal Gyrus (LH)** | **ΔRight V** | 0.174 | 0.259 | 0.937 |
| **ΔSuperior Temporal Gyrus (LH)** | **ΔLeft VI** | 0.146 | 0.344 | 0.937 |
| **ΔSuperior Temporal Gyrus (LH)** | **ΔVermis VI** | 0 | 0.998 | 0.998 |
| **ΔSuperior Temporal Gyrus (LH)** | **ΔRight VI** | 0.137 | 0.375 | 0.937 |
| **ΔSuperior Temporal Gyrus (LH)** | **ΔLeft Crus I** | 0.017 | 0.913 | 0.998 |
| **ΔSuperior Temporal Gyrus (LH)** | **ΔRight Crus I** | 0.245 | 0.11 | 0.937 |
| **ΔSuperior Temporal Gyrus (LH)** | **ΔLeft VIIIb** | -0.071 | 0.645 | 0.947 |
| **ΔSuperior Temporal Gyrus (LH)** | **ΔLeft IX** | -0.068 | 0.663 | 0.947 |
| **ΔSuperior Temporal Gyrus (LH)** | **ΔRight IX** | -0.08 | 0.604 | 0.947 |
| **ΔSuperior Temporal Gyrus (LH)** | **ΔVermis X** | -0.024 | 0.877 | 0.998 |
| **ΔSuperior Temporal Gyrus (RH)** | **ΔRight V** | -0.058 | 0.71 | 0.847 |
| **ΔSuperior Temporal Gyrus (RH)** | **ΔLeft VI** | 0.015 | 0.924 | 0.924 |
| **ΔSuperior Temporal Gyrus (RH)** | **ΔVermis VI** | -0.114 | 0.462 | 0.847 |
| **ΔSuperior Temporal Gyrus (RH)** | **ΔRight VI** | -0.073 | 0.638 | 0.847 |
| **ΔSuperior Temporal Gyrus (RH)** | **ΔLeft Crus I** | -0.094 | 0.544 | 0.847 |
| **ΔSuperior Temporal Gyrus (RH)** | **ΔRight Crus I** | 0.065 | 0.674 | 0.847 |
| **ΔSuperior Temporal Gyrus (RH)** | **ΔLeft VIIIb** | -0.179 | 0.245 | 0.847 |
| **ΔSuperior Temporal Gyrus (RH)** | **ΔLeft IX** | -0.133 | 0.39 | 0.847 |
| **ΔSuperior Temporal Gyrus (RH)** | **ΔRight IX** | -0.158 | 0.304 | 0.847 |
| **ΔSuperior Temporal Gyrus (RH)** | **ΔVermis X** | -0.047 | 0.763 | 0.847 |
| **ΔTransverse Temporal Gyrus (LH)** | **ΔRight V** | 0.167 | 0.279 | 0.92 |
| **ΔTransverse Temporal Gyrus (LH)** | **ΔLeft VI** | 0.059 | 0.702 | 0.92 |
| **ΔTransverse Temporal Gyrus (LH)** | **ΔVermis VI** | 0.086 | 0.578 | 0.92 |
| **ΔTransverse Temporal Gyrus (LH)** | **ΔRight VI** | 0.134 | 0.386 | 0.92 |
| **ΔTransverse Temporal Gyrus (LH)** | **ΔLeft Crus I** | -0.016 | 0.92 | 0.92 |
| **ΔTransverse Temporal Gyrus (LH)** | **ΔRight Crus I** | 0.178 | 0.247 | 0.92 |
| **ΔTransverse Temporal Gyrus (LH)** | **ΔLeft VIIIb** | -0.08 | 0.605 | 0.92 |
| **ΔTransverse Temporal Gyrus (LH)** | **ΔLeft IX** | -0.049 | 0.754 | 0.92 |
| **ΔTransverse Temporal Gyrus (LH)** | **ΔRight IX** | 0.021 | 0.893 | 0.92 |
| **ΔTransverse Temporal Gyrus (LH)** | **ΔVermis X** | -0.042 | 0.784 | 0.92 |
| **ΔTransverse Temporal Gyrus (RH)** | **ΔRight V** | 0.296 | 0.051 | 0.169 |
| **ΔTransverse Temporal Gyrus (RH)** | **ΔLeft VI** | 0.334 | 0.027 | 0.169 |
| **ΔTransverse Temporal Gyrus (RH)** | **ΔVermis VI** | 0.224 | 0.144 | 0.24 |
| **ΔTransverse Temporal Gyrus (RH)** | **ΔRight VI** | 0.298 | 0.049 | 0.169 |
| **ΔTransverse Temporal Gyrus (RH)** | **ΔLeft Crus I** | 0.259 | 0.09 | 0.18 |
| **ΔTransverse Temporal Gyrus (RH)** | **ΔRight Crus I** | 0.266 | 0.08 | 0.18 |
| **ΔTransverse Temporal Gyrus (RH)** | **ΔLeft VIIIb** | 0.057 | 0.715 | 0.795 |
| **ΔTransverse Temporal Gyrus (RH)** | **ΔLeft IX** | -0.006 | 0.969 | 0.969 |
| **ΔTransverse Temporal Gyrus (RH)** | **ΔRight IX** | -0.184 | 0.233 | 0.333 |
| **ΔTransverse Temporal Gyrus (RH)** | **ΔVermis X** | 0.084 | 0.587 | 0.733 |
| **ΔCaudal Anterior Cingulate Cortex (LH)** | **ΔRight V** | 0.243 | 0.112 | 0.52 |
| **ΔCaudal Anterior Cingulate Cortex (LH)** | **ΔLeft VI** | 0.062 | 0.69 | 0.893 |
| **ΔCaudal Anterior Cingulate Cortex (LH)** | **ΔVermis VI** | -0.002 | 0.992 | 0.992 |
| **ΔCaudal Anterior Cingulate Cortex (LH)** | **ΔRight VI** | 0.302 | 0.046 | 0.464 |
| **ΔCaudal Anterior Cingulate Cortex (LH)** | **ΔLeft Crus I** | -0.076 | 0.622 | 0.893 |
| **ΔCaudal Anterior Cingulate Cortex (LH)** | **ΔRight Crus I** | 0.057 | 0.714 | 0.893 |
| **ΔCaudal Anterior Cingulate Cortex (LH)** | **ΔLeft VIIIb** | 0.218 | 0.156 | 0.52 |
| **ΔCaudal Anterior Cingulate Cortex (LH)** | **ΔLeft IX** | 0.169 | 0.272 | 0.544 |
| **ΔCaudal Anterior Cingulate Cortex (LH)** | **ΔRight IX** | 0.177 | 0.25 | 0.544 |
| **ΔCaudal Anterior Cingulate Cortex (LH)** | **ΔVermis X** | -0.025 | 0.871 | 0.968 |
| **ΔCaudal Anterior Cingulate Cortex (RH)** | **ΔRight V** | 0.09 | 0.561 | 0.934 |
| **ΔCaudal Anterior Cingulate Cortex (RH)** | **ΔLeft VI** | 0.031 | 0.841 | 0.987 |
| **ΔCaudal Anterior Cingulate Cortex (RH)** | **ΔVermis VI** | -0.118 | 0.445 | 0.934 |
| **ΔCaudal Anterior Cingulate Cortex (RH)** | **ΔRight VI** | 0.212 | 0.168 | 0.817 |
| **ΔCaudal Anterior Cingulate Cortex (RH)** | **ΔLeft Crus I** | -0.183 | 0.236 | 0.817 |
| **ΔCaudal Anterior Cingulate Cortex (RH)** | **ΔRight Crus I** | 0.002 | 0.987 | 0.987 |
| **ΔCaudal Anterior Cingulate Cortex (RH)** | **ΔLeft VIIIb** | -0.006 | 0.968 | 0.987 |
| **ΔCaudal Anterior Cingulate Cortex (RH)** | **ΔLeft IX** | -0.022 | 0.886 | 0.987 |
| **ΔCaudal Anterior Cingulate Cortex (RH)** | **ΔRight IX** | -0.179 | 0.245 | 0.817 |
| **ΔCaudal Anterior Cingulate Cortex (RH)** | **ΔVermis X** | -0.11 | 0.478 | 0.934 |
| **ΔRostral Anterior Cingulate Cortex (LH)** | **ΔRight V** | -0.068 | 0.662 | 0.876 |
| **ΔRostral Anterior Cingulate Cortex (LH)** | **ΔLeft VI** | 0.07 | 0.652 | 0.876 |
| **ΔRostral Anterior Cingulate Cortex (LH)** | **ΔVermis VI** | -0.029 | 0.851 | 0.876 |
| **ΔRostral Anterior Cingulate Cortex (LH)** | **ΔRight VI** | -0.034 | 0.829 | 0.876 |
| **ΔRostral Anterior Cingulate Cortex (LH)** | **ΔLeft Crus I** | -0.042 | 0.787 | 0.876 |
| **ΔRostral Anterior Cingulate Cortex (LH)** | **ΔRight Crus I** | 0.041 | 0.79 | 0.876 |
| **ΔRostral Anterior Cingulate Cortex (LH)** | **ΔLeft VIIIb** | -0.093 | 0.549 | 0.876 |
| **ΔRostral Anterior Cingulate Cortex (LH)** | **ΔLeft IX** | 0.077 | 0.619 | 0.876 |
| **ΔRostral Anterior Cingulate Cortex (LH)** | **ΔRight IX** | 0.024 | 0.876 | 0.876 |
| **ΔRostral Anterior Cingulate Cortex (LH)** | **ΔVermis X** | -0.045 | 0.773 | 0.876 |
| **ΔRostral Anterior Cingulate Cortex (RH)** | **ΔRight V** | 0.045 | 0.77 | 0.925 |
| **ΔRostral Anterior Cingulate Cortex (RH)** | **ΔLeft VI** | 0.258 | 0.091 | 0.453 |
| **ΔRostral Anterior Cingulate Cortex (RH)** | **ΔVermis VI** | 0.302 | 0.046 | 0.453 |
| **ΔRostral Anterior Cingulate Cortex (RH)** | **ΔRight VI** | 0.126 | 0.415 | 0.894 |
| **ΔRostral Anterior Cingulate Cortex (RH)** | **ΔLeft Crus I** | 0.03 | 0.846 | 0.925 |
| **ΔRostral Anterior Cingulate Cortex (RH)** | **ΔRight Crus I** | 0.186 | 0.227 | 0.757 |
| **ΔRostral Anterior Cingulate Cortex (RH)** | **ΔLeft VIIIb** | 0.067 | 0.665 | 0.925 |
| **ΔRostral Anterior Cingulate Cortex (RH)** | **ΔLeft IX** | 0.05 | 0.747 | 0.925 |
| **ΔRostral Anterior Cingulate Cortex (RH)** | **ΔRight IX** | 0.015 | 0.925 | 0.925 |
| **ΔRostral Anterior Cingulate Cortex (RH)** | **ΔVermis X** | 0.118 | 0.447 | 0.894 |
| **ΔIsthmus Cingulate Cortex (LH)** | **ΔRight V** | 0.225 | 0.142 | 0.609 |
| **ΔIsthmus Cingulate Cortex (LH)** | **ΔLeft VI** | 0.084 | 0.589 | 0.736 |
| **ΔIsthmus Cingulate Cortex (LH)** | **ΔVermis VI** | 0.002 | 0.992 | 0.992 |
| **ΔIsthmus Cingulate Cortex (LH)** | **ΔRight VI** | 0.201 | 0.191 | 0.609 |
| **ΔIsthmus Cingulate Cortex (LH)** | **ΔLeft Crus I** | -0.006 | 0.967 | 0.992 |
| **ΔIsthmus Cingulate Cortex (LH)** | **ΔRight Crus I** | 0.15 | 0.331 | 0.662 |
| **ΔIsthmus Cingulate Cortex (LH)** | **ΔLeft VIIIb** | -0.114 | 0.459 | 0.736 |
| **ΔIsthmus Cingulate Cortex (LH)** | **ΔLeft IX** | -0.218 | 0.156 | 0.609 |
| **ΔIsthmus Cingulate Cortex (LH)** | **ΔRight IX** | -0.179 | 0.244 | 0.609 |
| **ΔIsthmus Cingulate Cortex (LH)** | **ΔVermis X** | 0.087 | 0.574 | 0.736 |
| **ΔIsthmus Cingulate Cortex (RH)** | **ΔRight V** | 0.402 | 0.007 | 0.059 |
| **ΔIsthmus Cingulate Cortex (RH)** | **ΔLeft VI** | 0.356 | 0.018 | 0.059 |
| **ΔIsthmus Cingulate Cortex (RH)** | **ΔVermis VI** | 0.048 | 0.757 | 0.757 |
| **ΔIsthmus Cingulate Cortex (RH)** | **ΔRight VI** | 0.359 | 0.017 | 0.059 |
| **ΔIsthmus Cingulate Cortex (RH)** | **ΔLeft Crus I** | 0.19 | 0.217 | 0.415 |
| **ΔIsthmus Cingulate Cortex (RH)** | **ΔRight Crus I** | 0.258 | 0.091 | 0.227 |
| **ΔIsthmus Cingulate Cortex (RH)** | **ΔLeft VIIIb** | 0.178 | 0.249 | 0.415 |
| **ΔIsthmus Cingulate Cortex (RH)** | **ΔLeft IX** | 0.072 | 0.641 | 0.736 |
| **ΔIsthmus Cingulate Cortex (RH)** | **ΔRight IX** | 0.068 | 0.663 | 0.736 |
| **ΔIsthmus Cingulate Cortex (RH)** | **ΔVermis X** | 0.079 | 0.609 | 0.736 |
| **ΔPosterior Cingulate Cortex (LH)** | **ΔRight V** | 0.461 | 0.002 | **0.016** |
| **ΔPosterior Cingulate Cortex (LH)** | **ΔLeft VI** | 0.174 | 0.258 | 0.432 |
| **ΔPosterior Cingulate Cortex (LH)** | **ΔVermis VI** | 0.074 | 0.633 | 0.703 |
| **ΔPosterior Cingulate Cortex (LH)** | **ΔRight VI** | 0.386 | 0.01 | 0.049 |
| **ΔPosterior Cingulate Cortex (LH)** | **ΔLeft Crus I** | 0.004 | 0.981 | 0.981 |
| **ΔPosterior Cingulate Cortex (LH)** | **ΔRight Crus I** | 0.16 | 0.299 | 0.432 |
| **ΔPosterior Cingulate Cortex (LH)** | **ΔLeft VIIIb** | 0.177 | 0.249 | 0.432 |
| **ΔPosterior Cingulate Cortex (LH)** | **ΔLeft IX** | 0.159 | 0.303 | 0.432 |
| **ΔPosterior Cingulate Cortex (LH)** | **ΔRight IX** | 0.259 | 0.089 | 0.298 |
| **ΔPosterior Cingulate Cortex (LH)** | **ΔVermis X** | 0.088 | 0.572 | 0.703 |
| **ΔPosterior Cingulate Cortex (RH)** | **ΔRight V** | 0.409 | 0.006 | **0.029** |
| **ΔPosterior Cingulate Cortex (RH)** | **ΔLeft VI** | 0.332 | 0.028 | 0.092 |
| **ΔPosterior Cingulate Cortex (RH)** | **ΔVermis VI** | 0.203 | 0.186 | 0.376 |
| **ΔPosterior Cingulate Cortex (RH)** | **ΔRight VI** | 0.414 | 0.005 | 0.029 |
| **ΔPosterior Cingulate Cortex (RH)** | **ΔLeft Crus I** | 0.146 | 0.345 | 0.388 |
| **ΔPosterior Cingulate Cortex (RH)** | **ΔRight Crus I** | 0.202 | 0.188 | 0.376 |
| **ΔPosterior Cingulate Cortex (RH)** | **ΔLeft VIIIb** | 0.168 | 0.274 | 0.388 |
| **ΔPosterior Cingulate Cortex (RH)** | **ΔLeft IX** | 0.145 | 0.349 | 0.388 |
| **ΔPosterior Cingulate Cortex (RH)** | **ΔRight IX** | 0.155 | 0.314 | 0.388 |
| **ΔPosterior Cingulate Cortex (RH)** | **ΔVermis X** | 0.046 | 0.765 | 0.765 |
| **ΔParahippocampal Gyrus (LH)** | **ΔRight V** | -0.413 | 0.005 | 0.054 |
| **ΔParahippocampal Gyrus (LH)** | **ΔLeft VI** | -0.166 | 0.282 | 0.403 |
| **ΔParahippocampal Gyrus (LH)** | **ΔVermis VI** | -0.108 | 0.484 | 0.605 |
| **ΔParahippocampal Gyrus (LH)** | **ΔRight VI** | -0.296 | 0.051 | 0.254 |
| **ΔParahippocampal Gyrus (LH)** | **ΔLeft Crus I** | -0.232 | 0.129 | 0.258 |
| **ΔParahippocampal Gyrus (LH)** | **ΔRight Crus I** | -0.235 | 0.125 | 0.258 |
| **ΔParahippocampal Gyrus (LH)** | **ΔLeft VIIIb** | -0.246 | 0.107 | 0.258 |
| **ΔParahippocampal Gyrus (LH)** | **ΔLeft IX** | -0.06 | 0.697 | 0.774 |
| **ΔParahippocampal Gyrus (LH)** | **ΔRight IX** | -0.203 | 0.187 | 0.311 |
| **ΔParahippocampal Gyrus (LH)** | **ΔVermis X** | 0.015 | 0.922 | 0.922 |
| **ΔParahippocampal Gyrus (RH)** | **ΔRight V** | -0.034 | 0.826 | 0.995 |
| **ΔParahippocampal Gyrus (RH)** | **ΔLeft VI** | 0.001 | 0.995 | 0.995 |
| **ΔParahippocampal Gyrus (RH)** | **ΔVermis VI** | 0.079 | 0.61 | 0.995 |
| **ΔParahippocampal Gyrus (RH)** | **ΔRight VI** | -0.003 | 0.987 | 0.995 |
| **ΔParahippocampal Gyrus (RH)** | **ΔLeft Crus I** | -0.028 | 0.857 | 0.995 |
| **ΔParahippocampal Gyrus (RH)** | **ΔRight Crus I** | 0.017 | 0.915 | 0.995 |
| **ΔParahippocampal Gyrus (RH)** | **ΔLeft VIIIb** | -0.047 | 0.764 | 0.995 |
| **ΔParahippocampal Gyrus (RH)** | **ΔLeft IX** | -0.083 | 0.591 | 0.995 |
| **ΔParahippocampal Gyrus (RH)** | **ΔRight IX** | -0.144 | 0.35 | 0.995 |
| **ΔParahippocampal Gyrus (RH)** | **ΔVermis X** | -0.136 | 0.38 | 0.995 |
| **ΔInsular Cortex (LH)** | **ΔRight V** | 0.011 | 0.945 | 0.945 |
| **ΔInsular Cortex (LH)** | **ΔLeft VI** | -0.079 | 0.611 | 0.764 |
| **ΔInsular Cortex (LH)** | **ΔVermis VI** | -0.121 | 0.435 | 0.725 |
| **ΔInsular Cortex (LH)** | **ΔRight VI** | -0.098 | 0.528 | 0.754 |
| **ΔInsular Cortex (LH)** | **ΔLeft Crus I** | -0.246 | 0.107 | 0.348 |
| **ΔInsular Cortex (LH)** | **ΔRight Crus I** | -0.032 | 0.836 | 0.928 |
| **ΔInsular Cortex (LH)** | **ΔLeft VIIIb** | -0.245 | 0.109 | 0.348 |
| **ΔInsular Cortex (LH)** | **ΔLeft IX** | -0.281 | 0.065 | 0.348 |
| **ΔInsular Cortex (LH)** | **ΔRight IX** | -0.227 | 0.139 | 0.348 |
| **ΔInsular Cortex (LH)** | **ΔVermis X** | -0.188 | 0.221 | 0.442 |
| **ΔInsular Cortex (RH)** | **ΔRight V** | 0.158 | 0.306 | 0.611 |
| **ΔInsular Cortex (RH)** | **ΔLeft VI** | 0.267 | 0.079 | 0.611 |
| **ΔInsular Cortex (RH)** | **ΔVermis VI** | 0.003 | 0.983 | 0.983 |
| **ΔInsular Cortex (RH)** | **ΔRight VI** | 0.159 | 0.304 | 0.611 |
| **ΔInsular Cortex (RH)** | **ΔLeft Crus I** | 0.066 | 0.67 | 0.838 |
| **ΔInsular Cortex (RH)** | **ΔRight Crus I** | 0.174 | 0.257 | 0.611 |
| **ΔInsular Cortex (RH)** | **ΔLeft VIIIb** | -0.072 | 0.642 | 0.838 |
| **ΔInsular Cortex (RH)** | **ΔLeft IX** | -0.084 | 0.586 | 0.838 |
| **ΔInsular Cortex (RH)** | **ΔRight IX** | -0.173 | 0.263 | 0.611 |
| **ΔInsular Cortex (RH)** | **ΔVermis X** | 0.006 | 0.97 | 0.983 |

Spearman partial correlations were computed between Δ cerebellar volume (mm³) and Δ cortical volume (mm³) in selected FSF-defined regions, adjusting for age, sex, education, APOE4 carrier status, and TIV. Statistical significance was determined at p < 0.05 (FDR-corrected q < 0.05).

Abbreviations: FSF, freesurfer; ROI, region of interest; ADD, Alzheimer’s disease dementia; APOE4, apolipoprotein E4; TIV, total intracranial volume; FDR, false discovery rate.

**Supplementary Table S14. Association between baseline Aβ burden and longitudinal cerebellar volume change in the PAD group**

| **Region** | **β** | **CI Lower** | **CI Upper** | **P value** | **FDR corrected Q value** | **R^2^** |
| --- | --- | --- | --- | --- | --- | --- |
| **Left I-V** | -0.132 | -0.367 | 0.103 | 0.266 | 0.92 | 0.146 |
| **Right I-V** | -0.064 | -0.304 | 0.175 | 0.592 | 0.92 | 0.178 |
| **Left V** | 0.018 | -0.236 | 0.272 | 0.888 | 0.92 | 0.088 |
| **Right V** | 0.119 | -0.137 | 0.374 | 0.355 | 0.92 | 0.202 |
| **Left VI** | -0.103 | -0.723 | 0.516 | 0.739 | 0.92 | 0.185 |
| **Vermis VI** | 0.116 | -0.017 | 0.248 | 0.085 | 0.92 | 0.179 |
| **Right VI** | 0.128 | -0.418 | 0.675 | 0.639 | 0.92 | 0.201 |
| **Left Crus I** | 0.145 | -1.363 | 1.652 | 0.848 | 0.92 | 0.153 |
| **Vermis Crus I** | 0.003 | -0.004 | 0.009 | 0.414 | 0.92 | 0.26 |
| **Right Crus I** | 0.302 | -0.638 | 1.242 | 0.522 | 0.92 | 0.207 |
| **Left Crus II** | 0.015 | -1.247 | 1.276 | 0.981 | 0.981 | 0.179 |
| **Vermis Crus II** | 0.028 | -0.043 | 0.099 | 0.427 | 0.92 | 0.335 |
| **Right Crus II** | 0.055 | -0.651 | 0.762 | 0.875 | 0.92 | 0.336 |
| **Left VIIb** | -0.106 | -0.751 | 0.539 | 0.743 | 0.92 | 0.168 |
| **Vermis VIIb** | 0.002 | -0.022 | 0.026 | 0.85 | 0.92 | 0.25 |
| **Right VIIb** | 0.074 | -0.357 | 0.506 | 0.73 | 0.92 | 0.298 |
| **Left VIIIa** | 0.169 | -0.453 | 0.79 | 0.588 | 0.92 | 0.103 |
| **Vermis VIIIa** | 0.136 | 0.013 | 0.259 | **0.03** | 0.852 | 0.329 |
| **Right VIIIa** | 0.086 | -0.367 | 0.54 | 0.704 | 0.92 | 0.19 |
| **Left VIIIb** | -0.048 | -0.552 | 0.457 | 0.85 | 0.92 | 0.085 |
| **Vermis VIIIb** | 0.075 | -0.017 | 0.168 | 0.107 | 0.92 | 0.183 |
| **Right VIIIb** | 0.23 | -0.134 | 0.594 | 0.211 | 0.92 | 0.167 |
| **Left IX** | 0.028 | -0.246 | 0.301 | 0.839 | 0.92 | 0.063 |
| **Vermis IX** | -0.016 | -0.105 | 0.072 | 0.717 | 0.92 | 0.211 |
| **Right IX** | 0.219 | -0.1 | 0.539 | 0.174 | 0.92 | 0.142 |
| **Left X** | -0.021 | -0.078 | 0.036 | 0.472 | 0.92 | 0.21 |
| **Vermis X** | -0.026 | -0.063 | 0.012 | 0.175 | 0.92 | 0.101 |
| **Right X** | 0.01 | -0.05 | 0.071 | 0.735 | 0.92 | 0.221 |

Linear regression models were constructed to examine whether baseline global cortical amyloid burden (SUVR) predicted longitudinal cerebellar volume change (Δ volume) in the PAD group. Each model included baseline regional cerebellar volume, age, sex, years of education, APOE4 carrier status, and TIV as covariates. For each cerebellar region, β coefficients, 95% CI, p-values, FDR–adjusted q-values, and model R² values are presented.

Abbreviations**:** Aβ, amyloid-β; SUVR, standardized uptake value ratio; Δ, change; CI, confidence interval; FDR, false discovery rate; TIV, total intracranial volume; PAD, preclinical Alzheimer’s disease.

**Supplementary Table S15. Association between baseline Aβ burden and longitudinal cerebellar volume change in the MCI_AD group**

| **Region** | **β** | **CI Lower** | **CI Upper** | **P value** | **FDR corrected Q value** | **R^2^** |
| --- | --- | --- | --- | --- | --- | --- |
| **Left I-V** | -0.074 | -0.234 | 0.086 | 0.36 | 0.926 | 0.195 |
| **Right I-V** | -0.086 | -0.268 | 0.096 | 0.352 | 0.926 | 0.139 |
| **Left V** | -0.063 | -0.237 | 0.11 | 0.47 | 0.926 | 0.1 |
| **Right V** | -0.044 | -0.268 | 0.18 | 0.696 | 0.926 | 0.211 |
| **Left VI** | -0.076 | -0.57 | 0.418 | 0.761 | 0.926 | 0.124 |
| **Vermis VI** | -0.024 | -0.116 | 0.069 | 0.612 | 0.926 | 0.091 |
| **Right VI** | -0.073 | -0.539 | 0.394 | 0.758 | 0.926 | 0.257 |
| **Left Crus I** | -0.147 | -0.948 | 0.653 | 0.716 | 0.926 | 0.17 |
| **Vermis Crus I** | -0.002 | -0.006 | 0.003 | 0.539 | 0.926 | 0.097 |
| **Right Crus I** | 0.246 | -0.527 | 1.019 | 0.529 | 0.926 | 0.163 |
| **Left Crus II** | -0.018 | -0.809 | 0.772 | 0.963 | 0.963 | 0.197 |
| **Vermis Crus II** | -0.009 | -0.054 | 0.036 | 0.686 | 0.926 | 0.246 |
| **Right Crus II** | -0.049 | -0.82 | 0.721 | 0.899 | 0.95 | 0.149 |
| **Left VIIb** | -0.039 | -0.496 | 0.417 | 0.865 | 0.95 | 0.12 |
| **Vermis VIIb** | -0.036 | -0.064 | -0.008 | **0.013** | 0.374 | 0.185 |
| **Right VIIb** | 0.023 | -0.411 | 0.457 | **0.916** | 0.95 | 0.175 |
| **Left VIIIa** | 0.143 | -0.208 | 0.495 | 0.42 | 0.926 | 0.078 |
| **Vermis VIIIa** | 0.034 | -0.057 | 0.125 | 0.462 | 0.926 | 0.183 |
| **Right VIIIa** | 0.159 | -0.237 | 0.555 | 0.428 | 0.926 | 0.103 |
| **Left VIIIb** | 0.066 | -0.194 | 0.326 | 0.617 | 0.926 | 0.146 |
| **Vermis VIIIb** | 0.008 | -0.054 | 0.07 | 0.798 | 0.931 | 0.18 |
| **Right VIIIb** | 0.295 | 0.001 | 0.59 | 0.05 | 0.54 | 0.198 |
| **Left IX** | 0.189 | -0.049 | 0.428 | 0.118 | 0.829 | 0.144 |
| **Vermis IX** | -0.036 | -0.116 | 0.043 | 0.364 | 0.926 | 0.201 |
| **Right IX** | 0.14 | -0.108 | 0.387 | 0.265 | 0.926 | 0.201 |
| **Left X** | 0.035 | -0.001 | 0.07 | 0.058 | 0.54 | 0.115 |
| **Vermis X** | 0.009 | -0.022 | 0.04 | 0.572 | 0.926 | 0.099 |
| **Right X** | 0.02 | -0.017 | 0.057 | 0.281 | 0.926 | 0.137 |

Linear regression models were constructed to examine whether baseline global cortical amyloid burden (SUVR) predicted longitudinal cerebellar volume change (Δ volume) in the MCI_AD group. Each model included baseline regional cerebellar volume, age, sex, years of education, APOE4 carrier status, and TIV as covariates. For each cerebellar region, β coefficients, 95% CI, p-values, FDR–adjusted q-values, and model R² values are presented.

Abbreviations**:** Aβ, amyloid-β; SUVR, standardized uptake value ratio; Δ, change; CI, confidence interval; FDR, false discovery rate; TIV, total intracranial volume; MCI_AD, mild cognitive impairment due to Alzheimer’s disease

**Supplementary Table S16. Association between baseline Aβ burden and longitudinal cerebellar volume change in the ADD group**

| **Region** | **β** | **CI Lower** | **CI Upper** | **P value** | **FDR corrected Q value** | **R^2^** |
| --- | --- | --- | --- | --- | --- | --- |
| **Left I-V** | 0.096 | -0.508 | 0.699 | 0.75 | 0.999 | 0.212 |
| **Right I-V** | -0.035 | -0.638 | 0.568 | 0.907 | 0.999 | 0.131 |
| **Left V** | -0.108 | -0.714 | 0.498 | 0.72 | 0.999 | 0.215 |
| **Right V** | -0.08 | -0.636 | 0.476 | 0.772 | 0.999 | 0.171 |
| **Left VI** | 0.087 | -1.068 | 1.242 | 0.879 | 0.999 | 0.327 |
| **Vermis VI** | -0.036 | -0.25 | 0.178 | 0.737 | 0.999 | 0.147 |
| **Right VI** | -0.044 | -1.135 | 1.048 | 0.936 | 0.999 | 0.244 |
| **Left Crus I** | -0.041 | -1.538 | 1.456 | 0.955 | 0.999 | 0.303 |
| **Vermis Crus I** | -0.01 | -0.02 | 0 | 0.051 | 0.999 | 0.313 |
| **Right Crus I** | -0.536 | -1.91 | 0.839 | 0.435 | 0.999 | 0.217 |
| **Left Crus II** | -0.054 | -1.591 | 1.482 | 0.943 | 0.999 | 0.182 |
| **Vermis Crus II** | 0.011 | -0.087 | 0.11 | 0.816 | 0.999 | 0.398 |
| **Right Crus II** | -0.118 | -1.49 | 1.255 | 0.863 | 0.999 | 0.202 |
| **Left VIIb** | 0.152 | -0.807 | 1.11 | 0.75 | 0.999 | 0.196 |
| **Vermis VIIb** | -0.031 | -0.088 | 0.025 | 0.273 | 0.999 | 0.131 |
| **Right VIIb** | 0.24 | -0.708 | 1.187 | 0.611 | 0.999 | 0.247 |
| **Left VIIIa** | 0.001 | -1.044 | 1.045 | 0.999 | 0.999 | 0.213 |
| **Vermis VIIIa** | 0.111 | -0.12 | 0.342 | 0.335 | 0.999 | 0.219 |
| **Right VIIIa** | 0.329 | -0.571 | 1.229 | 0.463 | 0.999 | 0.134 |
| **Left VIIIb** | 0.019 | -0.9 | 0.938 | 0.967 | 0.999 | 0.106 |
| **Vermis VIIIb** | -0.04 | -0.159 | 0.078 | 0.493 | 0.999 | 0.161 |
| **Right VIIIb** | 0.117 | -0.488 | 0.722 | 0.698 | 0.999 | 0.164 |
| **Left IX** | 0.138 | -0.344 | 0.62 | 0.565 | 0.999 | 0.168 |
| **Vermis IX** | -0.027 | -0.197 | 0.142 | 0.744 | 0.999 | 0.36 |
| **Right IX** | -0.103 | -0.579 | 0.373 | 0.662 | 0.999 | 0.351 |
| **Left X** | -0.02 | -0.106 | 0.066 | 0.641 | 0.999 | 0.237 |
| **Vermis X** | 0 | -0.073 | 0.072 | 0.991 | 0.999 | 0.122 |
| **Right X** | -0.025 | -0.099 | 0.05 | 0.509 | 0.999 | 0.179 |

Linear regression models were constructed to examine whether baseline global cortical amyloid burden (SUVR) predicted longitudinal cerebellar volume change (Δ volume) in the ADD group. Each model included baseline regional cerebellar volume, age, sex, years of education, APOE4 carrier status, and TIV as covariates. For each cerebellar region, β coefficients, 95% CI, p-values, FDR–adjusted q-values, and model R² values are presented.

Abbreviations: Aβ, amyloid-β; SUVR, standardized uptake value ratio; Δ, change; CI, confidence interval; FDR, false discovery rate; TIV, total intracranial volume; ADD, Alzheimer’s disease dementia

**Supplementary Data**

**Supplementary Data 1. Analysis codes(python scripts)**

**Code list:**

1. cerebellum_merge_preprocess.py — calculates delta values and merges group files.

2. gee_analysis.py — performs GEE analysis controlling for covariates (age, sex, APOE4, TICV, education).

3. ancova_posthoc_plot.py — runs ANCOVA and post-hoc comparisons for regional cerebellar changes.

4. partial_corr_residuals.py — partial correlation between cerebellar and cortical regions using residuals.

5. plot_results.py — generates bar plots, scatter plots, and annotated significance.

All scripts were written and executed using Python 3.11 and standard packages (pandas, statsmodels, scipy, matplotlib).

**Supplementary References**

1. Lee JH, Lee KU, Lee DY, et al. Development of the Korean version of the Consortium to Establish a Registry for Alzheimer's Disease Assessment Packet (CERAD-K): clinical and neuropsychological assessment batteries. J Gerontol B Psychol Sci Soc Sci. 2002;57(1):P47-P53.
2. Cummings JL, Mega M, Gray K, Rosenberg-Thompson S, Carusi DA, Gornbein J. The Neuropsychiatric Inventory: comprehensive assessment of psychopathology in dementia. Neurology. 1994;44(12):2308-2314.
3. Jack CR Jr, Bennett DA, Blennow K, et al. NIA-AA research framework: toward a biological definition of Alzheimer's disease. Alzheimers Dement. 2018;14(4):535-562.
4. Morris JC. Clinical dementia rating: a reliable and valid diagnostic and staging measure for dementia of the Alzheimer type. Int Psychogeriatr. 1997;9(Suppl 1):173-176; discussion 177-178.
5. Petersen RC, Caracciolo B, Brayne C, Gauthier S, Jelic V, Fratiglioni L. Mild cognitive impairment: a concept in evolution. J Intern Med. 2014;275(3):214-228.
6. Li X, Morgan PS, Ashburner J, Smith J, Rorden C. The first step for neuroimaging data analysis: DICOM to NIfTI conversion. J Neurosci Methods. 2016;264:47-56.
7. Lee J, Ha S, Kim REY, Lee M, Kim D, Lim HK. Development of amyloid PET analysis pipeline using deep learning-based brain MRI segmentation—A comparative validation study. Diagnostics (Basel). 2022;12(3):623.
8. Thurfjell L, Lilja J, Lundqvist R, et al. Automated quantification of 18F-flutemetamol PET activity for categorizing scans as negative or positive for brain amyloid: concordance with visual image reads. J Nucl Med. 2014;55(10):1623-1628.
9. Bao YW, Chau ACM, Chiu PK, et al. Heterogeneity of amyloid binding in cognitively impaired patients consecutively recruited from a memory clinic: evaluating the utility of quantitative 18F-flutemetamol PET-CT in discrimination of mild cognitive impairment from Alzheimer's disease and other dementias. J Alzheimers Dis. 2021;79(2):819-832.
10. Lee H, Kim HW, Lee M, et al. Evaluating brain volume segmentation accuracy and reliability of FreeSurfer and Neurophet AQUA at variations in MRI magnetic field strengths. Sci Rep. 2024;14(1):24513.
11. Fischl B. FreeSurfer. Neuroimage. 2012;62(2):774-781.11. Lee H, Kim HW, Lee M, et al. Evaluating brain volume segmentation accuracy and reliability of FreeSurfer and Neurophet AQUA at variations in MRI magnetic field strengths. *Scientific Reports*. 2024;14(1):24513.
12. Kesslak JP, Nalcioglu O, Cotman CW. Quantification of magnetic resonance scans for hippocampal and parahippocampal atrophy in Alzheimer's disease. Neurology. 1991;41(1):51.
13. Zhu Y, Wu Y, Lv X, et al. The relationship between APOE genotype, CSF Tau and cognition across the Alzheimer's disease spectrum, moderation and mediation role of insula network connectivity. CNS Neurosci Ther. 2024;30(1):e14401.
14. Fennema-Notestine C, Hagler DJ Jr, McEvoy LK, et al. Structural MRI biomarkers for preclinical and mild Alzheimer's disease. Hum Brain Mapp. 2009;30(10):3238-3253.
15. Liu H, Zhang L, Xi Q, et al. Changes in brain lateralization in patients with mild cognitive impairment and Alzheimer's disease: a resting-state functional magnetic resonance study from Alzheimer's Disease Neuroimaging Initiative. Front Neurol. 2018;9:3.
16. Miller J, Watrous AJ, Tsitsiklis M, et al. Lateralized hippocampal oscillations underlie distinct aspects of human spatial memory and navigation. Nat Commun. 2018;9(1):2423.
17. Tyrer A, Gilbert JR, Adams S, et al. Lateralized memory circuit dropout in Alzheimer’s disease patients. Brain Commun. 2020;2(2):fcaa134.
18. Gaser C, Dahnke R, Thompson P, Kurth F, Luders E. CAT – a computational anatomy toolbox for the analysis of structural MRI data. bioRxiv. 2022. doi:10.1101/2022.06.11.495771.
19. Diedrichsen J. A spatially unbiased atlas template of the human cerebellum. Neuroimage. 2006;33(1):127-138.
20. Na S, Seo SW, Kim YJ, Yoo H, Lee ES. Correlation analysis between subtest scores of CERAD-K and a newly developed tablet computer-based digital cognitive test (Inbrain CST). Front Aging Neurosci. 2023;15:1178324.
21. Hiu SKW, Bigirumurame T, Kunonga P, Bryant A, Pillai M. Neuropsychiatric Inventory domains cluster into neuropsychiatric syndromes in Alzheimer's disease: a systematic review and meta-analysis. Brain Behav. 2022;12(9):e2734.
